# Dynamics-informed machine learning for recovering extensive missing systems dynamics

**DOI:** 10.1038/s41467-026-77922-1

**Published:** 2026-09-19

**Authors:** Tao Wu, Xiangyun Gao, Ying Tang, Feng An, Xiaotian Sun, Kazuyuki Aihara, Haizhong An, Jürgen Kurths

**Affiliations:** 1 College of Management Science, Chengdu University of Technology, Chengdu, China; 2 International Research Center for Neurointelligence (IRCN), The University of Tokyo, Tokyo, Japan; 3 School of Economics and Management, China University of Geosciences, Beijing, China; 4 MOE Social Science Laboratory of Mineral Resources Security Governance, China University of Geosciences, Beijing, China; 5 Institute of Fundamental and Frontier Sciences, University of Electronic Science and Technology of China, Chengdu, China; 6 Key Laboratory of Quantum Physics and Photonic Quantum Information, Ministry of Education, University of Electronic Science and Technology of China, Chengdu, China; 7 School of Economics and Management, Beijing University of Chemical Technology, Beijing, China; 8 School of Advanced Manufacturing and Robotics, Peking University, Beijing, China; 9 Center for Mineral Industrial Chains, Hebei GEO University, Shijiazhuang, China; 10 Potsdam Institute for Climate Impact Research (PIK)–Member of the Leibniz Association, Potsdam, Germany

**Keywords:** Computational science, Nonlinear phenomena

## Abstract

Inevitable missing values in observational time series often hinder reliable data-driven modeling of complex systems across diverse domains. Recovery is essential yet challenging, particularly in high-dimensional systems where most variables can exhibit extensive intra-series missingness and widespread inter-variable missingness simultaneously. In this study, we introduce a dynamics-informed imputation framework, termed Full-Partial Reconstruction Mapping (FPRM). In contrast to traditional approaches that directly fit time series interdependences, the FPRM embeds intrinsic dynamical priors via state-space reconstruction and recovers multiple incomplete series by learning diffeomorphic topology between attractors from complete and incomplete data. We validate the FPRM on paradigmatic model systems (e.g., an ecology system, the ordinary Lorenz system, a coupled 120-dimensional (120D) Lorenz system, and a coupled 120D Rössler system and real-world datasets (e.g., road occupancy rate from transportation system, electroencephalogram (EEG) signals from neuroscience, exchange rates from finance, and wind speed from climate systems). Even in high-dimensional systems where only one variable is fully observed and all others suffer from extreme missingness (e.g., 90%), the FPRM enables reliable imputation of multiple incomplete variables—a task that challenges most existing approaches. This dynamics-guided framework outperforms traditional approaches, alleviating substantial data gaps and enabling robust data-driven paradigms for complex dynamical systems.

## Abstract (web summary)

Here, the authors introduce a dynamics-informed framework for imputing missing values in high-dimensional observational time series. They demonstrate that FPRM can reliably reconstruct severely incomplete variables and outperform conventional approaches across synthetic and real-world datasets

## Introduction

**Fig. 1:**
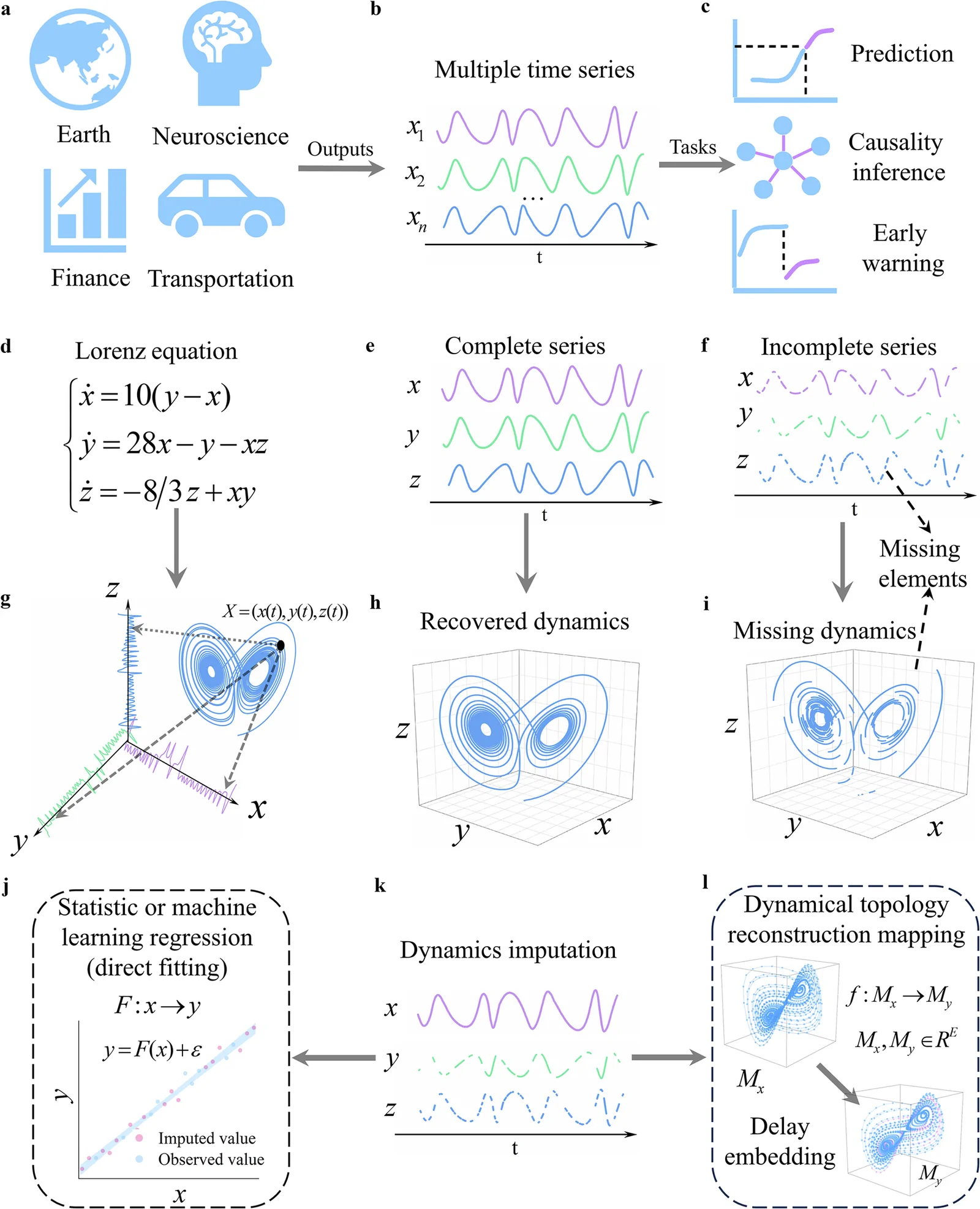
Time series and missing dynamics in complex systems. **a**–**c** Accessible multi-domain time series serve as the foundation for data-driven scientific discovery and decision-making across various real-world complex systems. **d** In the ordinary Lorenz system, the dynamical evolution is governed by a set of coupled differential equations. **g** Its dynamical trajectory in state space can be projected onto the coordinate axes of individual variables, yielding multiple time series. **e**, **h** Observed time series of the interacting variables can be mapped to the multi-dimensional state space to recover the trajectory. **f**, **i** Missing values in observed time series undermine the accurate approximation of underlying system states, leading to missing dynamics. **j**, **k** Traditional statistical and machine learning regression methods realize imputation by directly fitting correlations between complete (e.g., *x*) and incomplete time series (*y*). **k**, **l** Our imputation framework learns a one-to-one mapping between multidimensional manifolds (e.g., ${M}_{x}$ and ${M}_{y}$) reconstructed from complete and incomplete time series, respectively. In light of the delay embedding theorem, these reconstructed manifolds encode the fundamental dynamics (e.g., evolution trajectories in the state space) of the underlying system.

Complex dynamical systems are ubiquitous across real-world domains, such as climate, ecology, finance, and neuroscience. These systems have motivated extensive research aimed at decoding their underlying dynamical rules to enable predictive modeling and evidence-based decision-making^1–4^. Theoretically, the dynamics of such systems can be represented by appropriately formulated differential equations, which have inspired the development of numerous equation-based models^5,6^. However, these equation-driven approaches often face inherent limitations in real-world systems due to high dimensionality and intricate nonlinearity, which hinder the derivation and validation of accurate mathematical formulations^7–9^. Data-driven approaches have emerged as promising alternatives for the study of complex systems^10^. A defining feature of complex systems is their generation of rich time-series data from interacting system components^11^. Coupled with advances in computational capabilities and state-of-the-art techniques (e.g., machine learning (ML) or artificial intelligence (AI)), has unlocked new avenues for data-driven discovery in the field^12–14^. Notably, in scenarios where experimental manipulation is prohibitively costly, ethically questionable, or practically impossible, such as in climate systems, medicine and neuroscience, data-driven modeling is useful and essential for addressing fundamental tasks, including causal inference, forecasting, and early warning monitoring (Fig. 1a–c)^15–20^.

Various state-of-the-art ML/AI approaches are purely data-driven without prior knowledge of their underlying systems^21–23^. For instance, in general climate prediction tasks, extensive observational features (e.g., hourly climate data, such as temperature, humidity, and wind speed) are directly fed into a structured neural network, and the outputs serve as predictions with no incorporation of domain-specific prior knowledge (e.g., physical laws of the climate system)^24^. Consequently, the quality of the input data is of critical importance, as high-quality data ensures the generalizability and reliability of trained models, whereas training with low-quality data (e.g., incomplete datasets) may yield biased or even erroneous results, potentially leading to inappropriate decisions^16^. However, incomplete data with missing entries are inevitable in practical applications, such as climate time series derived from satellite observations that may contain missing values owing to cloud occlusion or sensor malfunctions^16^, whereas ecological time series suffer from data gaps caused by adverse weather conditions, seasonal variations, or sampling equipment failures^25^. Missing values may distort the overall statistical distribution of datasets^26^. Furthermore, time series observations are intrinsically linked to the underlying dynamics (e.g., the system’s evolution trajectories in the state space) of complex systems. Missing values in observed time series can pose an obstacle to recovering historical system states and their associated trajectory information^27^. For illustration, consider the classic Lorenz system, a prototypical simplified model of fluid dynamics^28^. Its dynamical flow evolves over time to form a butterfly-like structure in the state space, and system states are directly projected onto the three observed time‑series variables (Fig. 1d, g). Consequently, these time series can inversely approximate the historical states of the underlying system (Fig. 1e, h), and such a reliable reconstruction will facilitate various subsequent downstream dynamical analysis tasks (e.g., dynamical behavior prediction). However, when time‑series data contain substantial missing entries, our capacity to reconstruct these historical system states and trajectory information deteriorates (Fig. 1f, i). Therefore, imputing missing values in time series is of great importance for the analysis of dynamical systems.

**Table 1:** Comparison with other imputation techniques

Dataset	Method											
	FPRM	Mean	*k*-NN	GPR	SVM	RF	NN	SVM^*^	NN^*^	MVCM	S-map	MetaTSI
120D Lorenz system	(0.89,0.07) (0.41,0.13)	(0.01,0.09) (1.11,0.18)	(0.64,0.05) (0.82,0.06)	(0.71,0.06) (0.57,0.14)	(0.7,0.11) (0.69,0.1)	(0.51,0.05) (0.82,0.14)	(0.74,0.08) (0.65,0.11)	(0.81,0.08) (0.53,0.1)	(0.82,0.07) (0.49,0.16)	(0.63,0.1) (0.67,0.09)	(0.27,0.04) (1.13,0.23)	(0.72,0.07) (0.68,0.09)
120D Rossler system	(0.9,0.14) (0.31,0.21)	(0.01,0.04) (1.07,0.15)	(0.66,0.07) (0.81,0.12)	(0.51,0.08) (0.67,0.11)	(0.49,0.08) (0.67,0.09)	(0.46,0.06) (0.79,0.11)	(0.46,0.05) (0.69,0.18)	(0.84,0.07) (0.44,0.09)	(0.87,0.1) (0.39,0.22)	(0.64,0.12) (0.68,0.08)	(0.26,0.05) (1.12,0.17)	(0.87,0.09) (0.49,0.14)
500D road occupancy rates	(0.83,0.08) (0.49,0.13)	(0.01,0.03) (1.01,0.07)	(0.61,0.07) (0.86,0.11)	(0.67,0.11) (0.68,0.16)	(0.64,0.15) (0.69,0.16)	(0.53,0.12) (0.89,0.13)	(0.68,0.07) (0.87,0.12)	(0.77,0.1) (0.65,0.14)	(0.7,0.11) (0.76,0.21)	(0.6,0.11) (0.66,0.1)	(0.25,0.06) (1.22,0.39)	(0.75,0.14) (0.66,0.08)
64D EEG series	(0.95,0.11) (0.27,0.12)	(0.01,0.02) (1.07,0.12)	(0.06,0.11) (1.36,0.18)	(0.66,0.1) (0.71,0.16)	(0.62,0.12) (0.75,0.13)	(0.68,0.09) (0.85,0.17)	(0.64,0.16) (0.85,0.12)	(0.91,0.09) (0.38,0.13)	(0.89,0.12) (0.39,0.23)	(0.58,0.16) (0.71,0.13)	(0.22,0.06) (1.32,0.28)	(0.12,0.14) (1.01,0.11)
52D exchange rates	(0.82,0.14) (0.41,0.24)	(−0.01,0.1) (1.03,0.09)	(0.71,0.12) (0.68,0.18)	(0.79,0.09) (0.43,0.15)	(0.79,0.12) (0.52,0.14)	(0.65,0.15) (0.77,0.21)	(0.78,0.1) (0.46,0.16)	(0.76,0.1) (0.48,0.21)	(0.78,0.15) (0.51,0.13)	(0.51,0.14) (0.79,0.18)	(0.24,0.04) (1.3,0.27)	(0.86,0.01) (0.31,0.06)

Note: For each dataset, we assume that the first variable is fully observed, whereas all other variables are incomplete, with 90% missing values. SVM^*^ and NN^*^ denote that the training processes of FPRM are replaced by support vector machine (SVM) and neural network (NN), respectively. The machine learning algorithms and neural networks are simulated using the Statistics and Machine Learning Toolbox in MATLAB R2024b. Each entry reports average ρ and RMSE, their standard deviations across all imputed incomplete variables.

Practically, while datasets with sparse missing values may be addressed via deletion (e.g., time points containing missing values are removed for temporal alignment across variables), imputation has emerged as a widely embraced alternative for managing non-ignorable missing values^29^. Certain basic approaches to imputing missing values in time series leverage their distributional properties, for instance, replacing each missing value with the mean or median of all non-missing values for the corresponding variable^30^. Alternative approaches achieve imputation by learning statistical associations (e.g., the linear/nonlinear coherence between fully observed series and incomplete series^31^, Fig. 1j, k) or intrinsic patterns (e.g., long-range interdependences^32^) within the data. These methods leverage traditional machine learning algorithms^31^ (e.g., random forest^33^, support vector machine^34^, Gaussian process regression^35^), advanced neural networks^36–39^ (e.g., recurrent neural network^40^), and their extensions^41,42^. To further refine filling accuracy, researchers shift modeling paradigms to encode the fundamental dynamics behind time series. Specifically, S-map and its multi-view extension MVCM^11,25^ are classic empirical dynamic modeling tools that operate well without prior knowledge of system governing equations and achieve credible gap-filling results for ecological time series with mild missing entries. Meanwhile, the meta-learning framework MetaTSI^43^ provides a specialized dynamics-aware imputation pipeline to characterize intricate spatiotemporal evolution in urban traffic data. Though these methods yield satisfying performance within their respective domain boundaries, accurately recovering missing dynamics of complex systems under high missing rates remains an open area for improvement.

(i) Real-world systems are inherently nonlinear, with their output time series often exhibiting nonlinearity, chaos, and noise^44^. These properties render traditional statistical imputation methods ill-suited because most rely on strong assumptions regarding stationary, low-dimensional data, and fail to capture the dynamic complexity of time-evolving systems. (ii) For time series with a high proportion of missing values (high intra-series missingness, e.g., 80% or 90%), distribution-based methods may struggle because of a disrupted data distribution. Traditional machine learning models or neural networks may be constrained either by scarce usable training samples or by their propensity for overfitting^10^. iii) Real-world systems typically comprise multiple interacting components and exhibit high dimensionality. In scenarios where most system components contain missing values (widespread inter-variable missingness), e.g., when over 90% of the climate observations from distinct geographic locations have missing values, the number of available input features for imputation is considerably reduced. This challenge is further exacerbated when most variables have a high proportion of missing values. In addition, neural network–based imputation methods often require a careful balance between performance, computational cost, and interpretability^19^.

To address these challenges, we draw core methodological inspiration from dynamics-aware time-delay embedding frameworks, including S-map^11^ and MVCM^25^. S-map achieves dynamic imputation via weighted local linear approximation restricted to neighbors within a single attractor embedding view^11^, while MVCM extends S-map by ensembling predictions from multiple dynamically relevant attractor views weighted by cross-mapping skill^25^. Both methods deliver competitive imputation performance for ecological time series. Distinct from the pointwise local-neighbor fitting paradigm of S-map and MVCM, our FPRM leverages the intrinsic diffeomorphic topological correspondence between attractor manifolds guaranteed by Takens’ embedding theorem, and approximates this native manifold-to-manifold relation via Gaussian process regression (GPR)^45^. Specifically, FPRM first reconstructs attractor embeddings to encode system dynamics based on the delay embedding theorem^46^: a full-state reconstruction from fully observed time series, and a partial-state reconstruction from incomplete observations (Fig. 1k, l). We then train the GPR model on paired valid state samples spanning the entire domain of both manifolds to approximate their inherent topological mapping. This pipeline enables theoretically grounded recovery of missing dynamical trajectories by capitalizing on topological invariants retained during delay-coordinate embedding. Crucially, the diffeomorphism is an inherent topological property of the paired reconstructed manifolds, rather than a characteristic imposed by the GPR fit. The GPR merely acts as a flexible nonparametric surrogate to retrieve unobserved states based on this pre-existing manifold structure (see *Materials and “Methods”*). We first validate our framework across multiple benchmark model systems covering both low-dimensional dynamics (e.g., the 3-dimensional (3D) ecological system and 3D Lorenz system) and high-dimensional setups (e.g., the coupled 120D Lorenz system and 120D coupled Rössler system). Beyond the model systems, the efficacy of the FPRM framework is further demonstrated using various real-world datasets from transportation, neuroscience, finance, and climate.

## Results

### Full and partial reconstruction from complete and incomplete time series

**Fig. 2:**
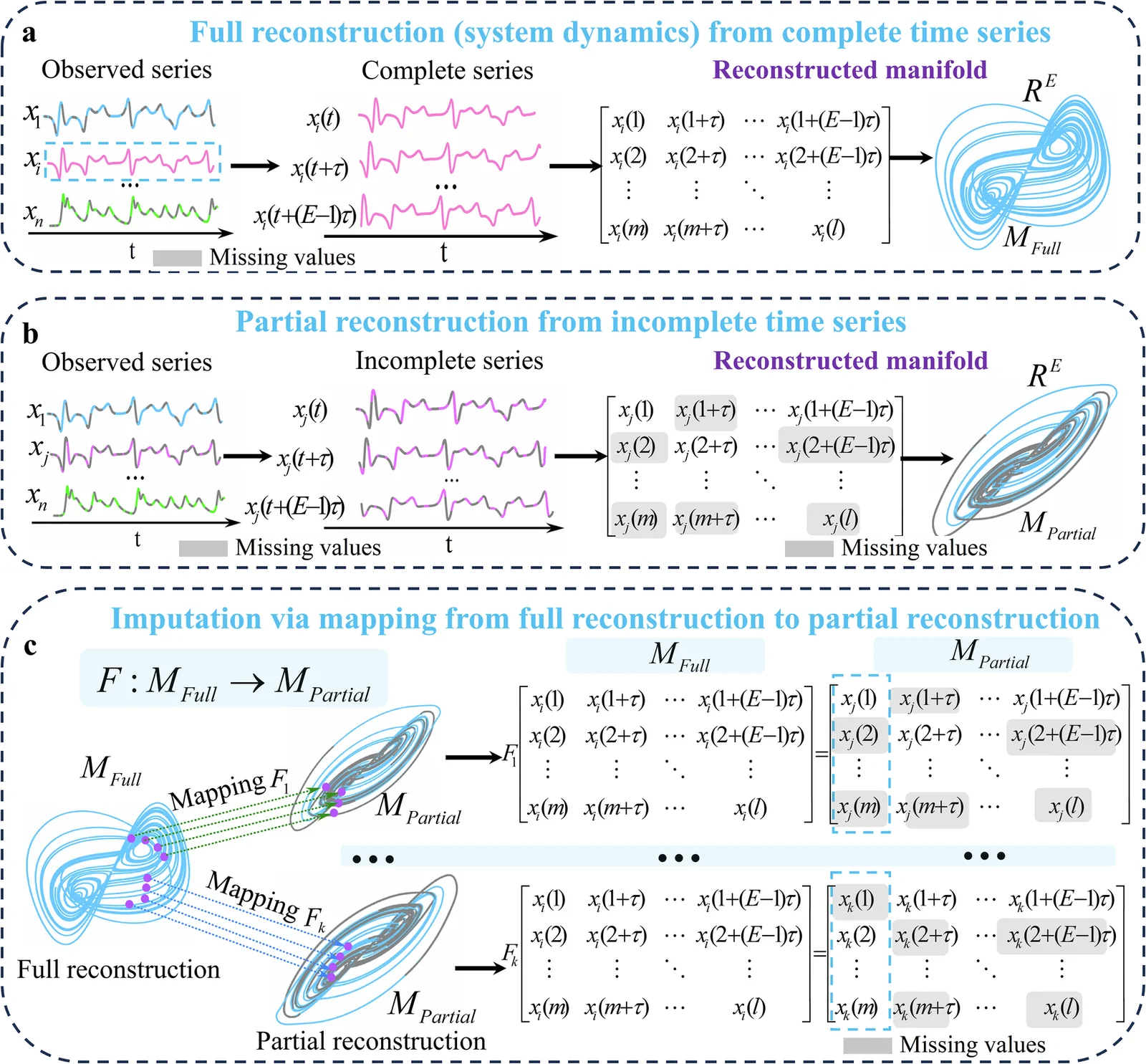
Schematic illustration of the FPRM framework. **a** From a complete series, an attractor manifold (system dynamics) (exemplified by the Lorenz attractor) is reconstructed in an *E*-dimensional state space, where all state information is known (full reconstruction). **b** On the other hand, from an incomplete series, an attractor manifold (partial reconstruction) is generated within the same state space, but with certain state information missing (depicted as the gray area). The missing elements precisely correspond to the missing values in the incomplete series. Analogously, each incomplete time series can yield a partial reconstruction. **c** Based on the embedding theory, an inherent one-to-one mapping exists between the full reconstruction and the partial reconstruction (especially for the first column vector). This mapping enables the imputation of all missing states in the partial reconstruction. Subsequently, through multiple such mappings from the full reconstruction to all partial reconstructions, the imputation of all incomplete time series within a complex system can be accomplished.

An *n*-dimensional dynamical system evolves temporally, tracing a state trajectory (which lies on the associated attractor manifold *M*) in the state space and concurrently generating *n* time-series variables (Fig. 2a). These observational time series form the foundation for subsequent investigations, because the underlying governing rules are unknown in most practical systems. When the state of observations at sequent time *t* (t=1,2,…,l) is fully observed, the time series x(t) is considered complete. By contrast, an incomplete time series exhibits missing values for certain time instants ($t\in \{1,2,\ldots,l\}$). Our primary objective is to impute all missing values for each incomplete time series in complex dynamical systems.

Many real-world systems are inherently modeled as dynamical systems, and the associated field of study is called dynamical systems theory. Takens’ embedding theorem, a pivotal concept in this theory, has been extensively applied, particularly in the analysis of nonlinear time series^47^. According to the embedding, each time series variable can be utilized to approximately reconstruct the dynamical trajectory of the underlying systems^46^ (see examples in Supplementary Fig. S1 and Supplementary Fig. S2). In particular, from a complete series ${x}_{i}(t)$($i\in \{1,2,\ldots,n\}$), by selecting an appropriate embedding dimension *E* and time delay τ (the selection of these parameters in this study is provided in Supplementary Fig. S1), we can reconstruct an attractor manifold ${M}_{Full}$ with the full state information (full reconstruction) (Fig. 2a). This reconstruction ${M}_{Full}$ demonstrates a diffeomorphic correspondence with the underlying dynamical system. Similarly, a new reconstruction ${M}_{Partial}$ can be also obtained from an incomplete series ${x}_{j}(t)$ ($j\in \{1,2,\ldots,l\}$,j≠i). According to Takens’ theorem, time-series-based reconstructions exhibit diffeomorphic topology with the underlying system, theoretically implying a one-to-one mapping between the reconstructions and the underlying system^46^.

1 $$f:M\to {M}_{{Full}},\,g:M\to {M}_{{Partial}}$$

Note that certain states on ${M}_{Partial}$ are unknown (gray units in Fig. 2b) because of missing values in the original time series; thus, this reconstruction only recovers the partial dynamics of the underlying system (partial reconstruction). Although partial reconstruction involves missing states, it should be diffeomorphic to the underlying system once these missing states are recovered; this consistency aligns with Takens’ embedding theorem^46^.

### Imputation via mapping from full reconstruction to partial reconstruction

Full and partial reconstructions are obtained via delay-coordinate transformations of the complete and incomplete time series, respectively. As both functions *f* and *g* are diffeomorphic mappings, we can deduce a one-to-one mapping from the full to partial reconstructions.

2 $$F:{M}_{Full}\to {M}_{Partial}$$

where $$F(x)=g({f}^{-1}(x))$$.

Thus, these missing values on ${M}_{Partial}$ can be recovered by identifying the mapping *F* (Fig. 2c). Specifically, the corresponding known values of the full and partial reconstructions are employed as training inputs to identify mapping *F*. Subsequently, the known values of the full reconstruction that are contemporary with the missing values serve as new inputs to impute all missing values in the partial reconstruction. In this study, we employ the classic Gaussian process regression (GPR) algorithm to train the mapping *F* (see *Materials and Methods*) owing to its robust capability in handling even small sample sizes and the uncertainty inherent in real-world datasets^45^.

Because every incomplete time series can fundamentally yield a partial reconstruction of the underlying system, imputation can be achieved by establishing multiple mappings, each linking a full reconstruction to its corresponding partial reconstruction. The proposed approach enables an efficient handling of imputation tasks for multivariate time series, even in challenging scenarios where the majority of observed time series in high-dimensional systems contain missing values.

To simulate missing values in both model-derived and real-world systems, elements are incrementally and randomly removed from a specific time-series variable, with the proportion of missing values ranging from 10% to 90%. The performance is measured by comparing the imputed values with the original observed values. Two metrics are employed for a quantitative assessment: i) the Pearson correlation coefficient (ρ) between the observed and imputed data, and ii) the normalized root mean square error (RMSE).

### Validation in low-dimensional model systems

**Fig. 3:**
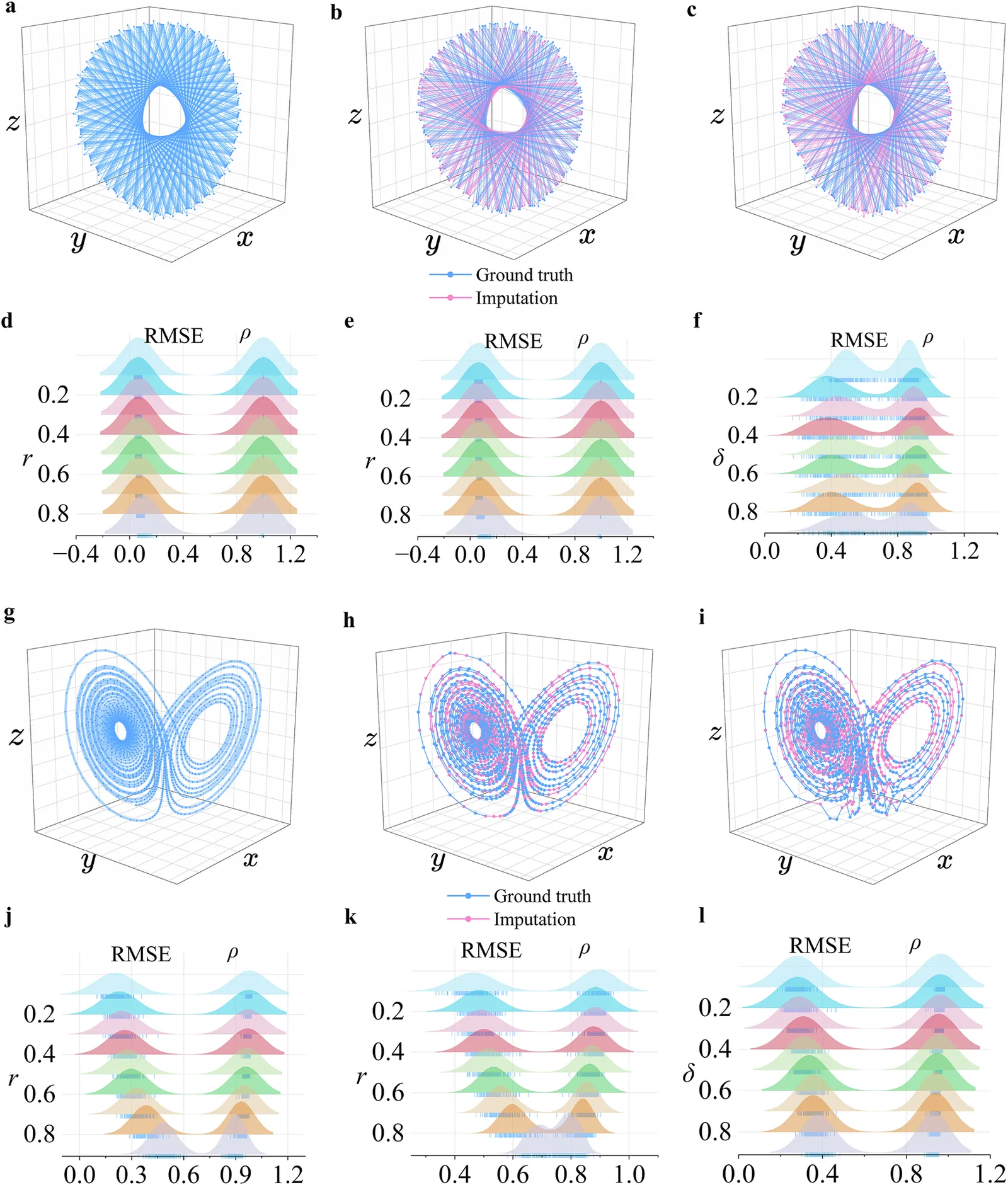
Imputation of low-dimensional model systems. **a** Original dynamical structure of the 3D ecology system. **b**, **c** Approximately recovered structure from time series: 50% of the values in variables *y* and *z* are randomly removed, respectively, with full reconstruction from variable *x* as model input. **d**, **e** Imputations for incomplete variables *y* and *z* as the proportion of missing values increases from 10% (r=0.1) to 90% (r=0.9). For each case, two performance metrics (RMSE (left panel) and ρ (right panel)) are displayed via kernel distribution derived from 200 repeated experiments; the horizontal axis represents the possible RMSE and ρ values. The blue tick marks along the horizontal axis display the distribution of raw values. **f** Imputation of variable *y* (r=50%) with increasing additive white noise magnitudes from 0.1 to 0.9. (**g**–**k**) Similar imputation cases in the 3D chaotic Lorenz system (3D), where the reconstruction from the complete variable *x* serves as the model inputs to impute substantial missing values in variables *y* and *z*, respectively. **l** Robustness test of the FPRM framework against increasing noise level, where variable *y* is incomplete (r=0.5). Note: r denotes the ratio of missing values, and δ represents the magnitude of additive white noise.

To demonstrate the ability of the proposed framework, we begin with a benchmark nonlinear ecological system comprising three interacting species: *x*, *y*, and *z*^11^ (see Eq. 3 in *Materials and “Methods”*). This system dynamically evolves within a 3D state space, forming a trajectory structure (Fig. 3a) accompanied by three distinct time-series variables. Given partial variables with substantial missing values, e.g., when 50% of the data points in the variables *y* or *z* are randomly removed, the FPRM framework efficiently imputes these missing values via mappings from the full reconstruction (derived from a complete series *x*) to partial ones (derived from incomplete series *y* and *z)*. The approximate recovered dynamics of the underlying system are presented in Fig. 3b, c. We further test imputation performance by increasing the proportion of missing elements from 10% to 90%. Nearly all the values of ρ are distributed above 0.8, whereas the normalized RMSEs remain below 0.4 (Fig. 3d, e). Even when 90% of the values are randomly removed, the FPRM framework achieves a high performance, with ρ=0.99, RMSE=0.11 for the variable *y*, and ρ=0.99,RMSE=0.13 for *z* on average. In addition, the FPRM framework is verified as robust against increasing noise levels (Fig. 3f). Extensive experiments show that when the variable *x* has missing values, the imputations maintain a high performance (Supplementary Fig. S3).

To test the performance of our framework in chaotic systems^47,48^, we select the classic Lorenz system (Eq. 4 in *Materials and “Methods”*) as a benchmark. The FPRM framework achieves reliable imputations when the variable *y* or *z* contains substantial missing values (Fig. 3g–k); the imputation results for variable *x* are provided in Supplementary Fig. S4. The framework maintains a high performance even with 90% missing values, yielding average ρ=0.9 and RMSE=0.48 for *y* and ρ=0.81 and RMSE=0.68 for *z*, respectively. Tests under deteriorating noise conditions confirm the robustness of the FPRM framework (Fig. 3l). We additionally evaluate the method’s robustness against variations in dataset length (Supplementary Fig. S5) and colored noise (Supplementary Fig. S6). Moreover, we apply the framework to the classic Chen system^49,50^ (Supplementary Fig. S7), which exhibits distinct chaotic behavior compared to the Lorenz system.

### Validation in high-dimensional model systems

**Fig. 4:**
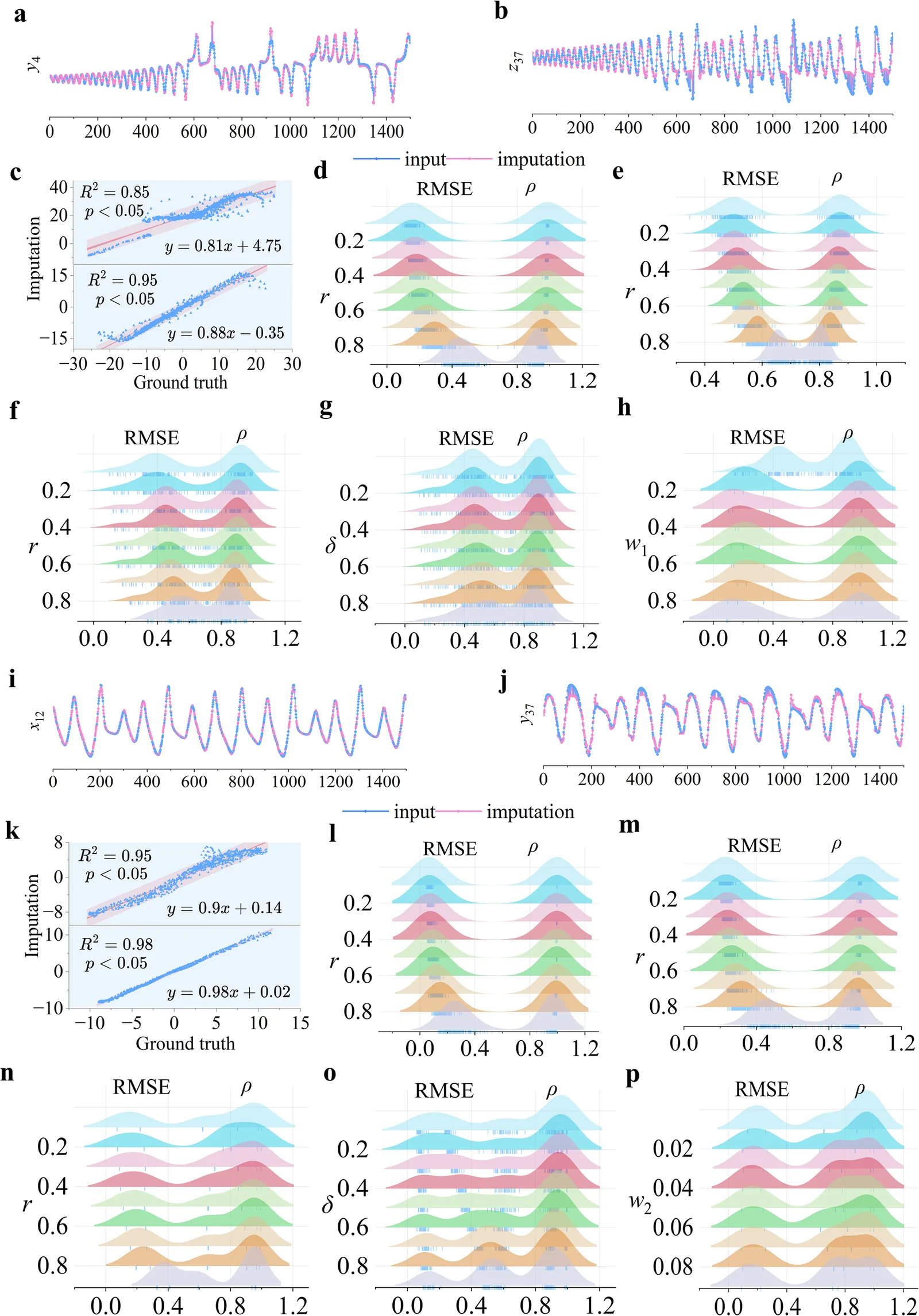
Performance of high-dimensional model systems. **a**–**c** Imputations for the 120D coupled Lorenz system, where randomly selected variables ${y}_{4}$ and ${z}_{37}$ are shown with 50% missing values and the full reconstruction of the variable ${x}_{1}$ serves as model input. The upper panel in (**c**) displays the linear correlations between the imputed values and ground truth for ${y}_{4}$, and the lower panel corresponds to those for ${z}_{37}$. **d**, **e** Imputations for variables ${y}_{4}$ and ${z}_{37}$ under increasing missing proportions (r=0.1 to r=0.9). Performance metrics (RMSE and ρ) are visualized via kernel distribution from 200 repeated experiments. **f** Imputations when all variables except ${x}_{1}$ contain increasing missing elements. **g** Robustness test of additive white noise with magnitudes from δ=0.1 to δ=0.9. **h** Robustness test for varying coupling magnitudes ${w}_{1}$. **i**–**p** Similar cases for the imputations in the 120D coupled Rössler system, FPRM also demonstrates efficient imputation performance. Note: ${w}_{1}$ and ${w}_{2}$ represent the coupling magnitudes in the coupled Lorenz system and the coupled Rössler system, respectively.

To demonstrate the FPRM framework in high-dimensional systems, we first consider a 120D coupled Lorenz system (see Eq. 4 in *Materials and “Methods”*). When the majority of variables are incomplete, specifically when only a single variable remains complete, reconstructing the missing values for all other variables poses a pronounced challenge for traditional regression approaches. This challenge intensifies significantly with extensive data loss in incomplete variables. Suppose that a variable ${x}_{1}(t)$ contains complete time series information (other cases are treated in *Discussions*); the FPRM framework performs imputation by training multiple mappings, from full state reconstruction based on ${x}_{1}(t)$ to every partial reconstruction derived from an incomplete series. For instance, the framework enables reliable imputation of incomplete variables, ${y}_{4}$, and ${z}_{37}$ (Fig. 4a–e). Furthermore, we present the imputation results for all the other 119 incomplete variables (Fig. 4f). Although the average imputation accuracy decays monotonically with increasing missing value proportions, FPRM remains reliable even when these incomplete variables contain 80% and 90% missing values with average ρ=0.87 and RMSE=0.48, ρ=0.84,and RMSE=0.54, respectively (Fig. 4f). Under deteriorating noise conditions and coupling magnitude variation, FPRM is confirmed to be robust and efficient (Fig. 4g, h).

Next, we validate our framework on another widely used benchmark system: the 120D coupled Rössler system (see Eq. 5 in *Materials and “Methods”*). We investigate a scenario in which the majority of variables exhibit substantial missingness. Specifically, only a single variable (e.g., ${x}_{1}(t)$) remains fully observed, with all other variables being incomplete (other cases are treated in *Discussions*). The results demonstrate the reliability of FPRM across varying proportions of missing elements (Fig. 4i–m). Notably, even when incomplete variables exhibit a 90% value loss, the FPRM achieves reliable imputation, yielding an average Pearson correlation coefficient ρ=0.81 and normalized RMSE=0.51 (Fig. 4n). Furthermore, our framework is validated to maintain robustness under deteriorating noise conditions and varying coupling magnitudes (Fig. 4o, p).

### Application to real-world systems

Time-series-based scientific discovery has emerged as a critical paradigm for understanding and predicting various real-world complex dynamical systems, where the underlying governing rules are often intractable and only partial time-series data are typically accessible. Incomplete data can exacerbate information gaps and introduce uncertainties, thereby negatively affecting the reliability and efficiency of data-driven analyses. To demonstrate the efficacy of the FPRM framework in real-world systems, we leverage cross-disciplinary datasets spanning the transportation system, neuroscience, finance, and climate (the climate results are detailed in Supplementary Figs. S8, S9).

**Fig. 5:**
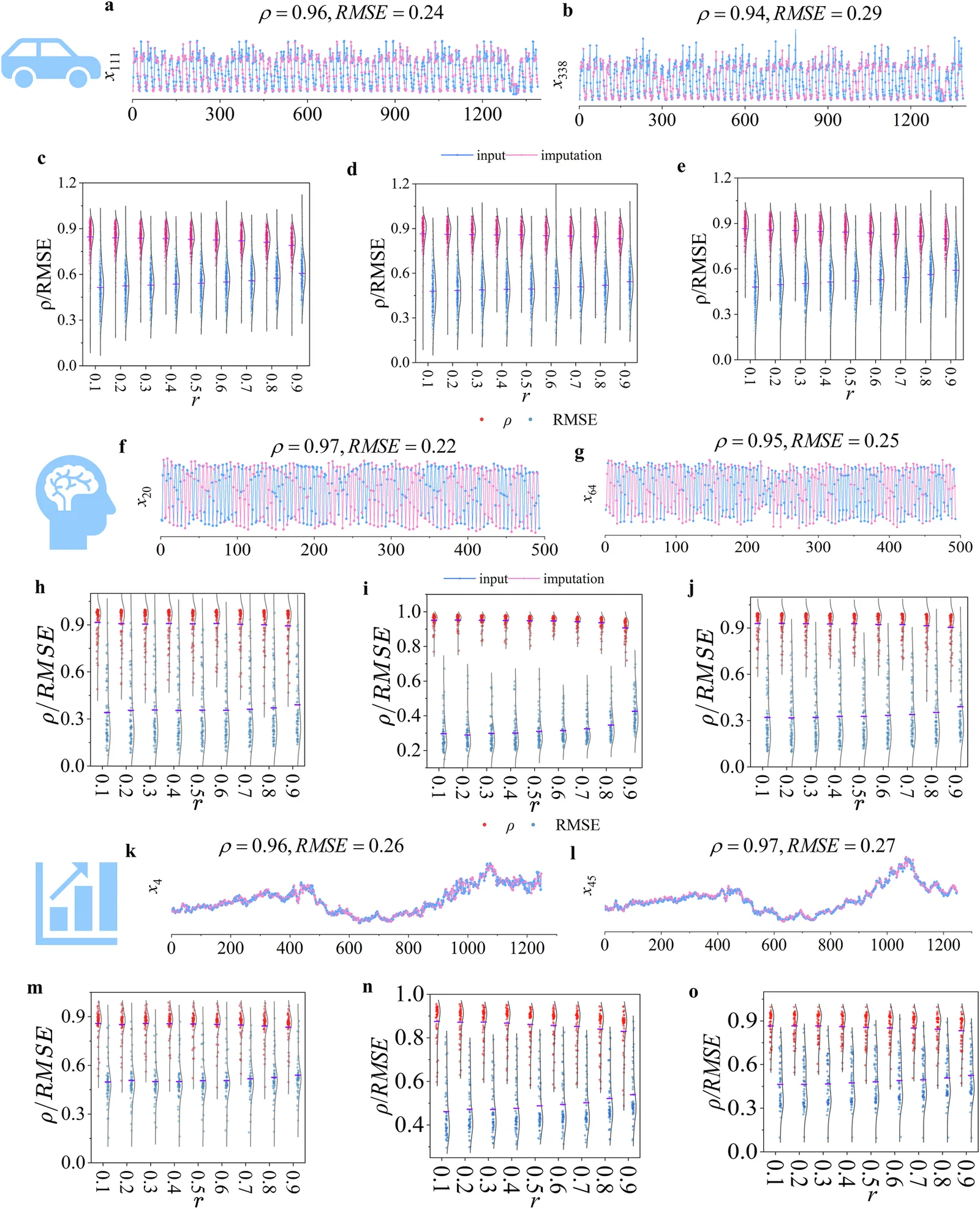
Imputation results for real-world systems (transportation, neuroscience, and finance). **a**, **b** Imputations for randomly selected road occupancy rates ${x}_{111}$ and ${x}_{338}$ in the transportation system (r=0.5), where ${x}_{1}$ is assumed complete series and used to construct the model input. **c**–**e** Imputations for all incomplete series when only one variable is fully observed (e.g., ${x}_{11}$, ${x}_{31}$, or ${x}_{52}$). The performance metrics for 499 incomplete observations with increasing missingness proportions from r=0.1 to r=0.9 are presented as red (ρ) and blue (RMSE) plots. **f**, **g** Imputations for randomly selected EEG series ${x}_{20}$ and ${x}_{64}$ with the full reconstruction from ${x}_{1}$ used as the model input (r=0.5). **h**–**j** Imputations of the remaining 64 series when only one variable contains complete state information (e.g., ${x}_{21}$,${x}_{35}$, or ${x}_{64}$). **k**,** l** Performance of exchange rate series for ${x}_{4}$ and ${x}_{45}$ with the full reconstruction from ${x}_{1}$ utilized as the model input (r=0.5). **m**–**o** Imputations of the remaining 51 exchange rates when one variable remains complete (e.g., ${x}_{6}$, ${x}_{12}$ or ${x}_{51}$).
ii) In neuroscience, time-series data (e.g., electroencephalograms (EEG) and Functional Magnetic Resonance Imaging (fMRI)) constitute one of the most critical cornerstones for investigating and elucidating the structure and function of the brain^52^. The accurate imputation of incomplete data is vital to ensure the validity and reliability of subsequent analyses and decision-making in diagnoses. Here, we apply the FPRM to 64 EEG signal series (denoted as ${x}_{1},{x}_{2},\ldots,{x}_{64}$) from a healthy participant (more details are described in ref. ^53^, with each series comprising 800 data points. All the datasets are preprocessed via the Z-score normalization. Suppose that two randomly selected variables (e.g., ${x}_{20}$ and ${x}_{64}$) contain 50% missing states; the FRRM framework enables reliable imputations (Fig. 5f, g). Even when only a single variable (e.g., ${x}_{21}$, ${x}_{35}$, or ${x}_{64}$) is fully observed and all others exhibit an increasing proportion of missingness (from 10% to 90%), the framework enables efficient imputation across nearly all variables (Fig. 5h–j). In the 90% missingness scenario, the average performances for the three cases are ρ=0.89 and RMSE=0.39,ρ=0.91 and RMSE=0.42, ρ=0.9 and RMSE=0.38, respectively.
iii) In finance, financial time-series data (e.g., asset prices) are widely used to support portfolio investment management (e.g., asset allocation optimization) and risk management (e.g., credit risk early warning), whereas incomplete data can distort these analyses and impede the formulation of reliable decisions^54^. To demonstrate the efficacy of our framework, we select the daily closing prices of 52 exchange rate series (${x}_{1},{x}_{2},\ldots,{x}_{52}$) against the US dollar. Each series contains 1256 data points, and all variables are normalized using the Z-score method. When two randomly selected variables, ${x}_{4}$ and ${x}_{45}$, contain 50% missing values, the FPRM framework achieves efficient imputation for both (Fig. 5k, l). Even when only one variable remains complete (e.g., ${x}_{6}$, ${x}_{12}$, or ${x}_{51}$) and all others randomly lose 90% of their values, the framework remains reliable, with the average ρ=0.83 and RMSE=0.54, ρ=0.82 and RMSE=0.55, ρ=0.83 and RMSE=0.52 (Fig. 5m–o).

i) For the transportation system, we focus on freeway traffic monitoring in the San Francisco Bay Area^51^. We select road occupancy rates (ranging from 0 to 1) measured by loop detectors on major freeways. The raw dataset is publicly available from the California Department of Transportation at http://pems.dot.ca.gov. Collected over the period from 2015 to 2016, this dataset contains hourly recordings spanning 48 months. This dataset yields 500 time series (denoted as ${x}_{1},{x}_{2},\ldots,{x}_{500}$). When multiple observations contain missing values (e.g., ${x}_{111}$ and ${x}_{338}$), FPRM enables reliable imputations even when 50% of records are lost (Fig. 5a, b). Moreover, we investigate a challenging scenario in which only one variable is fully observed, while all other variables exhibit substantial missingness. For instance, when ${x}_{11}$, ${x}_{31}$ or ${x}_{52}$ remain complete, the framework recovers nearly all incomplete observations across increasing proportions of missing values (Fig. 5c–e). Even when each incomplete observation contains 90% missing values, FPRM maintains reliability, yielding average ρ=0.79RMSE=0.58, ρ=0.84 andRMSE=0.54, ρ=0.8 and RMSE=0.59, respectively.

## Discussions

### Comparison

We compare our framework with several classic approaches, including mean imputation, *k*-nearest neighbors (*k*-NN)^31^, Gaussian process regression (GPR)^35^, support vector machine (SVM)^34^, random forest (RF)^33^, and a two-layer neural network (NN)^31^. These machine learning approaches accomplish imputation by directly learning the interdependencies between time series. Despite these static imputation methods, we further consider three representative classical dynamics-aware methods: MVCM^25^, S-map^11^ and MetaTSI^43^. As time‑series reconstruction‑based and dynamics‑aware paradigms, they are purely data‑driven and require no prior knowledge of the system’s governing equations. The results show that the FPRM clearly outperforms traditional methods across multiple datasets (see Table 1).

The FPRM framework uses Gaussian process regression (GPR) to train mappings between full and partial reconstructions for imputation. Theoretically, alternative learners can also build such mappings. We replace GPR with SVM and NN to validate the FPRM paradigm, both delivering competitive results (see SVM* and NN* in Table 1). Nonetheless, the FPRM integrated with GPR attains superior overall performance. Compared with traditional machine learning methods, approaches that incorporate prior system dynamics exhibit greater potential for accurate imputation. This is further supported by the relatively inferior imputation performance of standalone GPR, SVM, and NN algorithms.

### Overall performance

**Fig. 6:**
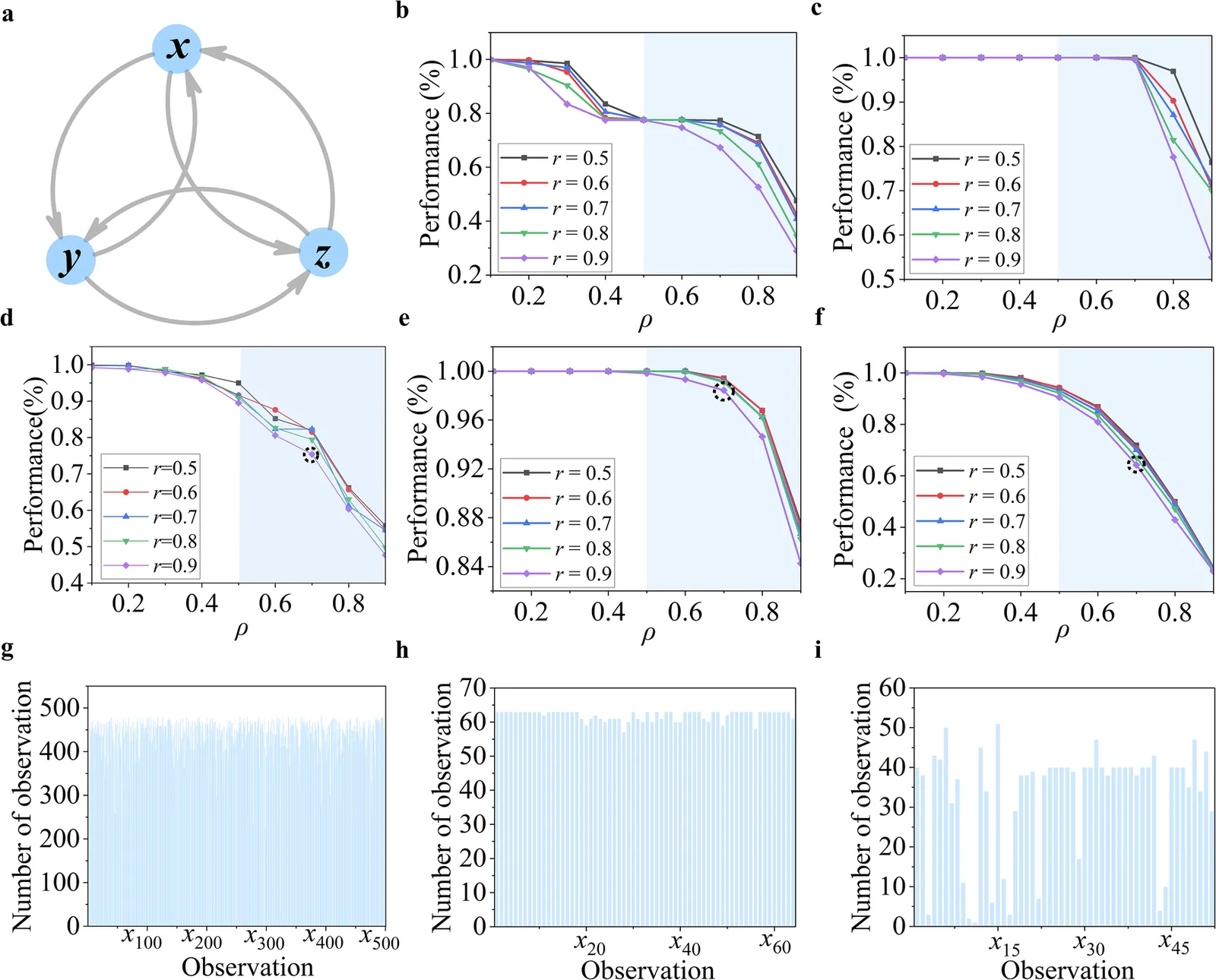
Overall imputation performance of the FPRM framework when only one variable is fully observed in complex systems. **a** Direct network from the 3D ecology system, constructed based on pairwise imputation performance ($\rho \ge 0.9$), with 90% missing values for each incomplete variable. **b** Overall imputation performance of the 120D Lorenz system (measured by the ratio of actual connections in the underlying network) as missingness increases from r=0.5 to r=0.9. **c**–**f** Corresponding results for the 120D Rössler system, 500D road occupancy rates, 64D EEG signals, and 52D exchange rates series. **g** Imputation capacity (the number of available imputed variables) for each variable in the transportation system. Each bar represents the number of available imputed variables for a given complete variable, where all other variables contain 90% missingness and the imputation performance satisfies $\rho \ge 0.7$ (corresponding to the case denoted by the dashed coil in (**d**)). **h**, **i** Similar analysis of imputation capacity in neuroscience and finance (corresponding to cases denoted by the dashed coils in (**e**,** f**)).

We demonstrated the imputation performance under the typical assumption that a specific variable is fully observed (e.g., the first variable), whereas all other variables within the system remain incomplete. However, in practical, real-world complex systems, missing values may randomly occur in any system component rather than being fixed to certain variables. H Here, we further discuss the overall performance across all feasible application scenarios, where each variable is alternately assumed to be fully observed in turn and adopted to impute all other incomplete variables within the dynamical system. Across all variable pairs, a directed, unweighted network can be easily constructed, where each node represents an individual system variable, and a directed edge from node *i* to *j* is inferred based on the imputation performance, specifically when the corresponding ρ values exceed a particular threshold value. For example, a fully interconnected directed network is generated from the 3D ecology system (Fig. 6a). The ratio of existing edges to the maximum number of possible edges (ranging from 0 to 1) serves as a simple yet effective metric for quantitatively evaluating the overall performance of our imputation framework^55^. A ratio closer to 1 indicates a highly interconnected network, which reflects the stronger generalized imputation capacity of the framework, whereas a ratio near 0 denotes a sparse network with very few effective, corresponding to a relatively lower imputation capacity. The results show that the overall performance of our framework decreases as the missing ratio increases from 50% to 90%. Notably, our framework remains effective; for both the model and real-world systems, the ratio of actual connections in the underlying reconstructed network exceeds 60%, even when each incomplete variable contains up to 90% missing values (Fig. 6b–f). Although most variables in a complex dynamical system can individually impute multiple incomplete variables with satisfactory accuracy, few can only effectively impute merely specific subsets of system variables (Fig. 6g–i). This noticeable discrepancy may stem from the inevitable observational noise and inherent nonlinear complexity of real-world dynamical systems.

### Potential for handling non-random missingness

In this study, missing values are typically simulated by randomly removing entries from the original time series, with missing data often distributed discretely in the series. In practical scenarios, however, missingness may follow a non-random pattern. Typical manifestations include continuous missing segments, threshold-induced missingness, and periodic missingness. Continuous missing segments correspond to prolonged consecutive data losses over a certain time interval, which widely occurs in sensor outage and device failure^56^. Threshold-induced missingness arises when observations are missing once the underlying signal exceeds or falls below a critical threshold value. Periodic missingness refers to missing values recurring at regular intervals, which is prevalent in cyclic monitoring tasks.

We first investigate continuous missing segments under three typical scenarios: missing entries located in the first, middle, and latter halves of the time series. Experimental results across multiple datasets validate the capability of our framework to accurately impute consecutive missing elements (Supplementary Fig. S10). For threshold-induced missingness, we design quantile-based missing experiments to mimic realistic non-random patterns. Missing values are generated when the original data falls below fixed quantile thresholds of 5, 10, 15, and 20% of the overall distribution. Our method is capable of reliable imputation for multiple variables in such scenarios (Supplementary Figs. S11, S12). Meanwhile, its performance can be further improved in more complex situations, such as threshold-induced missing data featuring abundant abrupt data points. Finally, for periodic missingness, we perform quantitative experiments with periodic intervals ranging from 2 to 11 sampling steps. The results confirm that our framework can effectively recover periodic missing entries for complex dynamical systems (Supplementary Figs. S13, S14).

### Merit and limitation

This study proposes a data-driven imputation framework derived from the efficient integration of time-series delay embedding and Gaussian process regression. Its reliability has been validated through comparisons with multiple benchmark models and real-world datasets, and it has several distinct advantages.

1. The architecture of the FPRM framework adheres rigorously to theoretical principles, with its core structure derived directly from the canonical embedding theorem in dynamical systems theory. In a dynamical system, the interacting components evolve over time; collectively, they form an attractor in the state space together with the corresponding coordinate time series of each component. According to Takens’ embedding theorem, the structure of the attractor can theoretically be reconstructed from any single-component time series^44^. Moreover, the reconstructed structures derived from the distinct output time series preserve the fundamental dynamics of the underlying system, although expressed via different coordinate measurements^45^. Thus, the existence of inherent equivalence (i.e., diffeomorphism) among all the reconstructed attractors. These reconstructed attractors admit one-to-one mappings, forming a critical theoretical foundation of the FPRM framework. On the other hand, Takens’ theorem underscores the feasibility of imputing missing values in time series from dynamical systems. Generally, if missing values were purely random and unpredictable numbers with no dynamical regularity, they could not be meaningfully imputed. Importantly, many real-world systems can be approximated as dynamical systems, even when their explicit governing equations remain unknown. Takens’ theorem guarantees that the missing values in a dynamical system are not arbitrary random values, as constrained by the requirement that time-series-based reconstruction must preserve the fundamental dynamics of the underlying system. Random values would lead to reconstructed attractors with drastically altered topological structures and entirely different dynamics, which directly contradicts the premises of the theorem. Consequently, missing values (except for some abrupt and unpredictable transitions such as tipping points^56^) cannot be regarded as purely random values; instead, they are governed by specific constraints that ensure that the imputed time series yields a reconstructed attractor that is diffeomorphic to the true attractor of the underlying system. This theoretical basis validates the feasibility of missing-value imputation, as well as underpins the interpretability of our FPRM framework.
2. Recovering missing dynamical states in high-dimensional systems remains challenging due to complex interactions among multiple variables and widespread incomplete observations. FPRM reconstructs a topologically diffeomorphic attractor of the original system via delay embedding under proper embedding settings. High-dimensional systems generally possess redundant observational information, and their intrinsic dynamical evolution can be well characterized within a properly embedded state space^19^. The embedding dimension is determined by the inherent dynamical properties of the system rather than the number of observed variables, which is often lower than that of the original dissipative high-dimensional system^57^. Imputation is thereafter implemented within this dynamically consistent embedded space, which naturally alleviates the curse of dimensionality and lowers computational overhead, particularly for kernel function evaluation in Gaussian Process Regression (GPR). To impute multiple incomplete components, FPRM learns multiple mappings between reconstructed attractors derived from complete and incomplete time series. Here, a full reconstruction from the complete series serves as a reliable input, and GPR acts as a useful learning model that enables the imputation of multiple incomplete variables. This framework offers more efficient techniques than previous ones, many of which struggle with model or input feature selection and require different inputs or models for imputing distinct targets within the same system^31^.
3. Traditional regression-based approaches perform imputation by directly fitting the statistical relationships between complete and incomplete time series variables^33–35^. They often suffer from feature scarcity (e.g., only one fully observed variable serves as the model input) and unstable statistical correlations, with the estimated correlations typically exhibiting temporal variability. Furthermore, when an incomplete series contains extensive missing values, both traditional regression models and some advanced ML/AI approaches are severely constrained, possibly because of insufficient training samples. By contrast, FPRM learns the topologically diffeomorphic relationships between the reconstructed attractors from both complete and incomplete series. In contrast to traditional approaches that consider only a single time series as a predictor, when only one variable is fully observed, FPRM utilizes its corresponding *E*-dimensional (E>1) full reconstruction as the model input. This design not only enriches the input information and efficiently alleviates feature scarcity but also contains prior dynamics of underlying systems. Moreover, the integration of Gaussian process regression (GPR) enables a non-parametric framework (requiring no explicit regression equations or parameters, thereby avoiding interference from unstable inter-series correlations), as well as leverages GPR’s powerful capability for handling small samples, a critical advantage for imputing extensive missing values, where only limited training information is available (e.g., when 90% of the values are missing, only 10% of the values are available for training).

In theory, FPRM enables the imputation of all incomplete variables using any complete variable from the underlying system (e.g., the 3D ecology system (Fig. 6a). The underlying system can be approximated as a deterministic dynamical system satisfying Takens embedding theorem, so that reconstruction from each observed time series can produce diffeomorphic attractor manifolds. While the average overall imputation performance is promising for real-world datasets, where most variables in a complex system can individually impute all other incomplete variables, some are only capable of imputing specific subsets (Fig. 6g–i). This discrepancy may arise from the inevitable noise and inherent complexity of real-world systems. Another limitation is that the FPRM currently requires training a dedicated mapping for each incomplete variable, rather than enabling the simultaneous imputation of all incomplete components. In addition, real-world systems shaped by multifaceted influences may experience abrupt behavioral transitions that manifest as tipping points in their output time series, and imputing such tipping points remains a non-trivial challenge^58,59^. A further aspect is that the framework relies on full reconstructions from complete variables as valid inputs, which restricts its applicability to scenarios where no complete variables are available (i.e., all system components contain substantial missing values). Nevertheless, our framework can be potentially optimized to accommodate certain cases. Specifically, for scenarios where all variables suffer from simultaneous missing entries at identical time points, our framework can be extended to a lag FPRM variant. This variant employs a segment-wise cross-reconstruction strategy for synchronous missing data. It builds dynamical mappings between attractors reconstructed from staggered valid and incomplete segments of paired time series, enabling the recovery of synchronous missing entries without fully observed variables (Supplementary Fig. S15). Results on multiple datasets validate the capability of our framework in handling scenarios with simultaneous missing entries across all variables (Supplementary Figs. S16, S17). Nevertheless, there remains room for further improvement handling extensive long continuous, synchronous missing segments across all variables, as gradually accumulated dynamic prediction deviations may impair the overall imputation performance.

In summary, the FPRM framework provides a viable approach for repairing data gaps, facilitating improved data-driven modeling and analysis in practical scientific applications. Moreover, as an effective integration of traditional mathematical theorems and advanced machine learning algorithms—with the former laying theoretical underpinnings and architectural foundations, and the latter facilitating operational implementation—this synergistic framework highlights the potential of “mathematics + ML/AI” paradigms in scientific inquiry.

## Methods

### Synthetic data from models

First, we detail the benchmark model systems employed in our approach, including the nonlinear ecology, coupled Lorenz, and coupled Rössler systems.

A nonlinear ecological system comprises three interacting species, as expressed in Eq. 3. Starting from the initial state $(x(0),y(0),z(0)=\left(\right.0.4,0.5,0.6)$, we generate a population time series of length 500. To eliminate the transient states, the first 100 data points are discarded:

3 $$\begin{array}{c}x(t+1)=x(t)[4-4x(t)-2y(t)-0.4z(t)],\\ y(t+1)=y(t)[3.1-0.31x(t)-3.1y(t)-0.93z(t)],\\ z(t+1)=z(t)[2.12+0.636x(t)+0.636y(t)-2.12z(t)].\end{array}$$

The coupled Lorenz system is formulated using Eq. 4, where i=1,…,n represents the *i*th coupled subsystem, and ${w}_{1}$ measures the coupling strength between connected subsystems. It describes the classic 3D Lorenz system when i=1. In addition, we define a 120D coupled system as i=40. Within the time interval [10 40], for each variable, we generate state points by selecting an initial state starting from 0.3 with a sequential increment of 0.03. The first 100 data points are removed to eliminate transient states:

4 $$\begin{array}{c}{\dot{x}}_{i}={a}_{1}({y}_{i}-{x}_{i})+{\omega }_{1}{\sum }_{j=1,j\ne i}^{N}({x}_{j}-{x}_{i}),\\ {\dot{y}}_{i}={x}_{i}({b}_{1}-{z}_{i})-{y}_{i},\\ {\dot{z}}_{i}={x}_{i}{y}_{i}-{c}_{1}{z}_{i}.\end{array}$$

where $${a}_{1}=10$$,$${b}_{1}=28$$, $${c}_{1}=8/3$$ and $${w}_{1}=0.1$$.

The coupled Rössler system is expressed by Eq. 5, where i=1,…,n represents the *i*th coupled subsystem, and ${w}_{2}$ measures the coupling strength between connected subsystems. It forms a 120D coupled system when i=40. From a time interval [0 100], 1717 state points are generated for each variable by selecting an initial state starting from 0.1 and incrementing it sequentially by 0.03. The first 100 data points are removed to eliminate transient states:

5 $$\begin{array}{c}{\dot{x}}_{i}=-{y}_{i}-{z}_{i}+{\omega }_{2}{\sum }_{j=1,j\ne i}^{N}({x}_{j}-{x}_{i}),\\ {\dot{y}}_{i}={x}_{i}+{a}_{2}{y}_{i},\\ {\dot{z}}_{i}={b}_{2}+{z}_{i}({x}_{i}-{c}_{2}).\end{array}$$

where $${a}_{2}={b}_{2}=0.2$$,$${c}_{2}=5.7$$ and $${w}_{2}=0.1$$.

### FPRM framework

This framework is developed based on the classic dynamical system theory, particularly the time-series embedding theory, and integrates a machine learning algorithm. In an *n*-dimensional dynamical system, suppose that the observation ${x}_{i}(t)$($i\in \{1,2,\ldots,n\}$,t=1,2…,l, *l* is the length of the time series) is a complete series, while ${x}_{j}(t)$($j\ne i,j\in \{1,2,\ldots,n\}$) is an incomplete series. The proposed FPRM framework operates as follows.

1) Full reconstruction from a complete series. According to Takens’ delay embedding theory, with an appropriate embedding dimension *E* and time lag τ, a single series ${x}_{i}(t)$ can be efficiently used to reconstruct an attractor $M({x}_{i})$. Each state point on $M({x}_{i})$ can be expressed as an ${X}_{i}(t)=({x}_{i}(t),{x}_{i}(t+\tau ),\ldots,{x}_{i}(t+(E-1)\tau ))$. This reconstructed attractor contains the full information because all entries in $M({x}_{i})$ are derived from the observed complete series:

6 $$M({x}_{i})=\left[\begin{array}{cccc}{x}_{i}(1) & {x}_{i}(1+\tau ) & \cdots & {x}_{i}(1+(E-1)\tau )\\ {x}_{i}(2) & {x}_{i}(2+\tau ) & \cdots & {x}_{i}(2+(E-1)\tau )\\ \vdots & \vdots & \ddots & \vdots \\ {x}_{i}(m) & {x}_{i}(m+\tau ) & \cdots & {x}_{i}(l)\end{array}\right],$$

where m=l−(E−1)τ.

Takens’ theory states that the reconstructed attractor has a topologically isomorphic structure to that of the original attractor (shaped by all the interacting components). Thus, a one-to-one mapping f between the original attractor *M* and the reconstructed attractor $M({x}_{i})$ exists. Mathematically, $\forall \hat{X}(t)\in M$,$\exists {X}_{i}(t)\in M({x}_{i})$, we have

7 $${X}_{i}(t)=f(\hat{X}(t)).$$

As f is a one-to-one mapping, we can deduce an inverse mapping ${f}^{-1}$ from the state points of the reconstructed attractor $M({x}_{i})$ to those of the original attractor *M*.

8 $$\hat{X}(t)={f}^{-1}({X}_{i}(t)).$$

2) Partial reconstruction from an incomplete series. From the incomplete time-series variable ${x}_{j}(t)$, it is possible to reconstruct an attractor $M({x}_{j})$ using an appropriate embedding dimension *E* and time delay τ, where each state of $M({x}_{j})$ is represented as ${X}_{j}(t)=({x}_{j}(t),{x}_{j}(t+\tau ),\ldots,{x}_{j}(t+(E-1)\tau ))$. This reconstructed attractor contains only partial information because certain entries are missing owing to the transformation from an incomplete time series.

9 $$M({x}_{j})=\left[\begin{array}{cccc}{x}_{j}(1) & {\underline{{x}_{j}(1+\tau )}} & \cdots & {\underline{{x}_{j}(1+(E-1)\tau )}}\\ \vdots & \vdots & \cdots & \vdots \\ {\underline{{x}_{j}(k)}} & {x}_{j}(k+\tau ) & \cdots & {\underline{{x}_{j}(k+(E-1)\tau )}}\\ \begin{array}{c}\vdots \\ {x}_{j}(m)\end{array} & \begin{array}{c}\vdots \\ {\underline{{x}_{j}(m+\tau )}}\end{array} & \begin{array}{c}\ddots \\ \cdots \end{array} & \begin{array}{c}\vdots \\ {x}_{j}(l)\end{array}\end{array}\right],$$

where 1<k<m and underlined elements represent the missing values.

According to embedding theory, this partial reconstruction should have a topologically isomorphic structure to the original attractor. A one-to-one mapping between the original attractor *M* and the reconstructed attractor $M({x}_{j})$ is theoretically valid. Mathematically, $\forall \hat{X}(t)\in M$,$\exists {X}_{j}(t)\in M({x}_{j})$, we obtain

10 $${X}_{j}\left(t\right)=g\left(\hat{X}\left(t\right)\right),\,\hat{X}\left(t\right)={g}^{-1}\left({X}_{j}\left(t\right)\right).$$

Therefore, owing to the underlying diffeomorphism between the partial reconstruction and the original system, the missing states are not randomly distributed, but are constrained by a deterministic rule that is closely associated with the original dynamical system. This guarantees the feasibility of recovering missing dynamics.

3) Imputation by mapping from full to partial reconstructions. Both the full reconstruction $M({x}_{i})$ and partial reconstruction $M({x}_{j})$ are diffeomorphic to the original attractor *M*; thus, the diffeomorphism property holds between the full and partial reconstructions.

11 $$F:M({x}_{i})\to M({x}_{j}).$$

Thus, $\forall {X}_{j}(t)\in M({x}_{j})$, $\exists {X}_{i}(t)\in M({x}_{i})$, we infer a smooth mapping ${F}_{i}$:

12 $${X}_{j}(t)={F}_{i}({X}_{i}(t)).$$

where *F* is inferred as $F(x)=g({f}^{-1}(x))$ according to Eq. 8 and 10.

Note that the first column of the partial reconstruction is directly sequenced from the observed series (i.e., ${x}_{j}(t)$, t=1,2,…,m, and m=l−(E−1)τ), which contains most of the missing elements of the original series. Therefore, we deduce a mapping from $M({x}_{i})$ to the components in the first column of $M({x}_{j})$.

13 $$F\left[\begin{array}{cccc}{x}_{i}(1) & {x}_{i}(1+\tau ) & \cdots & {x}_{i}(1+(E-1)\tau )\\ \vdots & \vdots & \cdots & \vdots \\ {x}_{i}(k) & {x}_{i}(k+\tau ) & \cdots & {x}_{i}(k+(E-1)\tau )\\ \vdots & \vdots & \ddots & \vdots \\ {x}_{i}(m) & {x}_{i}(m+\tau ) & \cdots & {x}_{i}(l)\end{array}\right]=\left[\begin{array}{c}{x}_{j}(1)\\ \vdots \\ {\underline{{x}_{j}(k)}}\\ \vdots \\ {x}_{j}(m)\end{array}\right].$$

The contemporary known values from $M({x}_{i})$ and $M({x}_{j})$ are employed to train mapping *F*, and the missing values can be recovered once *F* is identified as follows:

14 $${x}_{j}(k)=F\left(\right.{x}_{i}(k),{x}_{i}(k+\tau ),\ldots,{x}_{i}(k+(E-1)\tau ),$$

where $$k\in \{1,2,\ldots,m\}$$.

According to Takens’ theorem, each system variable yields a reliable reconstruction. Thus, in a high-dimensional system in which most variables are incomplete, the imputation task can be performed via multiple mappings, from full to partial reconstructions.

In this study, we employ the classic Gaussian process regression (GPR) to identify the mapping *F* in Eq. 11. Suppose $X\in {R}^{E}$ (e.g., $X={[{x}_{1},{x}_{2},\ldots {x}_{E}]}^{T}$) and y∈R are known elements from the full reconstruction and their contemporary elements from the partial reconstruction, respectively. We aim to impute the missing element $\tilde{y}=\tilde{f}(\tilde{X})$ for the input $\tilde{X}\in {R}^{E}$, where $\tilde{X}$ originates from the full reconstruction and is contemporary with the missing elements in the partial reconstruction. Assume that function *f* is distributed as a Gaussian process with mean function u(x) and covariance function *q*. We infer the following joint Gaussian distribution:

15 $$\left[\begin{array}{c}y\\ \tilde{f}\end{array}\right]\sim N\left(\left[\begin{array}{c}u(x)\\ u(\tilde{x})\end{array}\right],\left[\begin{array}{cc}q(x,x)+{\sigma }^{2}I & q(x,\tilde{x})\\ q(\tilde{x},x) & q(\tilde{x},\tilde{x})\end{array}\right]\right),$$

16 $$\tilde{q}(x,\tilde{x})=\exp (-\frac{1}{2{\theta }^{2}}{(x-\tilde{x})}^{2}),$$

where $\tilde{q}(x,\tilde{x})$ is the squared exponential kernel function and θ is the scale parameter determined by the mean of the standard deviations of the predictors.

Bayesian inference indicates that the posterior distribution is also a Gaussian process (GP) distribution as follows.

17 $$P(\tilde{f}(\tilde{x})|X,y)\sim N({u}_{\ast },{\sigma }_{\ast }^{2}).$$

Finally, we can obtain the predicted values via Eq. 18:

18 $${u}_{\ast }=u(\tilde{x})+q(\tilde{x},x){(q(x,x)+{\sigma }^{2}I)}^{-1}(y-u(x)).$$

We simulate the missing values in a time series by randomly eliminating a certain proportion (e.g., $r\in \{10\% ,20\%, \ldots , 90 \% \}$) of the values from the observed series. The Pearson correlation (ρ) between the observed and predicted data and the normalized root mean square error (RMSE) are employed to measure the performance:1

19 $$\rho=\frac{{Cov}\left(x,\widetilde{x}\right)}{{\sigma }_{x}{\sigma }_{\widetilde{x}}},{{{\rm{and}}}}\,{RMSE}=\sqrt{\frac{\frac{1}{n}\sum {\left(x-\widetilde{x}\right)}^{2}}{{\sigma }_{\widetilde{x}}}}$$

where x and $\tilde{x}$ represent the ground truth and imputed values, respectively; σ represents the standard deviation.

## Supplementary information

Supplementary Information

Transparent Peer Review file

## Data Availability

The data generated in this study have been deposited in the Figshare database under accession code https://doi.org/10.6084/m9.figshare.30446765. The raw data of road occupancy rates is in http://pems.dot.ca.gov. The EEG signal series are downloaded from http://bnci-horizon-2020.eu/database/data-sets. The exchange rate series are downloaded from https://fx.sauder.ubc.ca/fxdata.php. The wind-speed datasets are downloaded from https://cds.climate.copernicus.eu/.

## Code availability

The code for the Full-Partial Reconstruction Mapping (FPRM) method is implemented in MATLAB R2024b. This code is freely accessible at the following repository: https://doi.org/10.6084/m9.figshare.30446765.
