# Supplementary material for: Dynamics-informed machine learning for recovering extensive missing systems dynamics
Source: Nat Commun. 2026 Sep 19;17:10986. doi: 10.1038/s41467-026-77922-1 (PMC13683343; doi:10.1038/s41467-026-77922-1)
Supplement: Supplementary file 1 [file 41467_2026_77922_MOESM1_ESM.pdf]

Supplementary information for *Dynamics-Informed Machine Learning for Recovering Extensive Missing Systems Dynamics*

Content

Supplementary figures 2
1 Dynamics reconstruction in the 3D ecology system 2
2 Time series-based reconstruction in the Lorenz and Chen system 3
3 Imputation in the 3D ecology system 3
4 Imputation in the 3D Lorenz system 4
5 Robustness test for length of data 5
6 Robustness test for colored noise 5
7 Imputation in the 3D Chen system 6
8 Imputation of real-world data from climate system 7
9 Imputation for continuous missingness 9
10 Imputation for threshold-induced missingness 10
11 Imputation for periodic missingness 12
12 Imputation framework for simultaneous missingness of all variables 14
13 Imputation under different embedding dimension 16
Supplementary tables 19
1 Embedding dimension and time lag for delay embedding 19
2 Correlation dimension for datasets 19
Supplementary notes 20
1 Main imputation pipeline of the S-map algorithm 20
2 Main imputation pipeline of the MVCM algorithm 20
Supplementary references 21

Supplementary figures

1 Dynamics reconstruction in the 3D ecology system

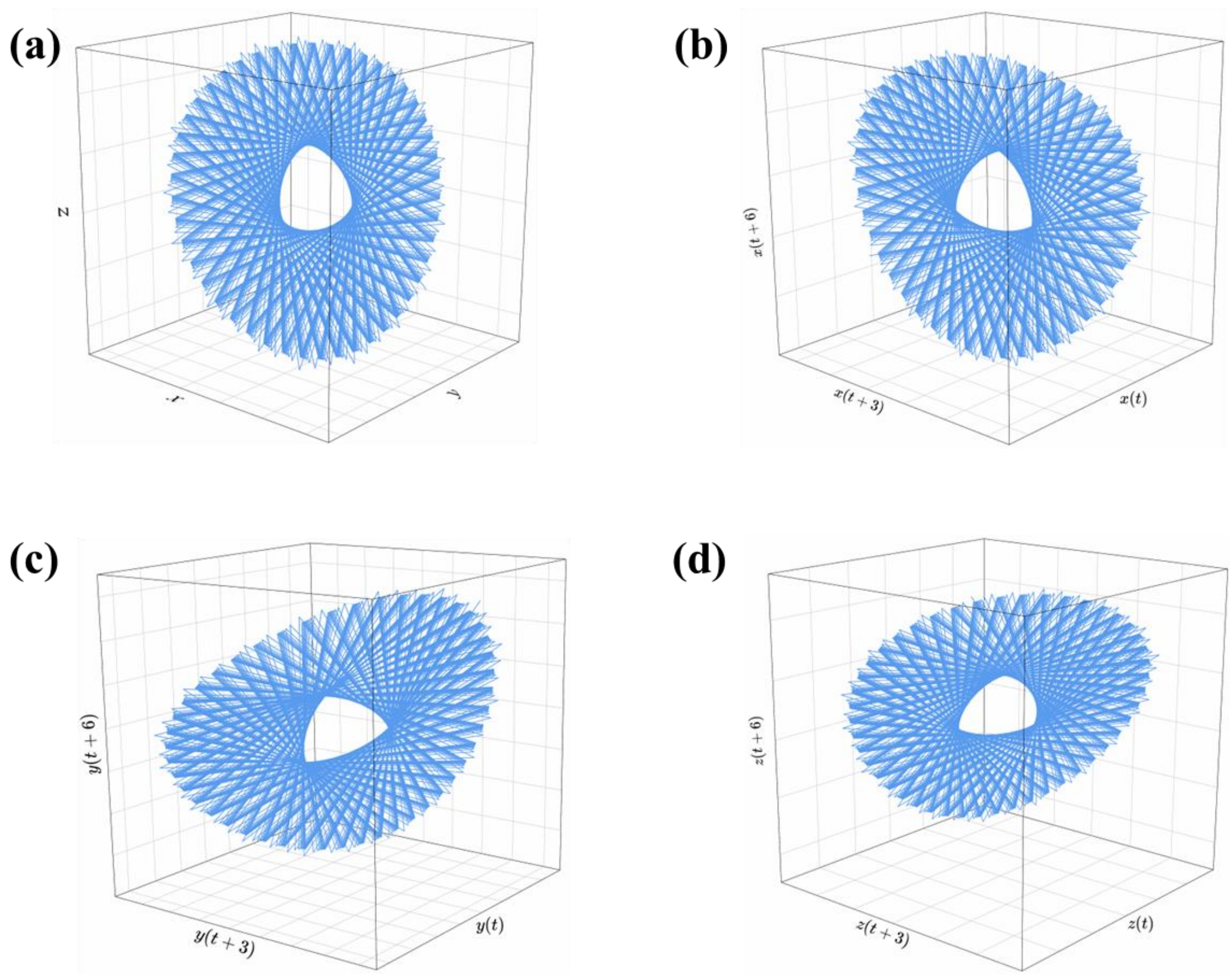

Figure S1. Reconstruction from time series in a 3-dimensional ecology system (specie variable x, y and z). (a) Original dynamical structure in state space. (b-d) Reconstructed dynamical structures from output time series x, y, and z, respectively ($E = 3, \tau = 3$). The dynamical structure of the ecological system can be well reconstructed from its output time series. According to Takens' embedding theorem, the three reconstructed representations are topologically diffeomorphic to the original system, ensuring the existence of one-to-one mappings among them.

2 Time series-based reconstruction in the Lorenz and Chen system

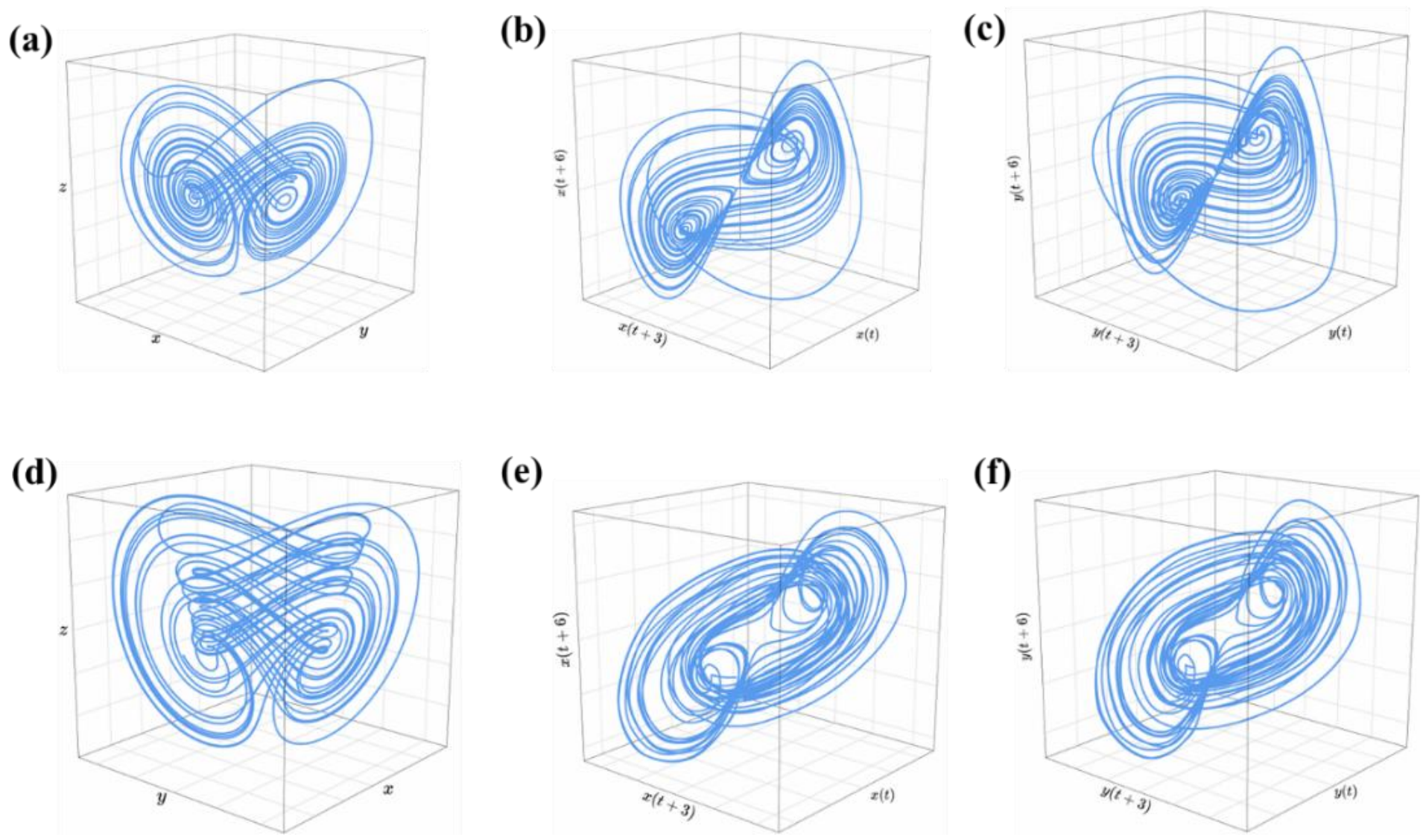

Figure S2. Time series-based reconstruction in benchmark chaotic systems. (a) Original 3-dimensional Lorenz system. (b, c) Reconstructed representations from time series x and y, respectively, where $E = 3, \tau = 3$. (d) Original 3-dimensional Chen system. (e, f) Reconstructed representations from time series x and y, respectively, where $E = 3, \tau = 3$. The attractors of chaotic systems are well reconstructed from their output time series.

3 Imputation in the 3D ecology system

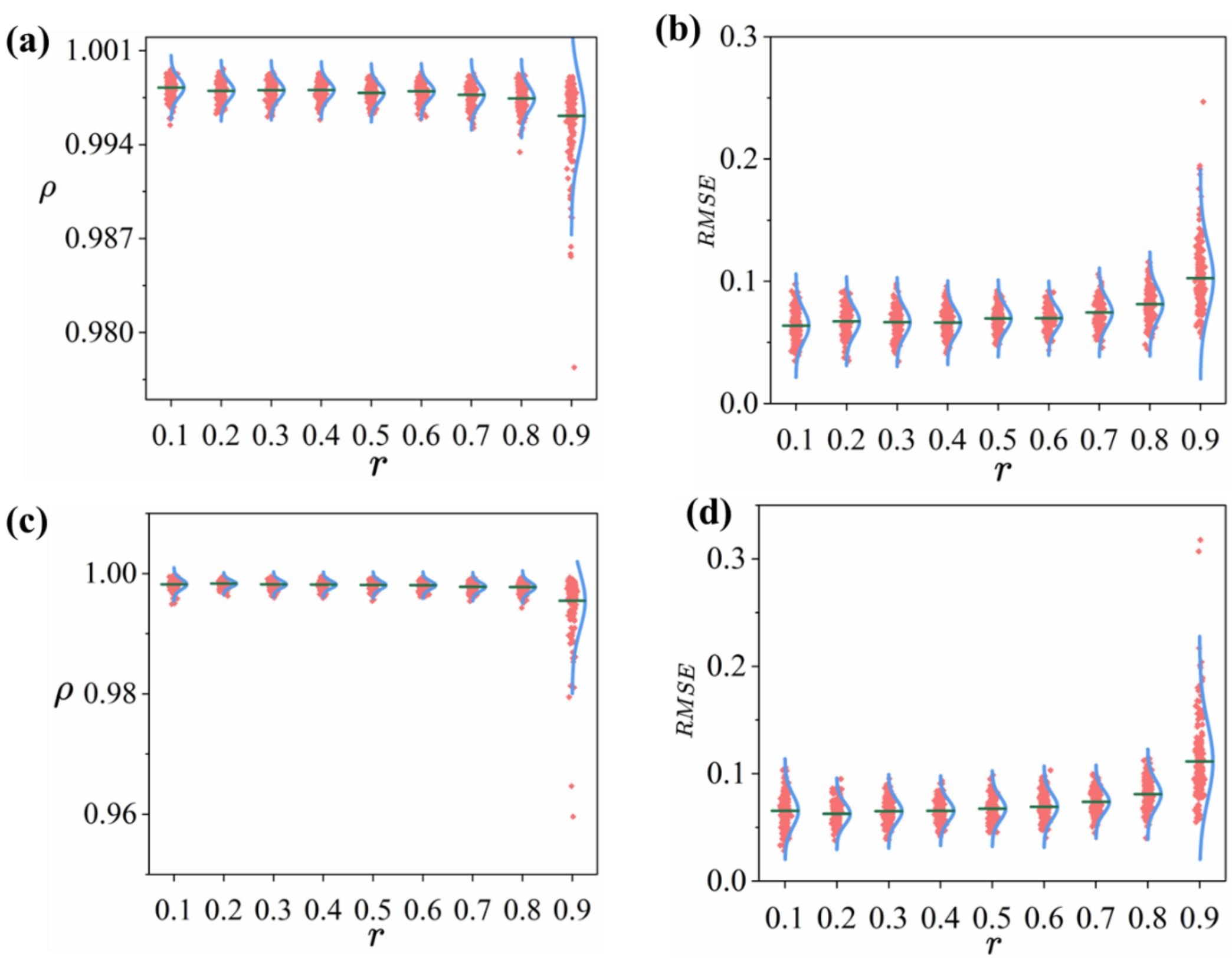

Figure S3. Imputation of incomplete series in 3-dimensional ecology system. (a, b)

Imputation performance of variable x with increasing missing elements, where variable y is complete and used for the imputation. (c, d) Imputation of variable z using complete variable y. Our method achieves reliable imputation of substantial missing elements in this nonlinear system. Note: The distribution is derived from 200 repeated experiments. r represents the proportion of missing values, and ranges from $r = 0.1$ to $r = 0.9$.

4 Imputation in the 3D Lorenz system

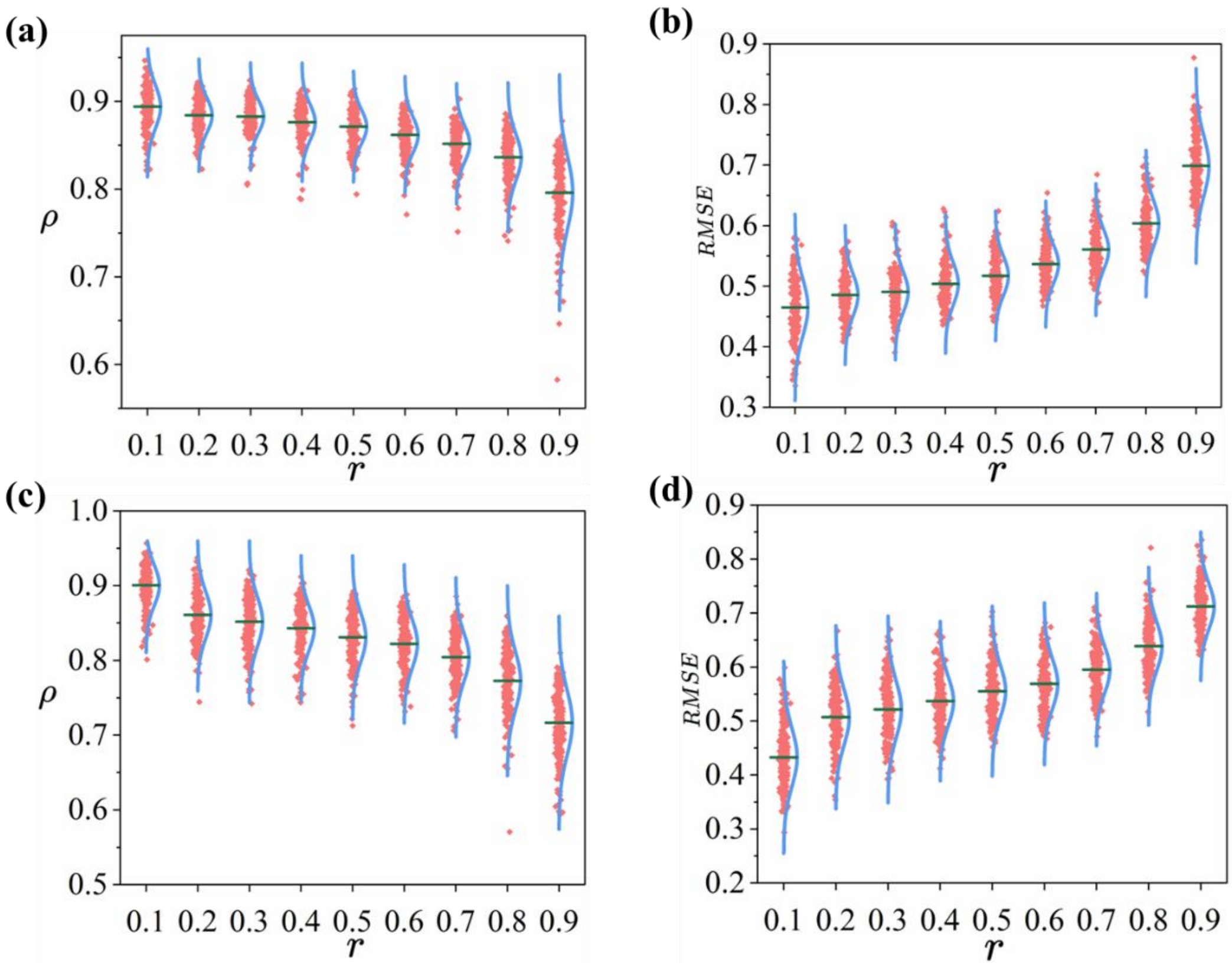

Figure S4. Imputation of incomplete series in 3-dimenisonal Lorenz system. (a, b) Imputation performance of variable x with increasing missing elements. (c, d) Performance of variable z. Note: Variable y is assumed to have complete information. The distribution is derived from 200 repeated experiments. r represents the proportion of missing values, and ranges from $r = 0.1$ to $r = 0.9$.

5 Robustness test for length of data

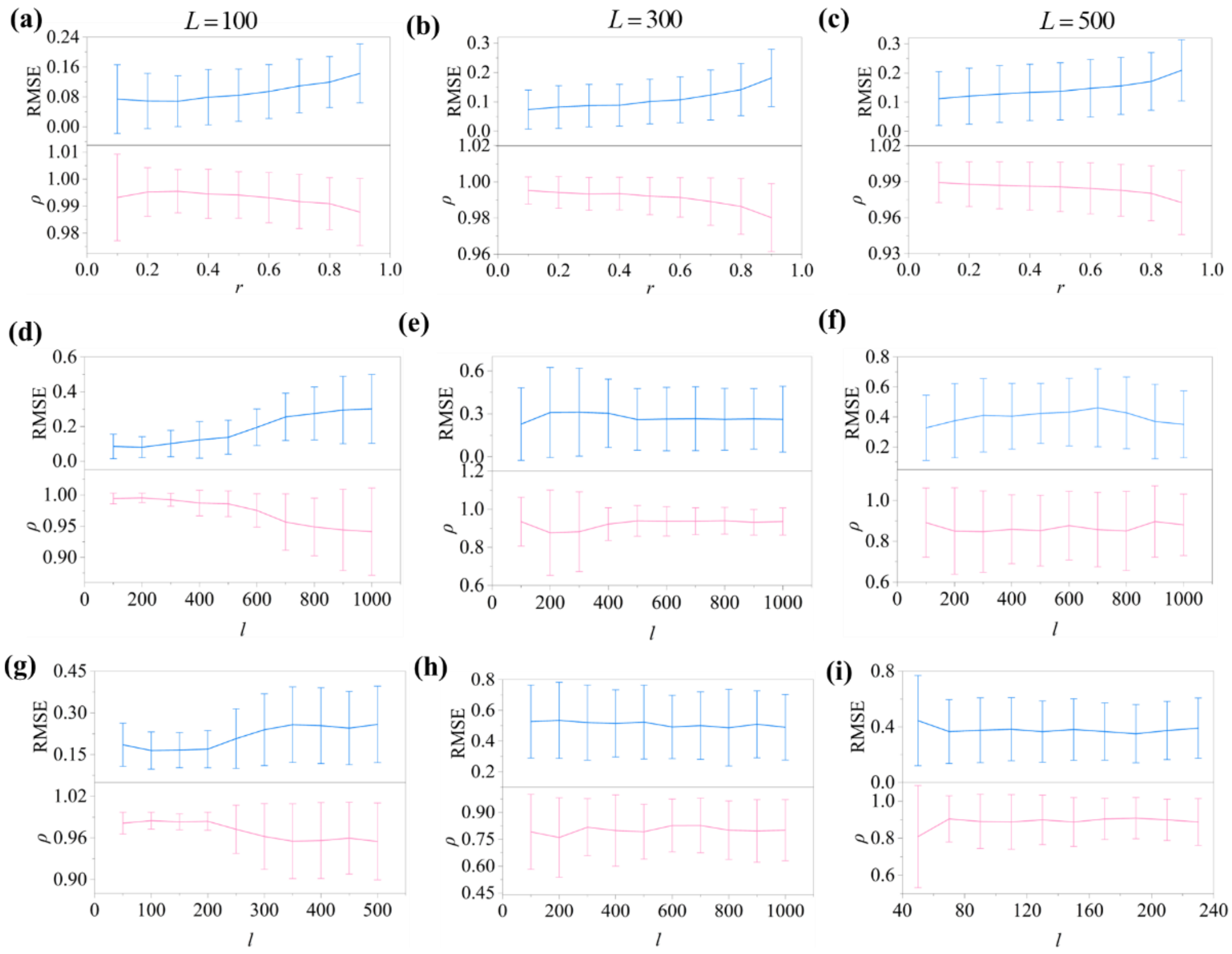

Figure S5. Robustness test of length of datasets. (a-c) Performance for the 3D Lorenz system under different time series lengths ($l = 100, 300, 500$) and missing rates ranging from $r = 0.1$ to $r = 0.9$. (d) Performance for the coupled Lorenz system as the time series length increases from $l = 100$ to $l = 1000$, with a fixed missing rate of $r = 0.5$. (e) Corresponding analyses for the coupled Rössler system. (f-i) Robustness tests on real-world datasets, including road occupancy rates, EEG signals, exchange rates, and wind speed series, consistent with the datasets used in the main manuscript. Note: In all tests, only one variable is assumed to be fully observed, while all other variables contain missing values. Error bars represent the standard deviation of the evaluation metrics across repeated trials.

6 Robustness test for colored noise

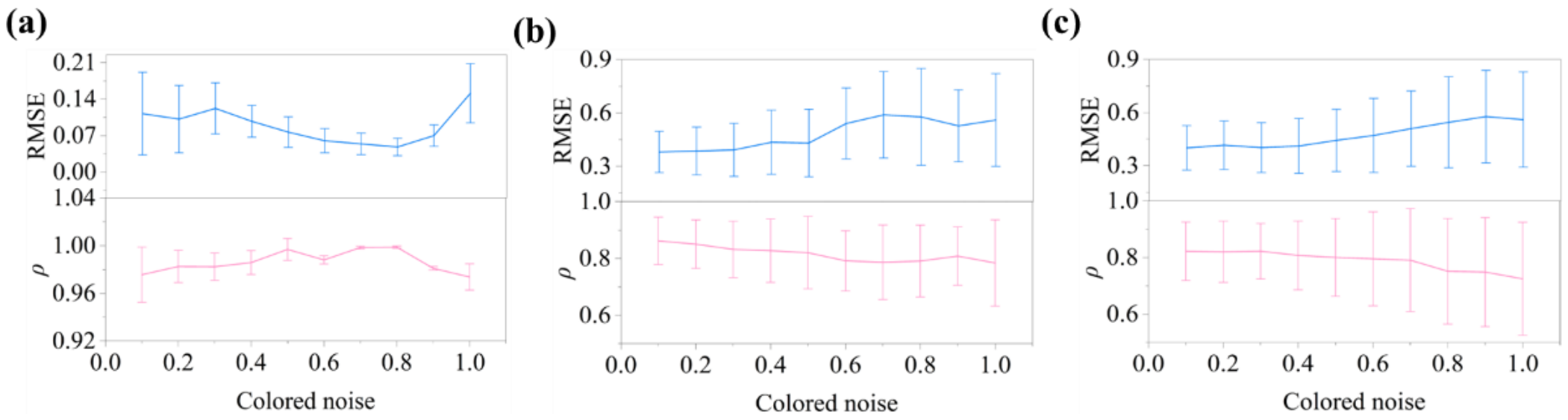

Figure S6. Robustness test under colored noise. (a) Performance for the 3D ecology

system as the colored noise intensity increases from 0.1 to 1. (b) Performance for the 120D coupled Lorenz system. (c) Performance for the 120D coupled Rössler system. Note: the colored noise is simulated via a typical $AR(1)$ autoregressive model: $\eta(t) = \upsilon\eta(t-1) + \varepsilon(t)$, where $\varepsilon(t)$ is the white noise, and υ is the correlation parameter (we set $\upsilon = 0.8$ in this work).

7 Imputation in the 3D Chen system

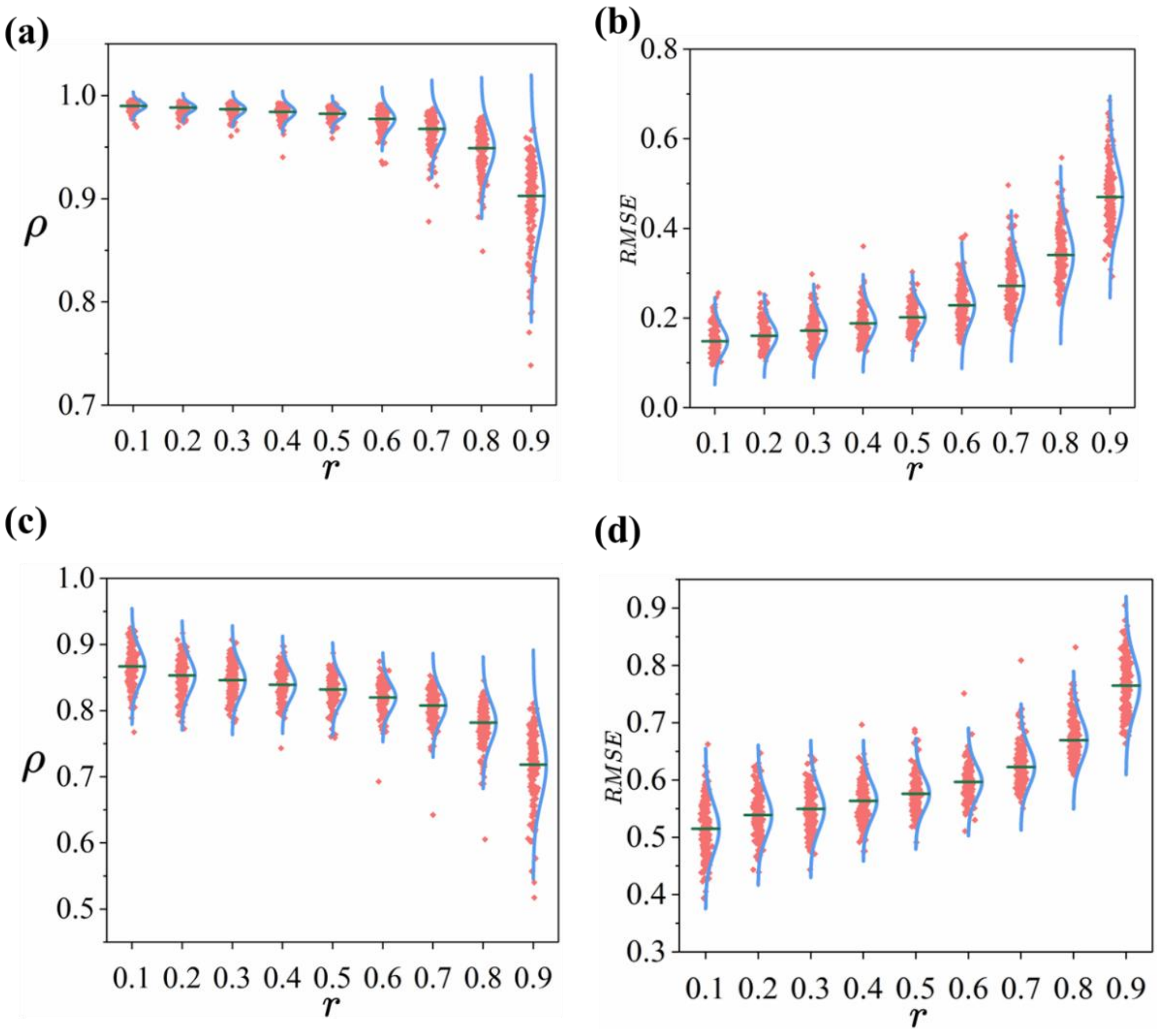

Figure S7. Imputation of incomplete series in 3-dimenisonal Chen system. (a, b) Imputation performance of variable y with increasing missing elements. (c, d) Performance for variable z. Note: Variable x is assumed to have complete information.

8 Imputation of real-world data from climate system

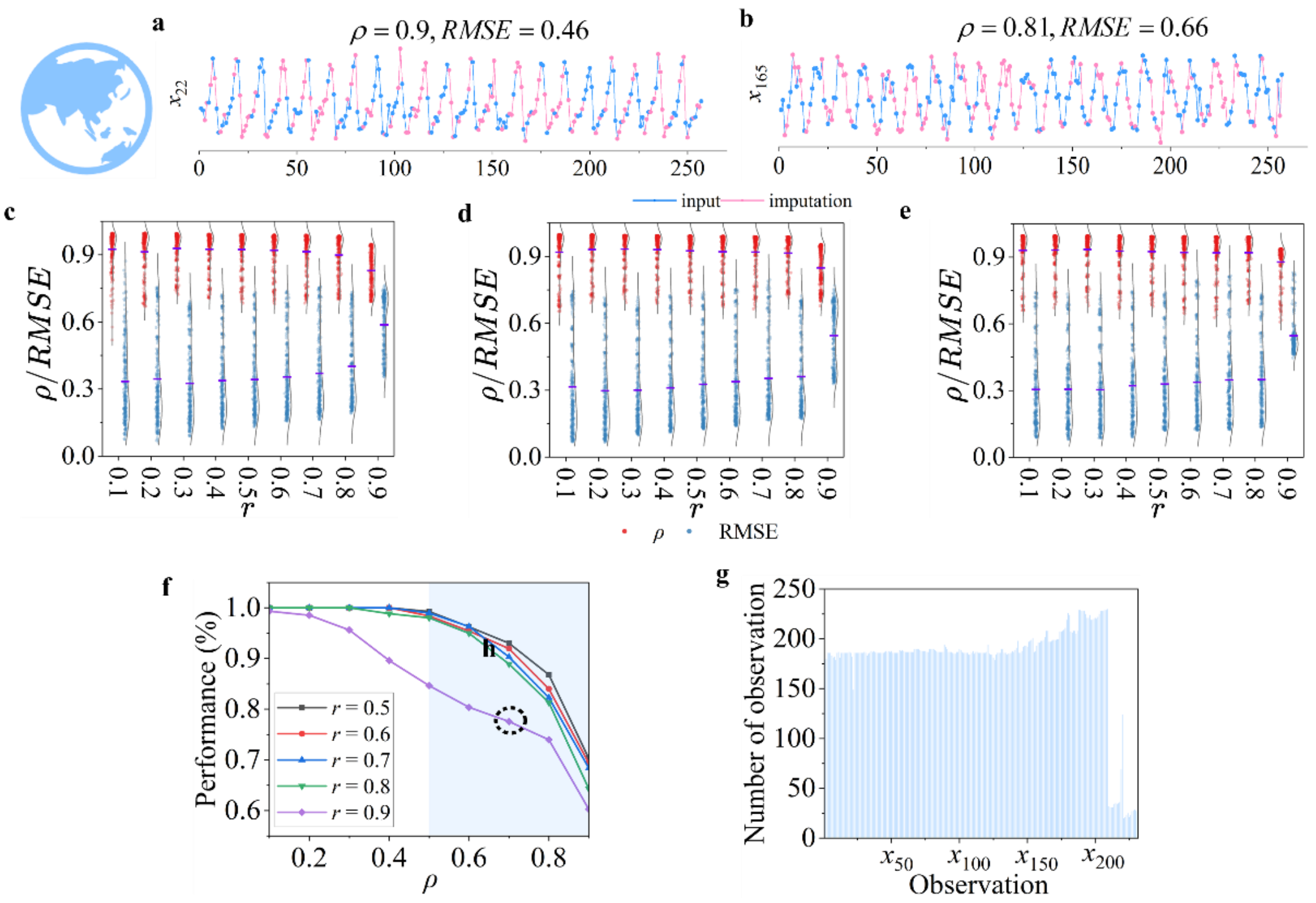

Figure S8. Imputation of wind speed. (a, b) Imputations for randomly selected wind speed series x_{22} and x_{165} in the climate system ($r = 0.5$), where x_1 is assumed complete series and used to construct the model input. (c-e) Imputations for all incomplete series when only one variable is fully observed (e.g., x_{31}, x_{61}, or x_{91}). The performance metrics for 230 incomplete observations with increasing missingness proportions from $r = 0.1$ to $r = 0.9$ are presented as red (ρ) and blue (RMSE) plots. (f) Overall imputation performance (measured by the ratio of actual connections in the underlying network) as missingness increases from $r = 0.5$ to $r = 0.9$.(g) Imputation capacity (the number of available imputed variables) for each variable in the climate system. Each bar represents the number of available imputed variables for a given complete variable, where all other variables contain 90% missingness and the imputation performance satisfies $\rho \geq 0.7$ (corresponding to the case denoted by the dashed coil in (f)).Note: We select the lower-tropospheric (850 hPa) zonal monthly wind component from the Indian Monsoon Index 2 (IMI2) region (700E–900E, 200N–300N), provided on a spatial grid with a resolution of $1^0 \times 1^0$. Collected over the period from 1998 to 2019, this dataset yields 231 time series (denoted as $x_1, x_2, \ldots, x_{231}$), with each series consisting of 264 state points.

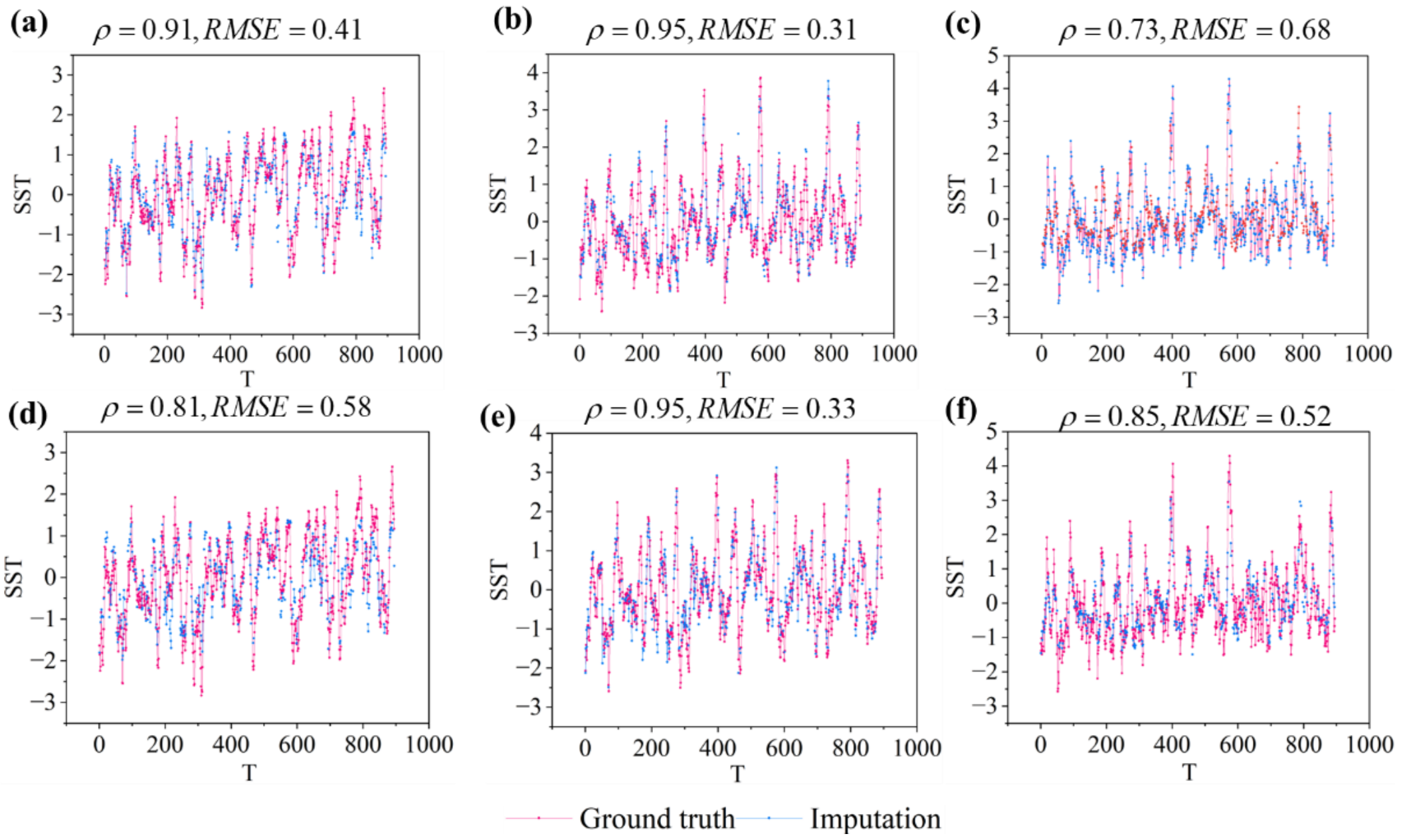

Figure S9. Imputation of sea surface temperature (SST) in ENSO cycle. (a-c) SST imputations in the ENSO 4, ENSO 3, ENSO 1+2 regions, with only SST in ENSO 3.4 region complete. (d-f) SST imputations in ENSO 4, ENSO 3.4 and ENSO 1+2, with only SST in ENSO 3 region complete. Note: The proportion of missing elements is 0.5. Results demonstrate the efficiency of FPRM framework. Note: Monthly SST anomaly series from 1990/01 to 2025/02 were selected, and these data are freely available at https://www.cpc.ncep.noaa.gov.

9 Imputation for continuous missingness

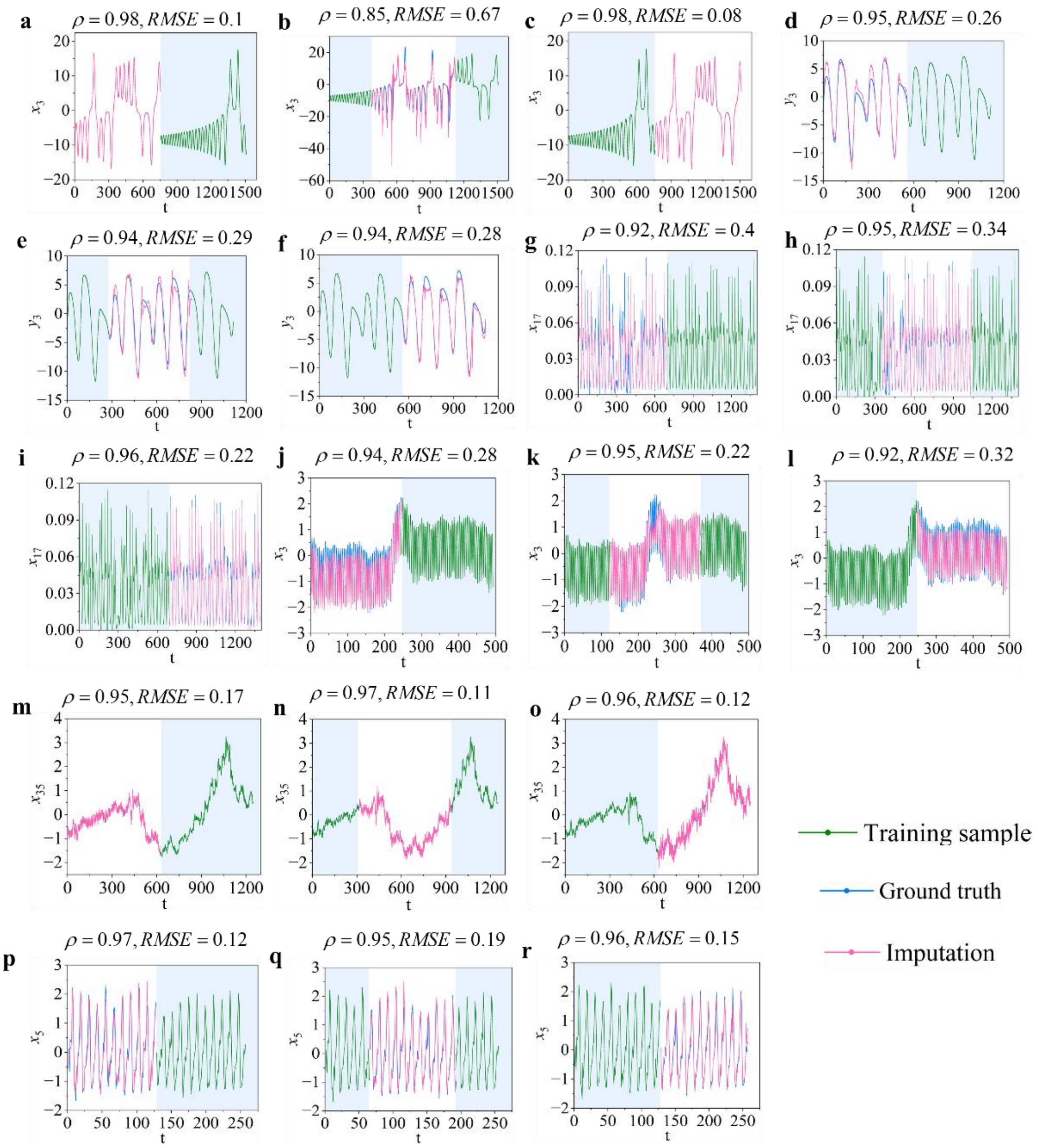

Figure S10. Imputation of consecutive missing values. (a-c) Imputation for the 120D Lorenz system, where the first, middle, and last halves of elements are missing, respectively. (d-r) Corresponding cases for the 120D Rössler system, road occupancy rates, EEG signals, and exchange rates, wind speeds, respectively. Note: the first variable (x_1) is assumed to be complete, and its associated full reconstruction is used as model input. The series covered by the shaded area represents the training samples (complete series), and the remaining series represent the test samples (missing values).

10 Imputation for threshold-induced missingness

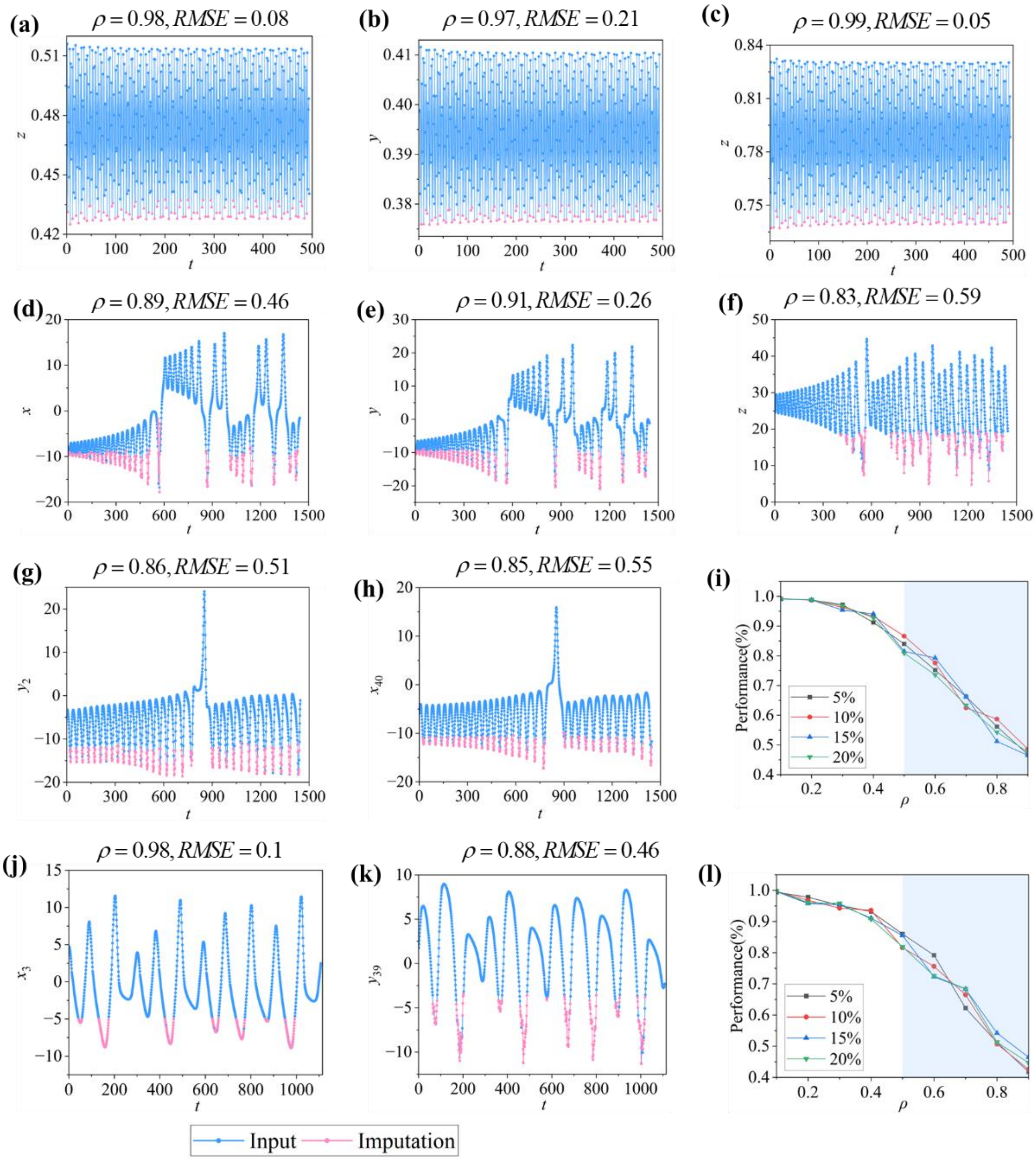

Figure S11. Imputation performance for threshold-induced missingness in model systems. (a-c) Results for the 3D ecology system, where values below the 20% quantile threshold are set as missing. (d–f) Corresponding analyses for the 3D Lorenz system. (d-f) Corresponding analyses for 3D Lorenz system. (g-i) Imputation performance for random selected observations (e.g., y_2 and x_{40}) in the 120D coupled Lorenz system. (i) Overall imputation performance in the 120D coupled Lorenz system when only one variable is fully observed while all other variables suffer from threshold-induced missingness at quantile levels of 5%, 10%, 15%, and 20%. (j-l) Corresponding analyses for the 120D coupled Rössler system.

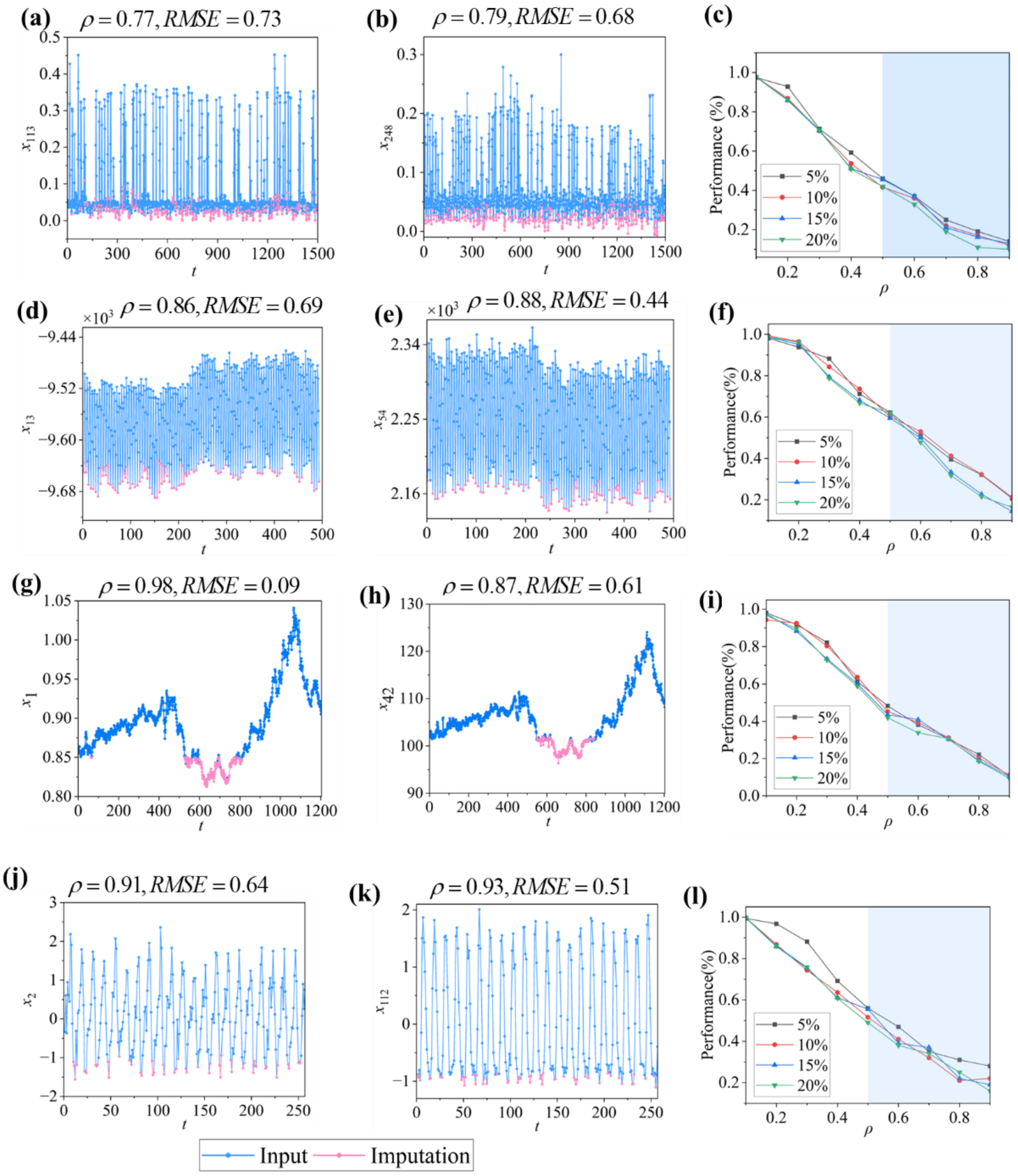

Figure S12. Imputation of threshold-induced missingness in real-world systems. (a–b) Imputation performance for road occupancy rates series. (d–f) Imputation performance for EEG series. (g–i) Imputation performance for exchange rate series. (j–l) Imputation performance for wind speed series. Note: The first two columns correspond to the scenario where data values below the 20% quantile threshold are set to missing.

11 Imputation for periodic missingness

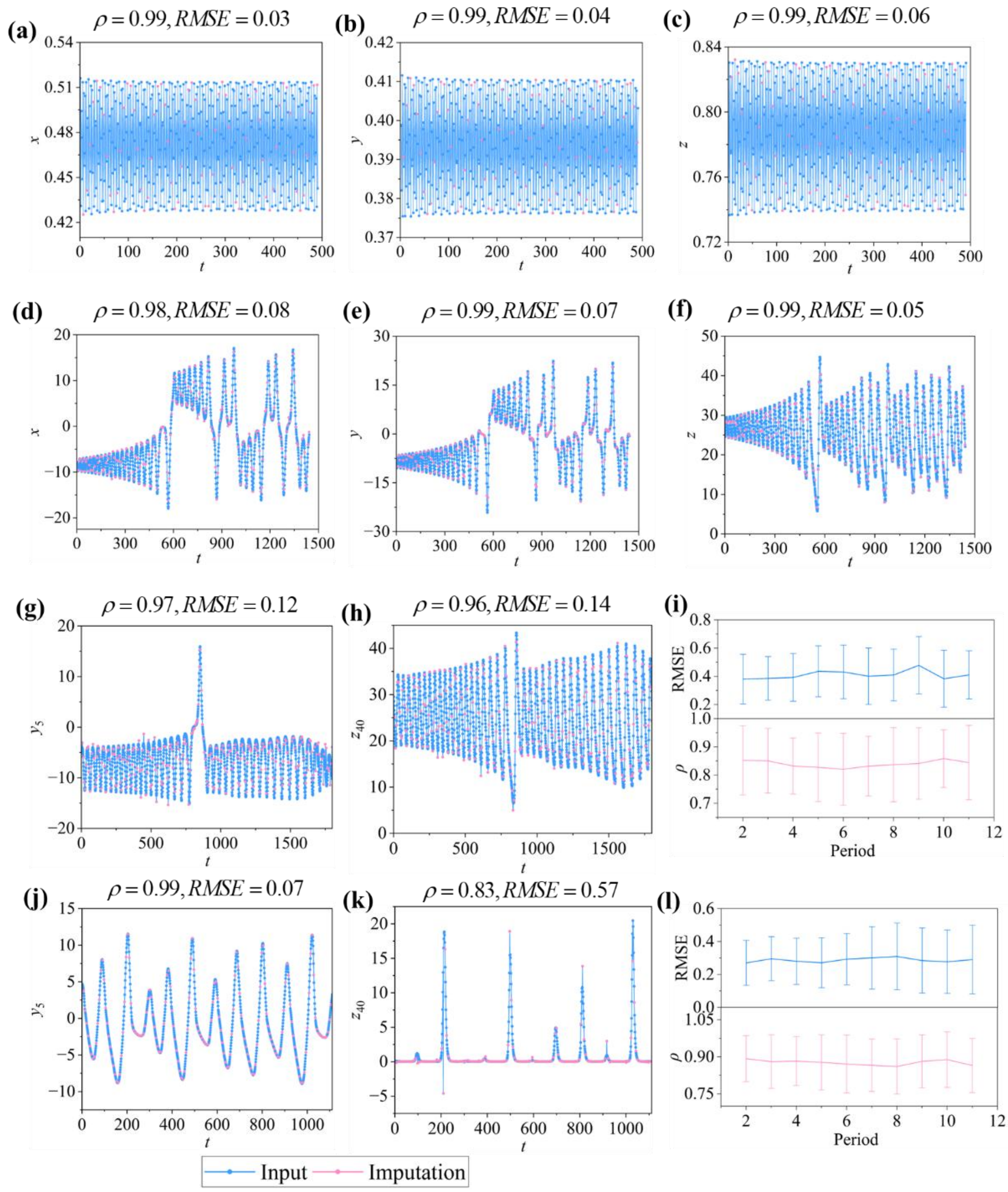

Figure S13. Imputation of periodic missingness in model systems. (a-c) Performance for the 3D ecological system, where data points are missing at a fixed interval of 7 sampling steps. (d-f) Corresponding analyses for the 3D Lorenz system. (g-i) Imputation performance for random selected observations, e.g., y_5 and z_{40} in 120D coupled Lorenz system. (i) Overall imputation performance (mean values with standard deviation error bars) for the 120D coupled Lorenz system when only one variable is fully observed while all others exhibit periodic missingness at fixed intervals ranging from 2 to 11 sampling steps. (j-l) Consistent analysis for the 120D coupled Rössler system.

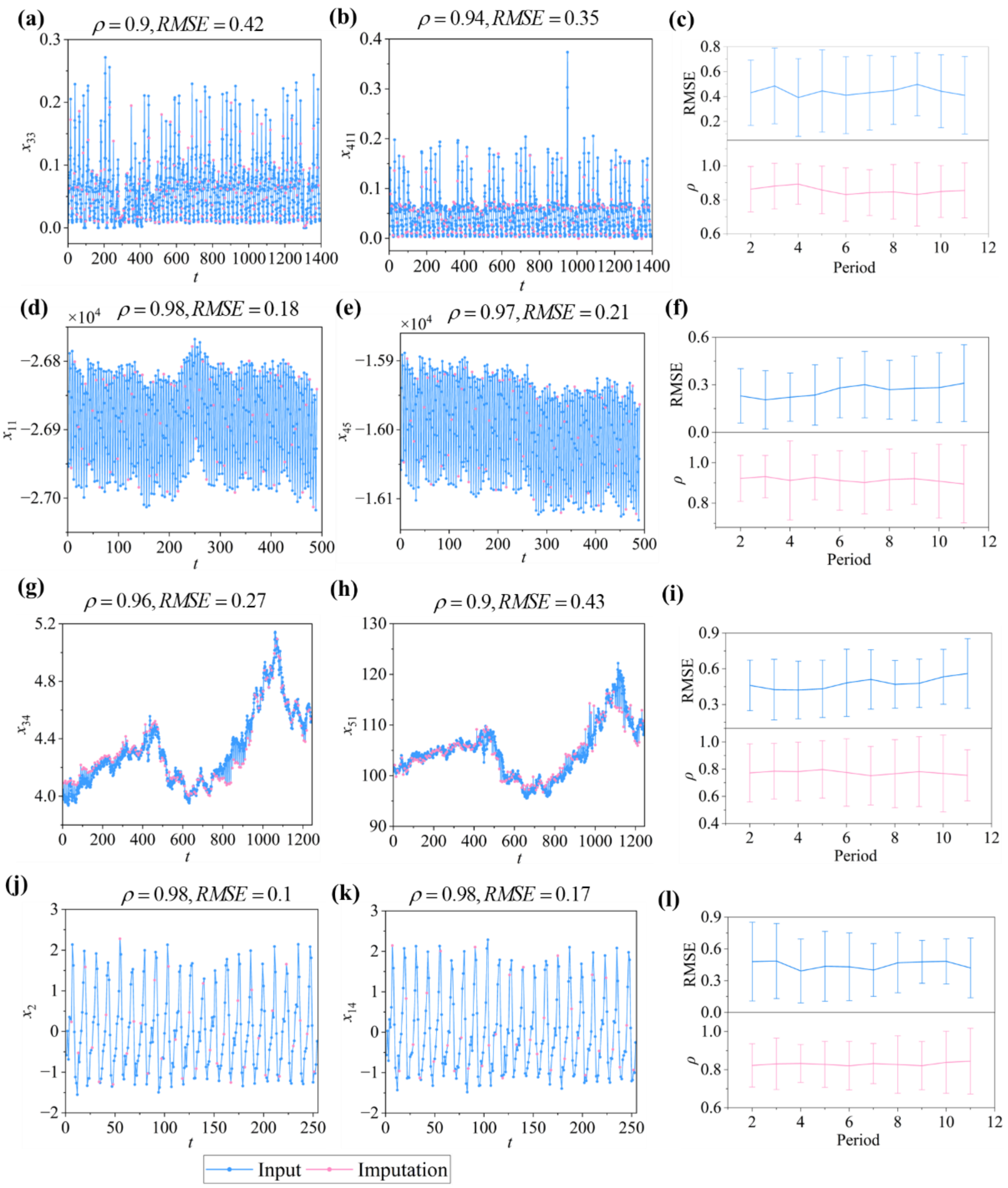

Figure S14. Imputation of periodic missingness in real-world systems. (a–c) Imputation performance for road occupancy rates. (d–f) Imputation performance for EEG series. (g–i) Imputation performance for exchange rate series. (j–l) Imputation performance for wind speed series. Note: The first two columns correspond to the periodic missing scenario with a fixed interval of 7 sampling steps.

12 Imputation framework for simultaneous missingness of all variables

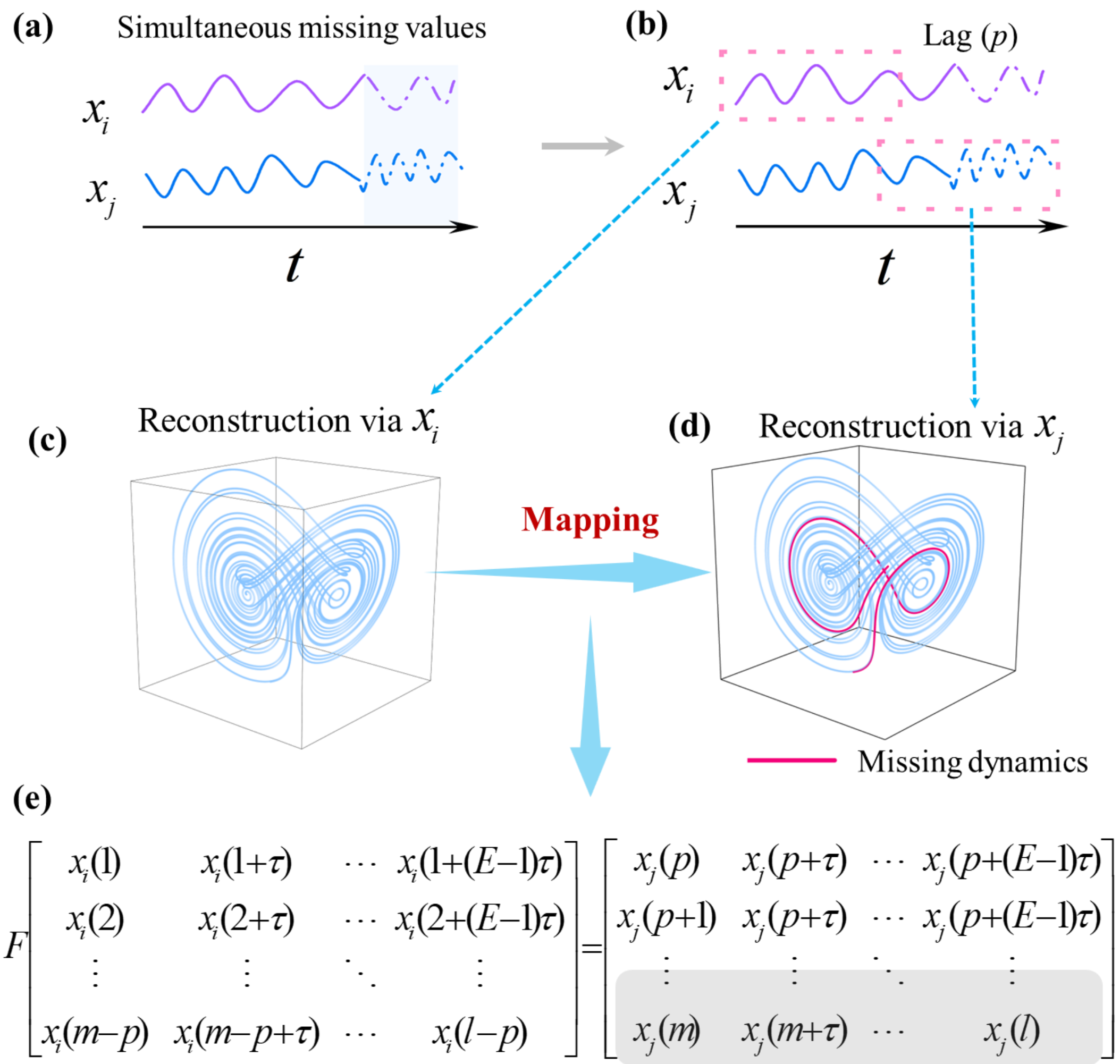

Figure S15. Imputation framework for simultaneous missingness of all variables. Suppose all observations contain simultaneous missingness (shadow areas in (a)), we also construct full reconstruction (b, c) from complete time series and partial reconstruction from lag time series. (e) Then, we can impute missing values (shadow elements) via a one-to-one mapping from full to partial reconstruction.

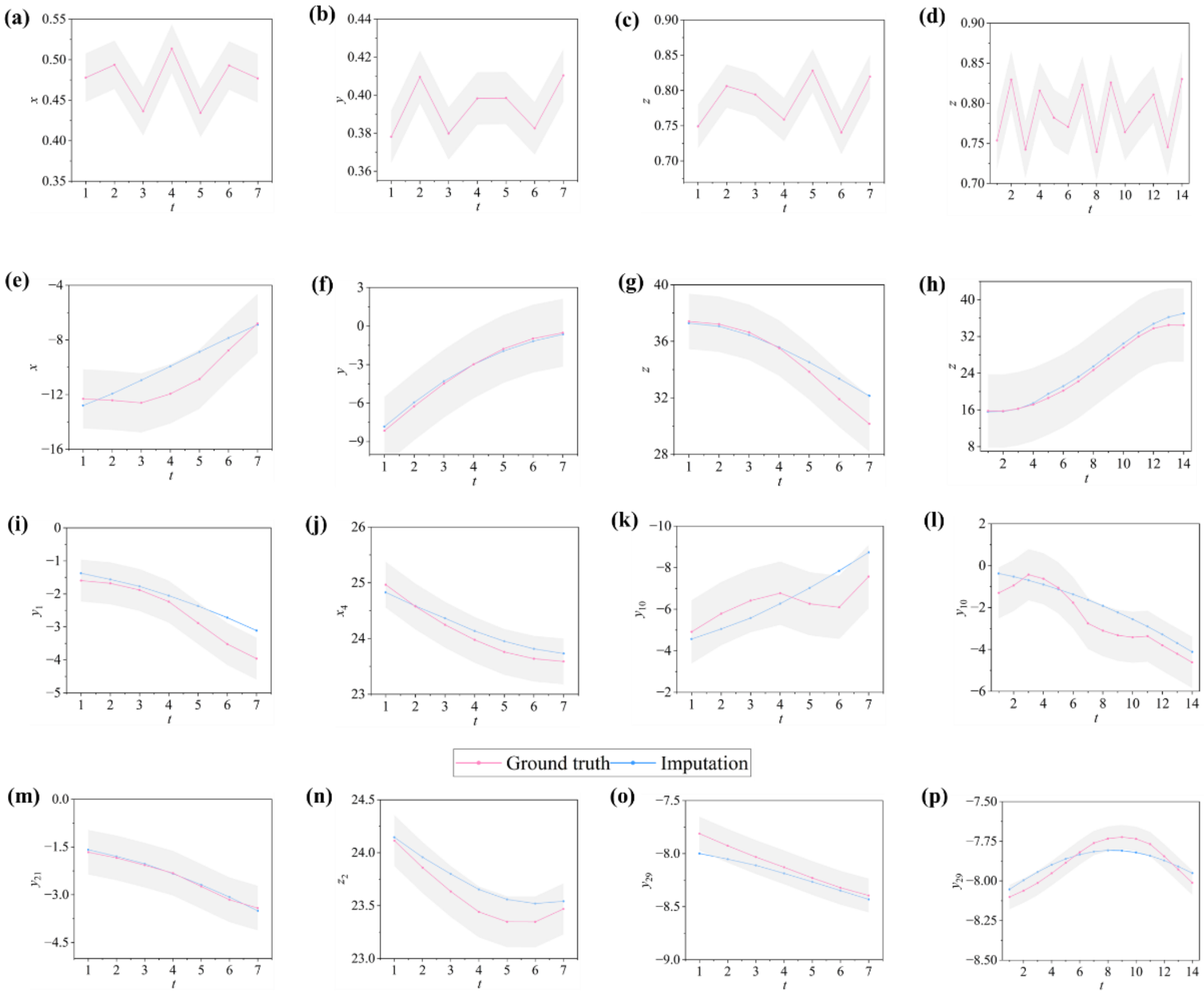

Figure S16. Imputation of simultaneous missingness of all variables in model systems. (a-d) Performance in 3D ecology system, where the number of missing points for each variable are seven (a-c) and fourteen (d). (e-h) Similar cases for the 3D Lorenz system. (i-l) Performance in the 120D coupled Lorenz system. (m-p) Similar cases for the 120D coupled Rössler system. Note: the first three and final columns simulate the numbers of missing points for each variable are seven and fourteen, respectively.

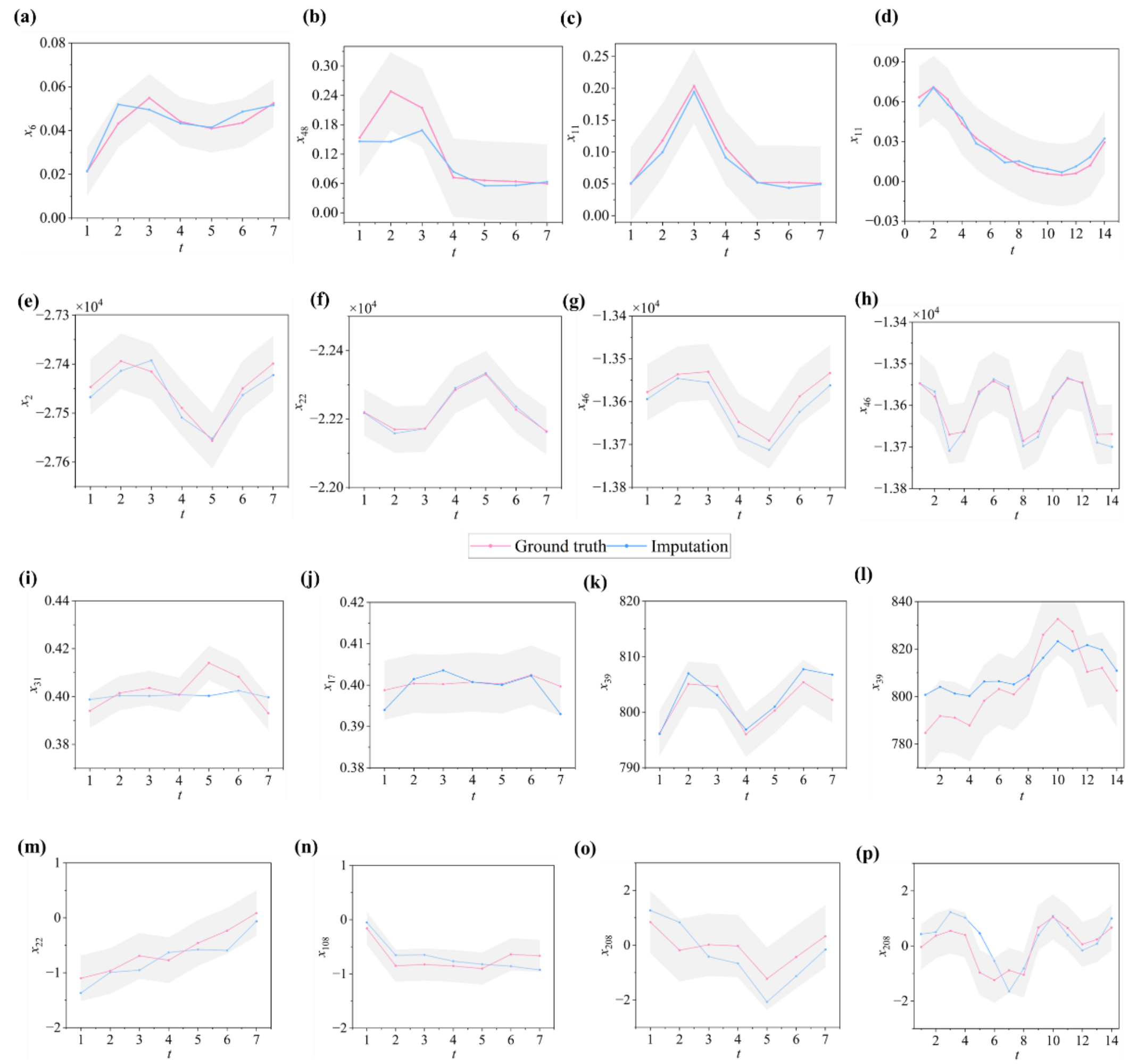

Figure S17. Imputation of simultaneous missingness of all variables in real-world systems. (a-d) Performance for road occupancy rates. (e-h) Performance for EEG series. (i-l) Performance for exchange rate series. (m-p) Performance for wind speed series. Note: the first three and firnal columes simulate the numbers of missing points for each variable are seven and fourteen, respectively.

13 Imputation under different embedding dimension

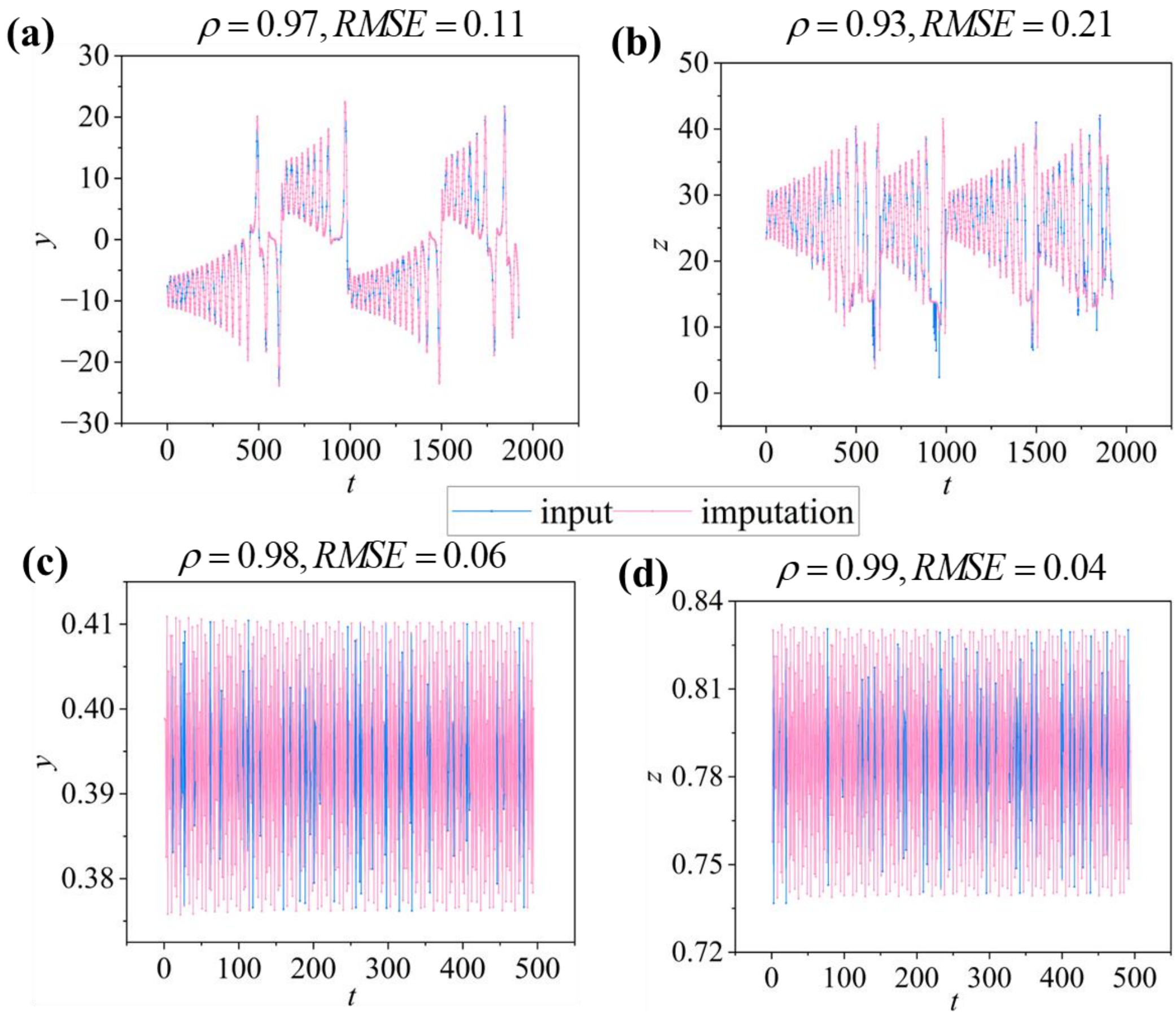

Figure S18. Imputation in low-dimensional space. (a, b) Imputation results for the 3D Lorenz system, in which variables y and z are incomplete with 90% missingness. The dimension of both full and partial reconstruction (i.e., 2D) is lower than that of the original system. (c, d) Similar imputation scenarios are shown for the 3D ecological system.

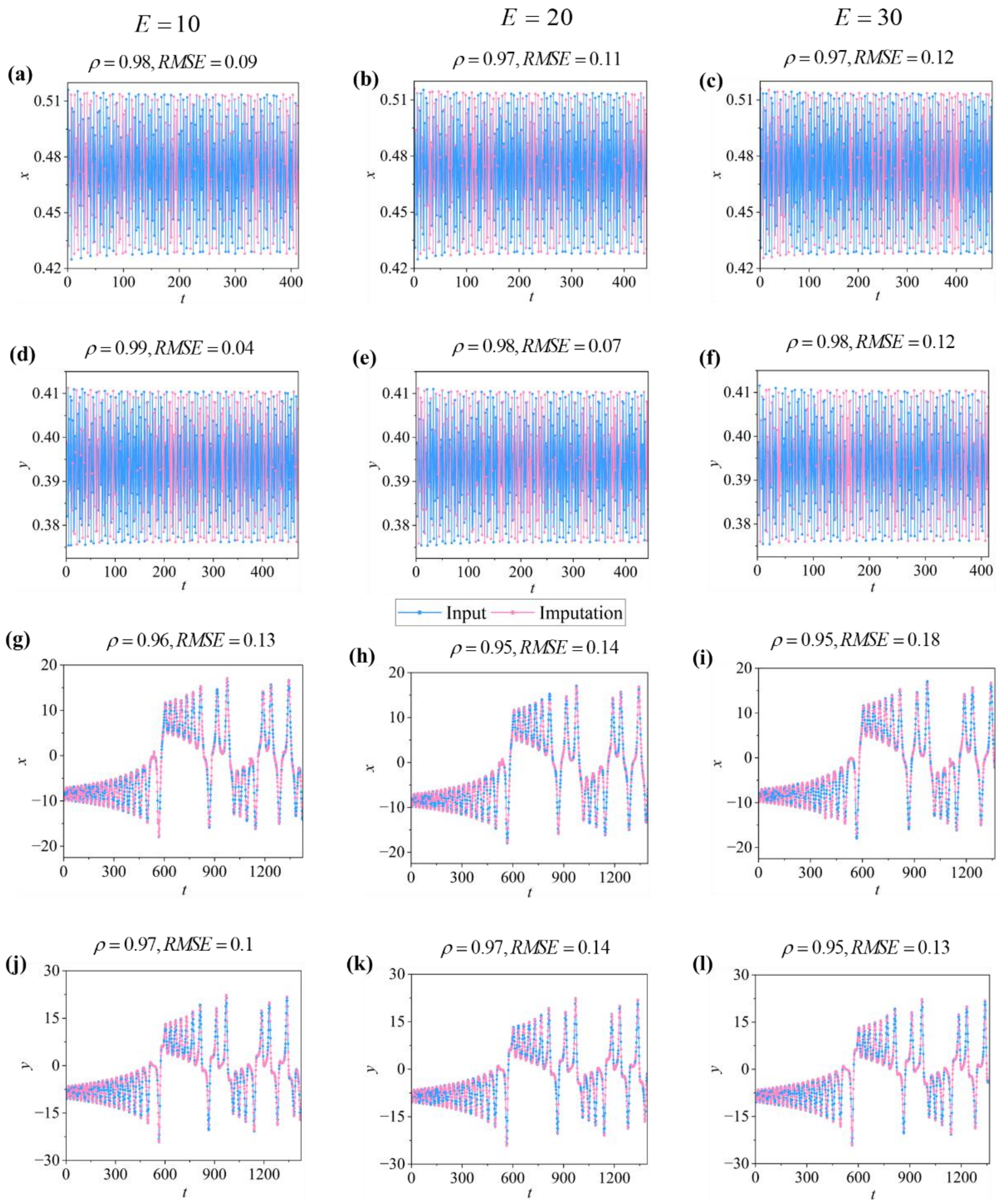

Figure S19. Imputation in multi-dimensional space. (a-f) Imputation results for the 3D ecology system with different embedding dimension, in which variables x and y are incomplete with 50% missingness. (g-l) Similar imputation scenarios are shown for the 3D Lorenz system. We first conduct phase-space reconstruction in a multidimensional space ($E = 10, 20, 30$) and then implement missing value imputation. The results further demonstrate the reliability of the proposed method.

Supplementary tables

1 Embedding dimension and time lag for delay embedding

Table S1. The parameters for attractor manifold reconstruction

Sample	Embedding dimension (*E*)	Time lag (τ)
3D Ecology system	3	3
3D Lorenz system	3	3
120D Lorenz system	4	3
120D Rössler system	5	2
500D road occupancy rates	6	4
64D EEG signal	3	3
52D exchange rates	4	4
4D SST series	3	3
231D wind speed	4	6

Note: The embedding dimension is identified by the popular false nearest neighbor (FNN) method [1]. The time lag is estimated from mutual information [2].

2 Correlation dimension for datasets

Table S2. Estimation of correlation dimension in our sample

Datasets	Correlation dimension
3D ecology system	1.31
3D Lorenz system	2.12
120D coupled Lorenz system	10.97
120D coupled Rössler system	12.13
500D road occupancy rate	15.04
64D EEG series	6.24
52D exchange rate	8.93
231D wind speed	11.03

Supplementary notes

1 Main imputation pipeline of the S-map algorithm

In our benchmark setting where only one anchor time series is fully observed and all remaining variables suffer 90% data missingness, S-map reconstructs attractors solely from this complete reference and imputes missing entries of coupled incomplete series via exponential-weighted local linear regression over all valid attractor states. Specifically, we first perform state-space reconstruction with fixed embedding dimension E and time delay τ whose values are kept identical to those adopted in the FPRM framework (Table S1). During embedding construction, we only utilize the single fully observed time series to build delay-coordinate attractors, and discard any state vectors that contain lagged values from incomplete variables to form the valid training library. The only tunable hyperparameter of S-map is the locality weight θ, which we searched over $\{1,5,9,15,25,35,45\}$ following the standard benchmark range defined in the original S-map algorithm and rEDM toolbox ((https://github.com/SugiharaLab/rEDM). The model assigns exponential weights $w(d)=\exp(-\theta d/\bar{d})$ to valid library states based on the Euclidean state-space distance. For each time point with missing entries, we build a query attractor state solely from lagged values of the complete series. A weighted local linear regression is fitted on all valid library points, and the optimal θ is selected to maximize the Pearson correlation between ground truth and imputed values.

2 Main imputation pipeline of the MVCM algorithm

In our benchmark setting where only one anchor time series is fully observed and all remaining variables suffer 90% data missingness, MVCM extends the single-view S-map paradigm and achieves imputation by ensembling predictions from multiple attractor views built upon the sole complete reference series. we first perform state-space reconstruction with fixed embedding dimension E and time delay τ whose values are kept identical to those adopted in the FPRM framework (Table S1). For the multi-view construction, we sample distinct lag combination for the candidate lag rang [1, 20] to generate diverse attractor views, and eliminate any candidate view containing lagged values from incomplete variables. The core tunable hyperparameters of MVCM include the locality weight θ (consistent with S-map) and the number of top-performing view n. Each view produces independent S-map predictions with exponential distance weighting, and outputs are aggregated via weights proportional to

each view's cross-mapping predictive skill. For each time point with missing entries, we build a query attractor state solely from lagged values of the complete series. A weighted local linear regression is fitted on all valid library points, and the optimal θ is selected to maximize the Pearson correlation between ground truth and imputed values. For each timestamp with missing measurements, we filter valid lag-based attractor views derived from the complete anchor series, run S-map local regression for each qualified view, and compute the weighted ensemble average across top-*n* views. The optimal combination of θ and *n* is selected to maximize the Pearson correlation coefficient between ground-truth observations and ensemble imputed predictions.

Supplementary references

1. Krakovska, A., Mezeiova, K. & Budacova, H. (2015) Use of false nearest neighbors for selecting variables and embedding parameters for state space reconstruction. *Journal of Complex Systems*, 2015:932750.
2. Fraser & Swiney. (1986) Independent coordinates for strange attractors from mutual information. *Physical Review A*. 33:134-1140.
