# Supplementary material for: Dynamics-informed machine learning for recovering extensive missing systems dynamics
Source: Nat Commun. 2026 Sep 19;17:10986. doi: 10.1038/s41467-026-77922-1 (PMC13683343; doi:10.1038/s41467-026-77922-1)
Supplement: Supplementary file 2 [file 41467_2026_77922_MOESM2_ESM.pdf]

nature portfolio

Peer Review File

Dynamics-Informed Machine Learning for Recovering Extensive Missing Systems Dynamics

Corresponding Author: Dr Tao Wu

This file contains all reviewer reports in order by version, followed by all author rebuttals in order by version.

Version 0:

Reviewer comments:

Reviewer #1

(Remarks to the Author)
In their manuscript “Dynamics-Informed Machine Learning for Recovering Extensive Missing Systems Dynamics” Wu and co-authors describe an approach to filling in gaps in time series based on time delay embedding and manifold learning. The manuscript demonstrates the algorithm in a wide range of simulated and empirical time series including atmospheric data, EEG data, and financial time series. Overall, the method appears to work quite well, the figures are very nicely done, and the paper is well-written.

The authors are primarily focused on values that are missing at random – which is a good place to start. However, there are many situations in which variables exhibit state-dependent or highly correlated missingness. For instance, values that are reported missing when the true value falls below a detection threshold are common in many disciplines. What happens when all of the variables have missing values at the same time? This happens frequently in real world data, e.g. when a satellite, camera, or other sensor is offline for repairs or maintenance, or there is a storm that prevents data collection, etc. These practical situations do not seem to be addressed. Also, it seems like 90% missing still corresponds to hundreds of complete observations. What happens when you have fewer – say 10’s?.

The monsoon example is interesting, but makes me wish that the authors had included a more reasonable alternative model as a benchmark. As the authors are aware, data imputation is a well-established field - particularly in time series analysis. Showing that this new method works is nice and showing that it works against a few alternatives is also good. But showing that it works better than the state of the art would make it really exciting. The comparison models are all static ML – which is pretty far from the best one can do with time series data. For example, atmospheric data are typically highly autocorrelated in space and time. This suggests that a linear spatio-temporal imputation scheme – even something as simple as the Beale-Little algorithm developed to accommodate missing values in principal component analysis in the 1970’s (using lags to make the comparison fair) – would suffice. I recognize that the method the authors have presented is more general than this linear imputation method, but readers, like me, typically want to know if the extra computation is worth the effort.

The ecological example is a bit too trivial to count as a ‘real world’ application – there are only two variables, and since these exhibit cyclic dynamics, the attractor is 1-d. In the real world, the number of species vastly exceeds 2 and attractor dimensions are typically >5. I suggest simply dropping this - there's already plenty here to make a convincing case.

This all said, I am having some trouble seeing how this algorithm differs- in a fundamental way - from the algorithm presented by Carpenter et al. (2025) ‘Repairing gaps in ecological time series’ in Methods in Ecology and Evolution. The basic idea and theoretical justification are identical. As far as I can tell, the only real difference is that Wu et al. use GP regression for state reconstruction, while Carpenter et al. use a local linear model. A trivial distinction. The authors have cited Carpenter in the context of missing variables in ecology, but have failed to note the considerable methodological overlap – or take the opportunity to highlight the differences in their approach. This seems critical if this paper is to appear in Nature.

Two small points by line number-

285-I am glad to see the authors move beyond the trivial Lotka-Volterra and Lorenz examples. It would be helpful in interpreting the results if-in addition to the dimensionality of the state space- the authors provided an estimate of the attractor

dimension. E.g. if the attractor dimension is 1-2 as in the previous examples, then this isn't that much more challenging. But if it is 50 or more, then the results are quite impressive.

529-530 – again the authors assert that static methods for data imputation are the only possibility, overlooking well established time series methods.

(Remarks on code availability)
Some of the code are available at this link - but not enough to rerun the examples. E.g. the mlx code seems to references a function PhaSpaRecon which is not provided, similarly the file for the high dimensional Rossler example is missing. These are only two examples, not a thorough list of all of the things that are missing that would be required to repeat this analysis.

Reviewer #2

(Remarks to the Author)
The manuscript presents an imputation framework for recovering missing values in a multivariate time series data from a known system, based on knowledge of system dynamics and the availability of complete time series for at least one variable. The framework relies on Takens' embedding theorem, which provides a mathematical basis for the approach's validity in principle. The presentation of the results is clear, and the selection of test systems is diverse and appropriate. However, the framework's positioning as a general data-driven modelling approach appears somewhat overstated, given its reliance on a priori knowledge of the system dynamics. In addition, the statistical analysis would benefit from modest improvements in both presentation and depth. Overall, the proposed framework is promising and demonstrates advantages over model-agnostic imputation approaches.

Specifically, I have the following comments and suggestions:

1. The introduction frames the scope as "data-driven modelling" in the early sections. e.g. lines 82-90: "the reliable reconstruction of such dynamics may be compromised when the time series contains substantial missing values..." . This framing suggests that the primary objective is the discovery of underlying system dynamics and, ultimately, improved model identification. However, the framework addresses a different problem: the recovery of missing values in observational time series for a system whose dynamics are assumed to be already known. This distinction is important and should be clarified. The authors are encouraged to revise the Introduction to more accurately reflect the scope of the work.
2. The comparison with existing methods focuses primarily on model-agnostic or system-unaware approaches, which implicitly combine model learning and imputation (e.g., machine learning, deep learning, or Bayesian methods). While such comparisons are useful from a practical standpoint, the manuscript would benefit from discussing comparisons with other dynamics-informed or system-aware approaches, if available. This is only a suggestion.
3. Introduction, lines 91-93: It is not clear what 'deletion' means here. While readers familiar with imputation methods understand the technical meaning, clarifying it would improve comprehension for average readers. e.g., does it mean that the entire time series is deleted for the erratic observable?
4. Introduction (lines 104–108): The manuscript states that real-world systems are inherently nonlinear and subject to noise and time-varying shifts, and the subsequent paragraph suggests that the proposed framework addresses all of these challenges. While the claim of addressing nonlinearity is justified, nonstationarity (including time-varying shifts) is also a limitation of the proposed approach, as acknowledged later in the manuscript. This creates a degree of inconsistency in the framing. The authors may wish to moderate this claim in the Introduction.
5. Results (lines 154-186): Taken's theorem forms the basis of the proposed framework. However, its application to data relies on several assumptions, such as infinite or sufficiently long time series, an appropriate embedding dimension, and noise-free observations. While these assumptions can be approximated in practice, as demonstrated in the manuscript, this still warrants discussion of the limits with respect to the required data size. Particularly, it would help to mention the constraint on the length of the available time series relative to the system timescale. This is an important practical consideration and would help clarify the method's range of applicability.
6. Figure 3: The manuscript presents imputation results for the variable y across different noise levels, but only for a single missing data ratio (r=0.5). For completeness, it would be helpful to include analogous results for the variable z as well as for additional missing data ratios. While these results need not all be included in the main figures, they could be incorporated into Table 1 or provided as supplementary material to give a more comprehensive evaluation.
7. Figures 3 and 4: The Pearson correlation coefficient and RMSE appear to be plotted in a manner that suggests they follow the same distribution (this is particularly noticeable at higher noise levels, e.g., noise magnitude = 0.9). As these metrics represent fundamentally different quantities, this visualisation may be misleading. It would be preferable to present them separately or clearly distinguish them within the same figure, e.g., using different colours, as in Figure 6.
8. Figure 5: Connected to the previous point, the manuscript does not appear to include an analysis for the aforementioned metrics under different noise distributions for the real-world systems. Incorporating such an analysis could strengthen the interpretation of the results, particularly in relation to Figure 6, where the performance of the proposed method appears to degrade for real-world datasets, and is attributed to inherent noise. Exploring the sensitivity to different noise characteristics would provide additional insight into how to best implement the framework for practical applications.
9. Table 1: The table reports performance metrics for the compared algorithms; however, it appears that only mean values are presented. For a more rigorous statistical assessment, it would be beneficial to include measures of variability, such as standard deviations or confidence intervals. This is particularly relevant given that Figure 5 suggests a substantial spread in the distributions of these metrics, which should be reflected in the summary statistics.
10. Conclusion (lines 496–504): It is not entirely clear what is meant by "random values" in this context. The manuscript primarily discusses imputation based on Takens' embedding theorem, and it is therefore unclear what baseline or alternative this statement is referring to. The authors can clarify why and how 'random values' can be inevitable in some alternative

approaches compared.
11. Conclusion (lines 510-519): The manuscript states that the proposed framework “reconstructs a topologically diffeomorphic attractor of the system in a low-dimensional space.” This claim could be misinterpreted to mean that the framework offers a way to perform dimensional reduction from high-dimensional multivariate data. In the examples presented, the reconstructed attractor has the same dimensionality as the original system (or the number of variables for which data is available). In fact, the framework relies on matching the embedding dimension of the partially observed system to that of the full system in order to ensure a valid one-to-one mapping. As such, it may be more accurate to state that the method reconstructs a diffeomorphic representation of the attractor under appropriate embedding conditions, rather than implying dimensionality reduction. Clarifying this point would help avoid potential overinterpretation of the results.

In summary, the manuscript presents a promising and theoretically well-motivated framework, but its current presentation would benefit from clearer positioning, more precise articulation of assumptions, and more comprehensive presentation. Addressing the issues outlined above would substantially strengthen the manuscript and improve its overall rigour and clarity.

(Remarks on code availability)

Version 1:

Reviewer comments:

Reviewer #1

(Remarks to the Author)
The authors have done a nice job of responding to my earlier comments. Their efforts to address realistic patterns of missing data are commendable and results impressive. Overall, this is a very nice piece of work.

I am however, a bit disappointed by the authors response regarding MVCM. First, the authors claim that GP regression provides a global, diffeomorphic map. Maybe there is a sense in which a GP regression is 'global,' though this is rather at odds with O'Hagan's original 1978 derivation of GP regression as a Bayesian version of local linear regression. And unless the authors have implemented some incredibly clever non-standard prior specification (which they have not reported) there is no reason at all to imagine that the output of a GP regression is a diffeomorphism. To see that this is true, simply let the length scale go to zero to find that the regression collapses to a set of delta functions evaluated at the data points: Definitely not invertible.

Second, the authors claim without evidence that MVCM is innappropriate for context in which they are working. Given the description of both algorithms, I genuinely can't see why this would be the case. But the right way to respond would have been to simply run MVCM on the simulated data and show that it does not work. Instead, the authors have chosen to run S-map as a straw man (without providing any details on how this was done), which unsurprisingly performs rather poorly.

To alleviate any ambiguity: the only reasonable response is to acknowledge that Carpenter and Sugihara already had this idea, but applied it to a very narrow range of systems - right from the beginning, not buried in the Methods. They would then include Carpenter and Sugihara's algorithm alongside their own in the simulation testing so that readers had a clear sense of how significant a contribution was being made.

(Remarks on code availability)
n/a

Reviewer #2

(Remarks to the Author)
My earlier comments have been satisfactorily addressed. I am happpy to recommend the article, but I suggest a careful reading of the figure captions. For example, I noticed the caption for Figure S19 is not accurate, the panels (c)-(d) do not represent ecological system.
Another confusion is with the description of the AR1 process (Figure S6). The same symbol \rho is used for another correlation parameter everywhere else in the paper, where it represents correlation between the imputed and original time series. I suggest you check carefully and change the symbols to avoid confusion.

(Remarks on code availability)

Version 2:

Reviewer comments:

Reviewer #1

(Remarks to the Author)
The authors have done a nice job addressing my earlier suggestions and complaints.
Although the cross-mapped gap filling idea is not new, GP is clearly better than simplex/NN for interpolating. This is a substantial piece of work that will almost certainly end up being highly cited.

(Remarks on code availability)

Reviewer #2

(Remarks to the Author)
Authors have improved on existing imputation algorithms in the cases where dynamics of the system is fully known. This is slightly different from the case when the system equations are not known in full, but a coupled dependence is assumed between observed and partially observed variables. So in essence, here the idea is to learn a mapping between the full attractor of the system, and partially observed attractor of the system, and then use it for imputation. I believe this work could find application in scenarios when a well-understood system is observed but the exact states are unknown and most of the information about the current and accessible past states is limited. I am surprised to find that some parts of the paper overstate and wrongly claim that this algorithm can help understand "essential system dynamics". They should moderate this claim since at best this algorithm provides a way to find more information about the current state of the system, rather than unfolding the system dynamics or learning equations.

For example, in introduction, lines 83-94, and in lines 117-133, the authors have presented the algorithm as an alternative to exisiting "empirical" ones, such as MVCM. But they have not clarified that the domain of those algorithms by nature is unknown systems, which is very different from their case. On careful reading, I find the comparison rather ill-suited since they work with different information about the system and to compare their performance may not be informative on their suitability for a given situation. This distinction should be clearly stated early in the introduction. Also in the Discussion. For example, the line 433-435 is incomplete: "We demonstrated the imputation performance under the typical assumption that a specific variable is fully observed (e.g., the first variable), whereas all other variables within the system remain incomplete." since there is a bigger assumption that the system dynamics is known. They should state this assumption of known attractor wherever the other assumption of "specific variable is observed etc." is stated so the reader is not misled into believing this is another empirical approach based solely on observed partial data.
I am happy to see that they have moderated this claim and clarified the scope in the rest of the discussion section.
I am happy with the details of the methods and supplementary section.

(Remarks on code availability)

Open Access This Peer Review File is licensed under a Creative Commons Attribution 4.0 International License, which permits use, sharing, adaptation, distribution and reproduction in any medium or format, as long as you give appropriate credit to the original author(s) and the source, provide a link to the Creative Commons license, and indicate if changes were made.
In cases where reviewers are anonymous, credit should be given to 'Anonymous Referee' and the source.
The images or other third party material in this Peer Review File are included in the article's Creative Commons license, unless indicated otherwise in a credit line to the material. If material is not included in the article's Creative Commons license and your intended use is not permitted by statutory regulation or exceeds the permitted use, you will need to obtain permission directly from the copyright holder.
To view a copy of this license, visit https://creativecommons.org/licenses/by/4.0/

Reviewer #1 (Remarks to the Author):

In their manuscript "Dynamics-Informed Machine Learning for Recovering Extensive Missing Systems Dynamics" Wu and co-authors describe an approach to filling in gaps in time series based on time delay embedding and manifold learning. The manuscript demonstrates the algorithm in a wide range of simulated and empirical time series including atmospheric data, EEG data, and financial time series. Overall, the method appears to work quite well, the figures are very nicely done, and the paper is well-written.

Response: Thank you very much for your positive comments. We greatly appreciate your recognition that our method performs well, the figures are well presented and the manuscript is well organized. We have carefully revised the manuscript following all your constructive suggestions. The main revisions are summarized as follows:

In brief, we supplemented comprehensive experiments covering various non-random missing scenarios, including threshold-induced missingness, periodic missingness and simultaneous missingness across all time series. We further clarified the theoretical merits and experimental advantages of our method compared with state-of-the-art approaches. We additionally adopted real-world datasets from transportation systems to further verify the effectiveness of our proposed model.

For ease of reading, we provide the following notes regarding this response letter:

1) All italicized text in this response represents the content that has been added to the revised manuscript.

2) Figures marked as Fig. S will be included in the Supplemental information (SI).

3) Figures marked as Fig. SS will not be included in the formal manuscript and are provided for explanatory purposes in this letter.

The authors are primarily focused on values that are missing at random – which is a good place to start. However, there are many situations in which variables exhibit state-dependent or highly correlated missingness. For instance, values that are reported missing when the true value falls below a detection threshold are common in many disciplines. What happens when all of the variables have missing values at the same time? This happens frequently in real world data, e.g. when a satellite, camera, or other sensor is offline for repairs or maintenance, or there is a storm that prevents data

collection, etc. These practical situations do not seem to be addressed. Also, it seems like 90% missing still corresponds to hundreds of complete observations. What happens when you have fewer – say 10's?.

Response: Thanks greatly for your constructive suggestions. We fully agree with your valuable comments. Following your recommendation, we have incorporated two typical practical missing scenarios into our analysis: **threshold-induced missingness**, and **periodic missingness (see line 504-526 in updated main text)**. Furthermore, we have improved the framework of our method to effectively handle the simultaneous missingness of all variables. The details are given as follows:

1) For the threshold-induced missingness, to fully simulate this real-world scenario, we explicitly construct quantile-based threshold missing experiments, where missing values are generated when the true data values fall below fixed quantile thresholds of 5%, 10%, 15%, and 20% of the overall data distribution. We validate our framework for both model systems (e.g., 3D ecology system, 3D Lorenz system, 120D coupled Lorenz system and 120D coupled Rössler system) (Fig. S11), and real-world datasets including road occupancy rates, EEG series, exchange rates, wind speed series (Fig. S12). The results demonstrate that our framework has the potential to address threshold-induced missingness.

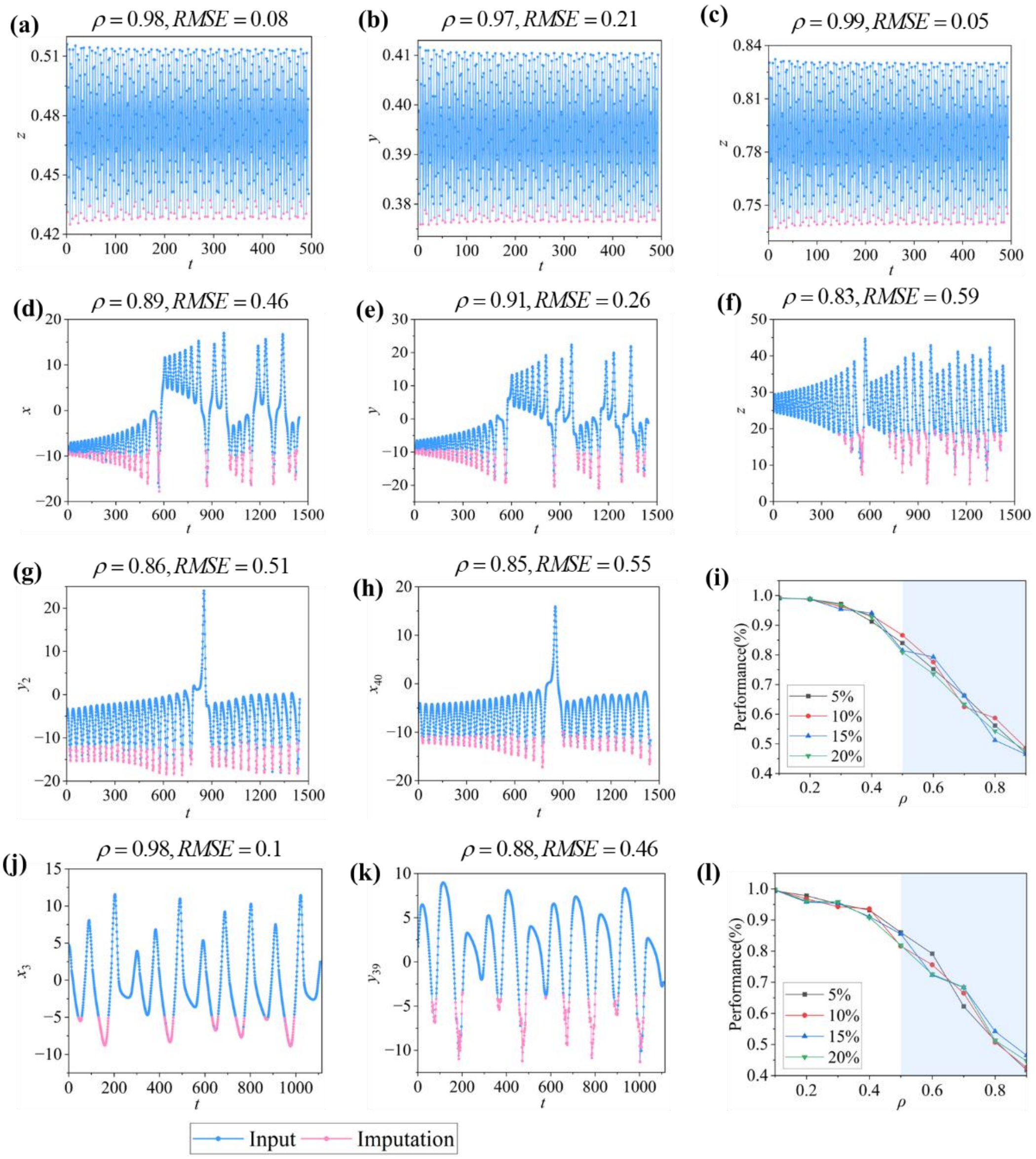

Figure S11. Imputation performance for threshold-induced missingness in model systems. (a-c) Results for the 3D ecology system, where values below the 20% quantile threshold are set as missing. (d–f) Corresponding analyses for the 3D Lorenz system. (g-i) Imputation performance for random selected observations (e.g., y_2 and x_{40}) in the 120D coupled Lorenz system. (i) Overall imputation performance in the 120D coupled Lorenz system when only one variable is fully observed while all other variables suffer from threshold-induced missingness at quantile levels of 5%, 10%, 15%, and 20%. (j-l) Corresponding analyses for the 120D coupled Rössler system.

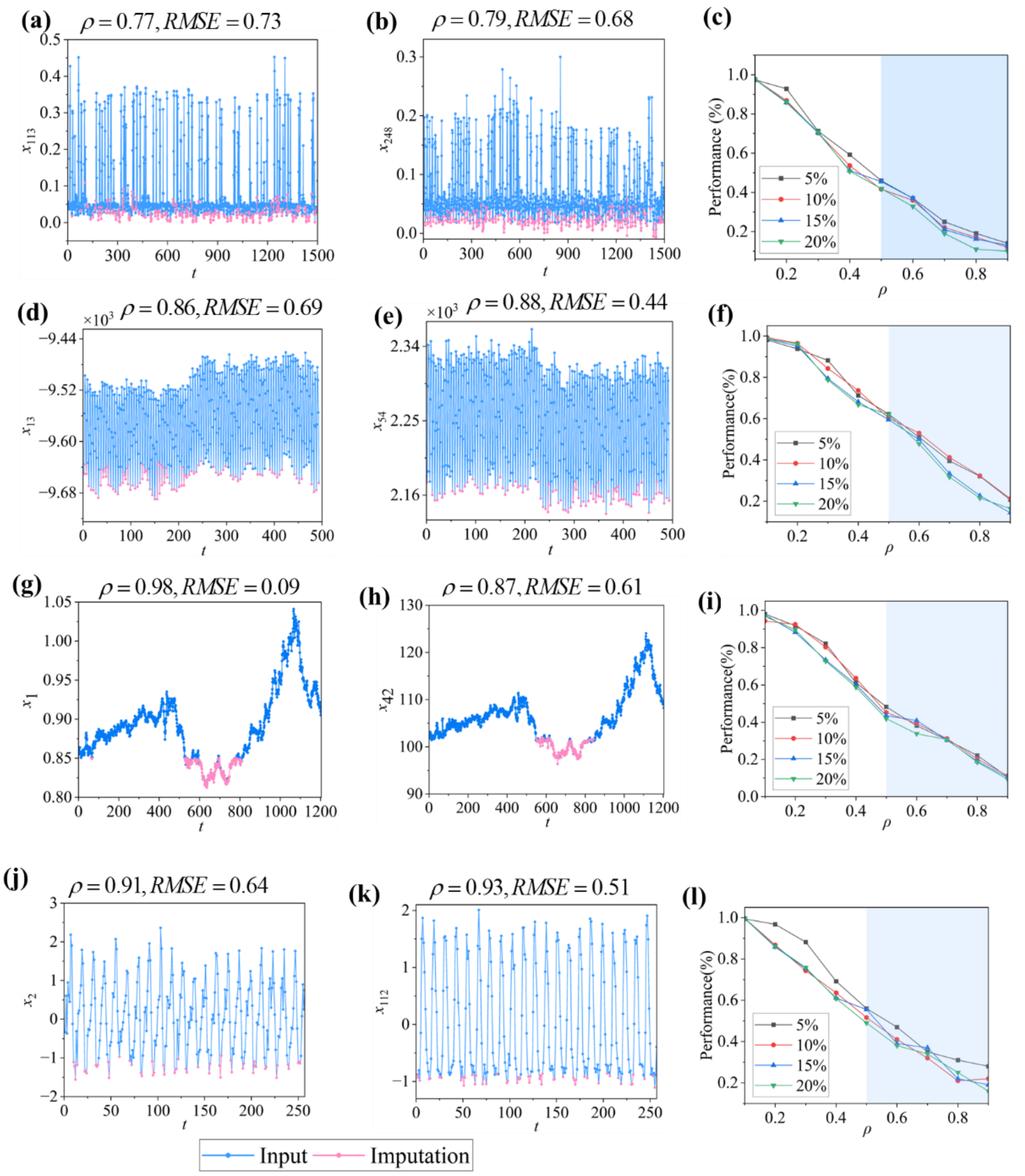

Figure S12. Imputation of threshold-induced missingness in real-world systems. (a–b) Imputation performance for road occupancy rates series. (d–f) Imputation performance for EEG series. (g–i) Imputation performance for exchange rate series. (j–l) Imputation performance for wind speed series. Note: The first two columns correspond to the scenario where data values below the 20% quantile threshold are set to missing.

2) For the periodic missingness, we conduct quantitative periodic missing experiments with periodic intervals ranging from 2 to 11 sampling steps. We also validate our framework for both model systems (e.g., 3D ecology system, 3D Lorenz system, 120D coupled Lorenz system and 120D coupled Rössler system) (Fig. S13), and real-world datasets including road occupancy rates, EEG series, exchange rates, wind speed series (Fig. S14). The results demonstrate that our framework is efficient to

address periodic missingness in complex systems.

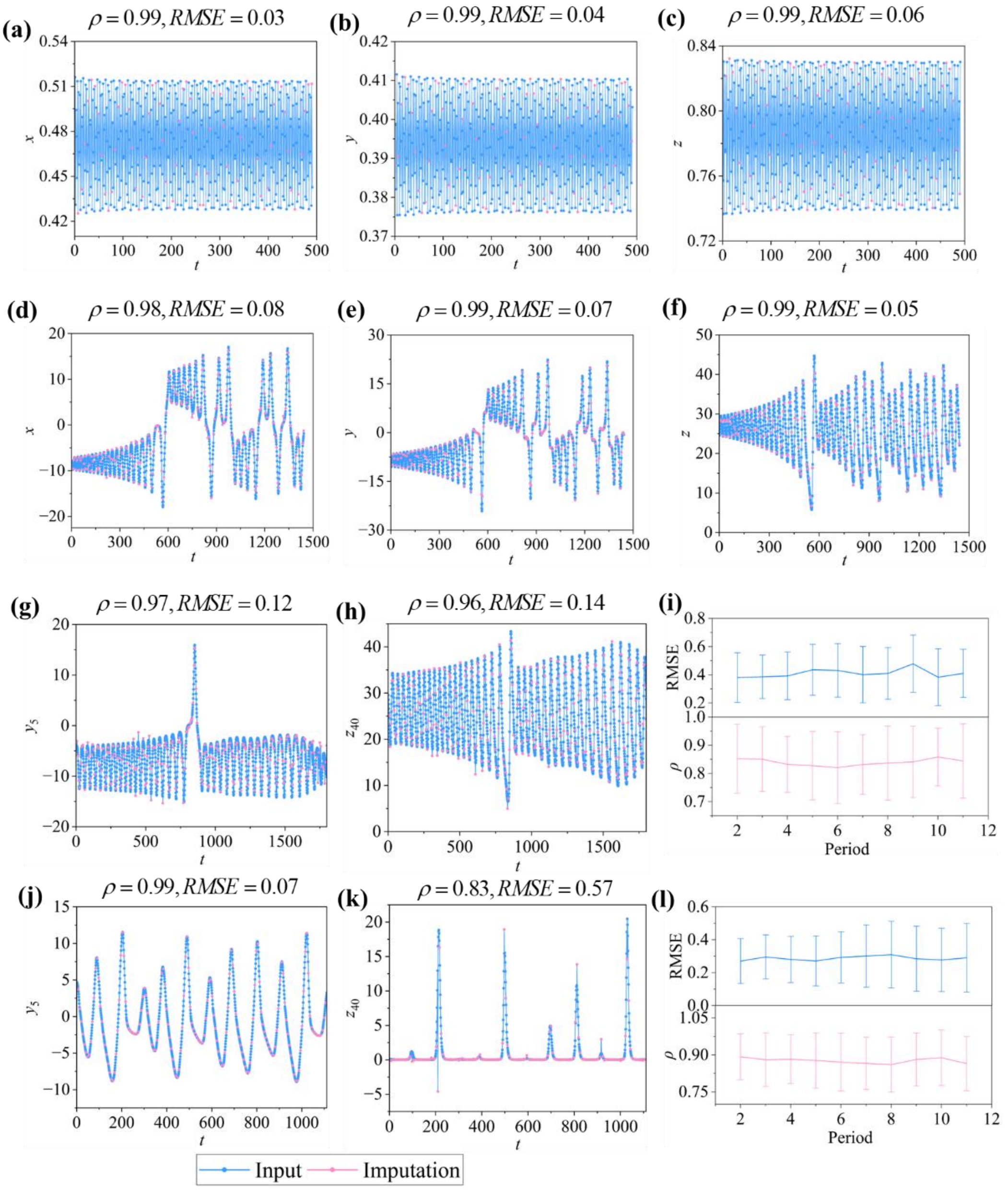

Figure S13. Imputation of periodic missingness in model systems. (a-c) Performance for the 3D ecological system, where data points are missing at a fixed interval of 7 sampling steps. (d-f) Corresponding analyses for the 3D Lorenz system. (g-i) Imputation performance for random selected observations, e.g., y_5 and z_{40} in 120D coupled Lorenz system. (i) Overall imputation performance (mean values with standard deviation error bars) for the 120D coupled Lorenz system when only one variable is fully observed while all others exhibit periodic missingness at fixed intervals ranging from 2 to 11 sampling steps. (j-l) Consistent analysis for the 120D coupled Rössler system.

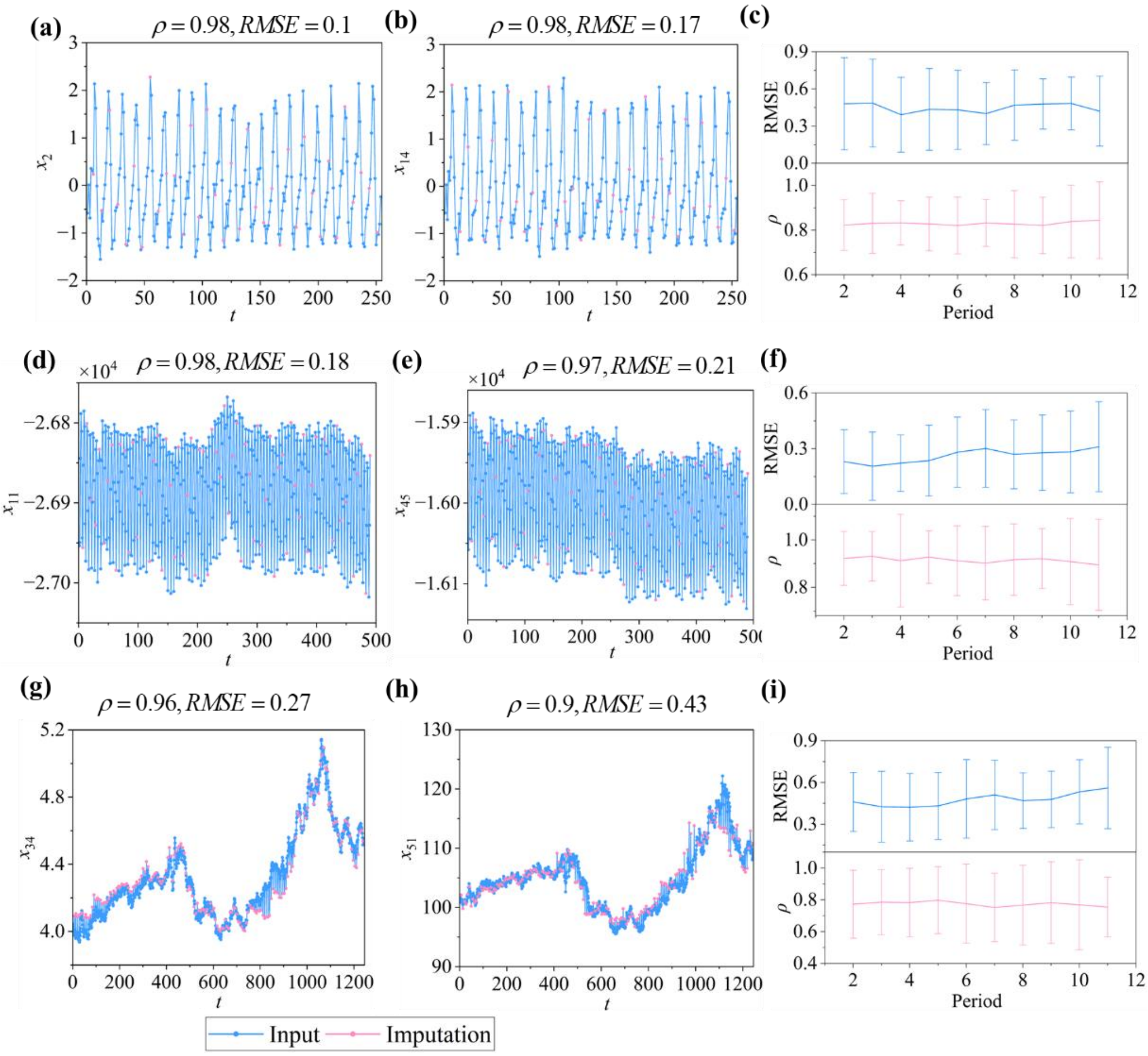

Figure S14. Imputation of periodic missingness in real-world systems. (a–c) Imputation performance for road occupancy rates. (d–f) Imputation performance for EEG series. (g–i) Imputation performance for exchange rate series. (j–l) Imputation performance for wind speed series. Note: The first two columns correspond to the periodic missing scenario with a fixed interval of 7 sampling steps.

We have summarized the discussions on threshold-induced missingness and periodic missingness in the revised main text (Lines 504–526), with the relevant content presented below.

Potential for handling non-random missingness. *In this study, missing values are typically simulated by randomly removing entries from the original time series, with missing data often distributed discretely in the series. In practical scenarios, however, missingness may follow a non-random pattern. Typical manifestations include continuous missing segments, threshold-induced missingness, and periodic missingness. Continuous missing segments correspond to prolonged consecutive data losses over a certain time interval, which widely occurs in sensor outage and device failure [57].*

Threshold-induced missingness arises when observations are missing once the underlying signal exceeds or falls below a critical threshold value. Periodic missingness refers to missing values recurring at regular intervals, which is prevalent in cyclic monitoring tasks.

We first investigate continuous missing segments under three typical scenarios: missing entries located in the first, middle, and latter halves of the time series. Experimental results across multiple datasets validate the capability of our framework to accurately impute consecutive missing elements (Fig. S10). For threshold-induced missingness, we design quantile-based missing experiments to mimic realistic non-random patterns. Missing values are generated when the original data falls below fixed quantile thresholds of 5%, 10%, 15%, and 20% of the overall distribution. Our method is capable of reliable imputation for multiple variables in such scenarios (Figs. S11 and S12). Meanwhile, its performance can be further improved in more complex situations, such as threshold-induced missing data featuring abundant abrupt data points. Finally, for periodic missingness, we perform quantitative experiments with periodic intervals ranging from 2 to 11 sampling steps. The results confirm that our framework can effectively recover periodic missing entries for complex dynamical systems (Figs. S13 and S14).

3) For the simultaneous missingness of all variables, while current frameworks necessitate at least one complete time series variable, a slight modification of our approach(i.e., lag full-partial reconstruction mapping (Fig. S15) enables it to deal with the case when all variables suffer from simultaneous missing values. As mentioned, simultaneous missingness of all variables may occur when satellites, traffic cameras, or other sensors go offline for repairs and maintenance, or when severe weather such as storms disrupts data collection. In general, the number of missing observations per variable is usually limited. This is because equipment failures or natural disturbances like storms rarely last for a long time and are typically short-lived. Accordingly, we conduct simulations under two cases, with 7 and 14 missing points per variable occurring simultaneously. We also validate our framework for both model systems (e.g., 3D ecology system, 3D Lorenz system, 120D coupled Lorenz system and 120D coupled Rössler system) (Fig. S16), and real-world datasets including road occupancy rates, EEG series, exchange rates, wind speed series (Fig. S17). The results demonstrate that our framework has potential to address simultaneous missingness of all variables. Nevertheless, there remains room for further improvement handling extensive long

continuous synchronous missing segments across all variables, as gradually accumulated dynamic prediction deviations may impair the overall imputation performance.

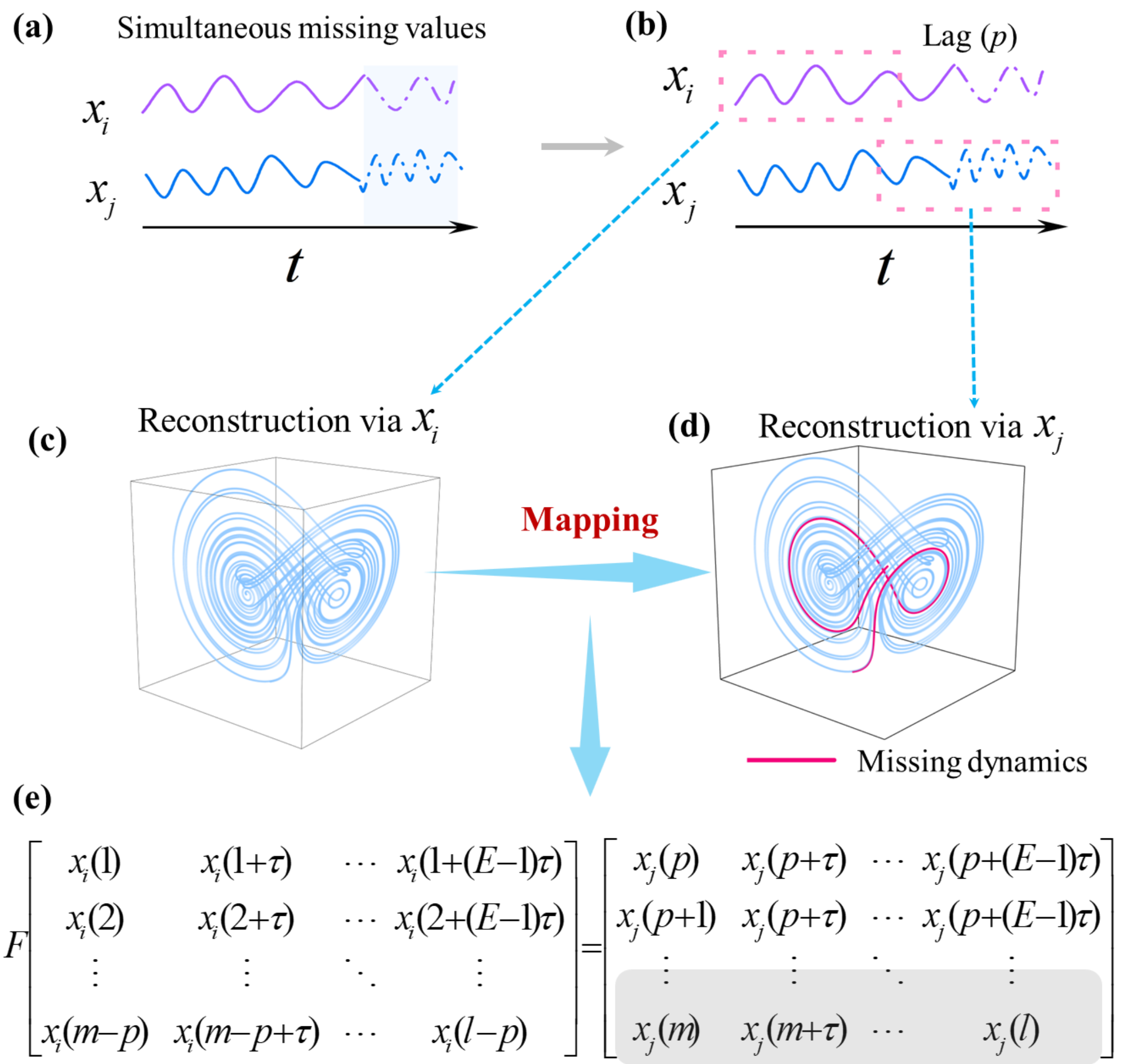

Figure S15. Imputation framework for simultaneous missingness of all variables. Suppose that all observations contain simultaneous missingness (shadow areas in (a)). We also construct full reconstruction (b, c) from complete time series and partial reconstruction from lag time series. (e) Then, we can impute missing values (shadow elements) via a one-to-one mapping from full to partial reconstruction.

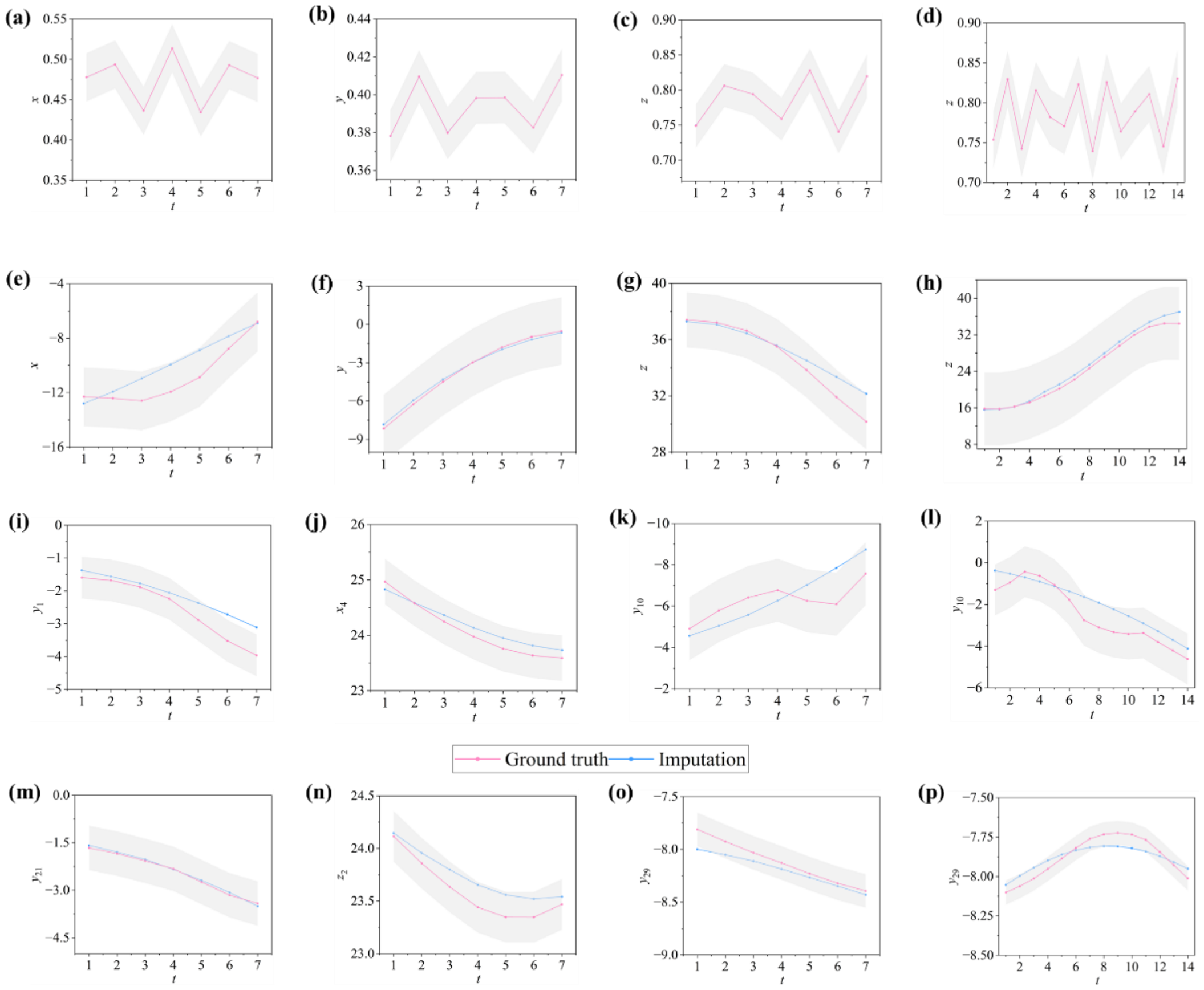

Figure S16 Imputation of simultaneous missingness of all variables in model systems. (a-d) Performance in 3D ecology system, where the number of missing points for each variable are seven (a-c) and fourteen (d). (e-h) Similar cases for the 3D Lorenz system. (i-l) Performance in the 120D coupled Lorenz system. (m-p) Similar cases for the 120D coupled Rössler system. Note: the first three and final columns simulate the numbers of missing points for each variable are seven and fourteen, respectively.

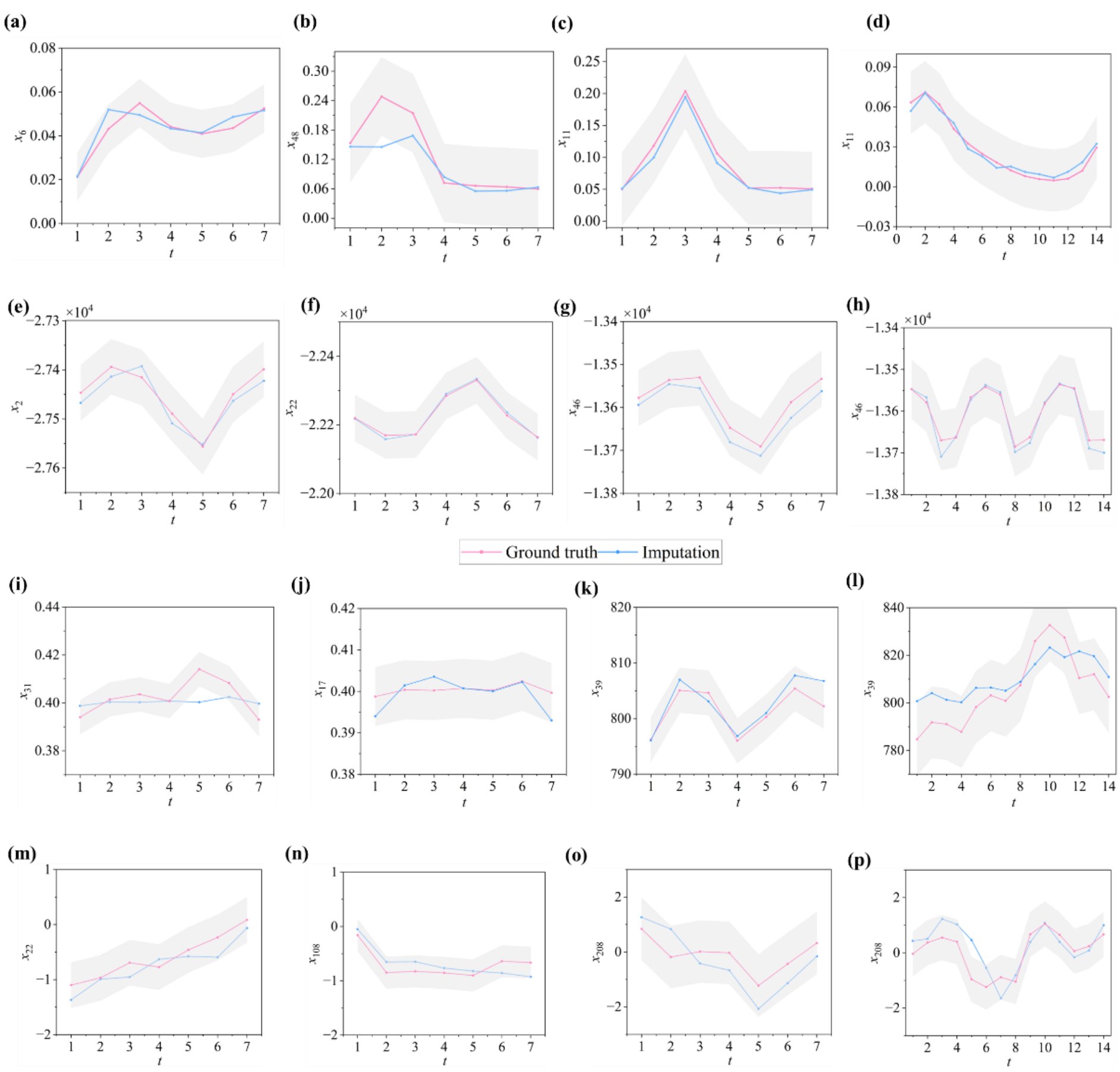

Figure S17. Imputation of simultaneous missingness of all variables in real-world systems. (a-d) Performance for road occupancy rates. (e-h) Performance for EEG series. (i-l) Performance for exchange rate series. (m-p) Performance for wind speed series. Note: the first three and final columes simulate the numbers of missing points for each variable are seven and fourteen, respectively.

We have included the discussions for the simultaneous missingness of all variables in the revised main text (Line 613-622), with the relevant content presented below.

Nevertheless, our framework can be potentially optimized to accommodate certain cases. Specifically, for scenarios where all variables suffer from simultaneous missing entries at identical time points, our framework can be extended to a lag FPRM variant. This variant employs a segment-wise cross-reconstruction strategy for synchronous missing data. It builds dynamical mappings between attractors reconstructed from staggered valid and incomplete segments of paired time series, enabling the recovery of synchronous missing entries without fully observed variables (Fig. S15). Results on multiple datasets validate the capability of our framework in handling scenarios with simultaneous missing entries across all variables (Figs. S16 and S17). Nevertheless,

there remains room for further improvement handling extensive long continuous synchronous missing segments across all variables, as gradually accumulated dynamic prediction deviations may impair the overall imputation performance.

4) For the final comment "it seems like 90% missing still corresponds to hundreds of complete observations. What happens when you have fewer – say 10's?". In this work, we primarily focus on an extreme case in which only a single variable remains completely observed, and all the remaining variables contain missing values.

The monsoon example is interesting, but makes me wish that the authors had included a more reasonable alternative model as a benchmark. As the authors are aware, data imputation is a well-established field - particularly in time series analysis. Showing that this new method works is nice and showing that it works against a few alternatives is also good. But showing that it works better than the state of the art would make it really exciting. The comparison models are all static ML – which is pretty far from the best one can do with time series data. For example, atmospheric data are typically highly autocorrelated in space and time. This suggests that a linear spatio-temporal imputation scheme – even something as simple as the Beale-Little algorithm developed to accommodate missing values in principal component analysis in the 1970's (using lags to make the comparison fair) – would suffice. I recognize that the method the authors have presented is more general than this linear imputation method, but readers, like me, typically want to know if the extra computation is worth the effort.

Response: Thanks for your important comments and suggestions. We agree that classical linear spatio-temporal imputation methods, such as the Beale–Little algorithm, perform well and are sufficient under low missing rates with abundant intact observations. However, such linear schemes rely heavily on reliable estimation of spatio-temporal correlation. When the missing proportion is extremely high and valid observations are severely limited, the estimated spatio-temporal dependence will deviate substantially from the real underlying correlation patterns, leading to unsatisfactory imputation performance in challenging high-missing scenarios. Our framework can achieve stable and accurate imputation even under extreme conditions where only one variable is fully observed and all other variables are largely missing, which cannot be well handled by conventional linear alternatives.

Following your suggestion, we have incorporated road occupancy rate series from complex transportation systems as an additional representative real-world example. We evaluate the extreme setting where only one variable remains fully observed and all

other variables are missing, and the results confirm that our method can still achieve reliable and effective imputation under this challenging scenario (Fig. 5(a-e) and Fig.6 (d, g). And then the results for wind speed series are moved to Supplementary (Fig. S8).

For comparison with existing methods, in addition to traditional statistical approaches, we have already included several dynamics-aware approaches, such as the combinations of delay embedding with support vector machine (SVM*) and delay embedding with neural network (NN*). Here, we further add two representative classical dynamics-aware methods: S-map and MetaTSI. Specifically, the S-map method reconstructs the system attractor via time-delay embedding and performs imputation through locally weighted linear regression on states in the phase space. MetaTSI achieves dynamics-aware imputation by learning continuous coordinate-based representations with meta-learning. The experimental results show that S-map fails to deliver reliable imputation performance when multivariate time series are affected by 90% missing values. Although MetaTSI achieves satisfactory results on several synthetic and real-world datasets, it exhibits limited generalization capability and performs poorly on the EEG dataset (see Table 1 in the main text). Overall, FPRM consistently achieves better imputation performance compared with the above methods.

We have incorporated the comparison with the aforementioned methods into the revised main text (Lines 401–415), and the relevant content is presented below.

Despite these static imputation methods, we further consider three representative classical dynamics-aware methods: MVCM [25], S-map [11] and MetaTSI [43]. Specifically, MVCM and the S-map method reconstruct the system attractor via time-delay embedding and perform imputation through locally weighted linear regression on states in the phase space. MetaTSI achieves dynamics-aware imputation by learning continuous coordinate-based representations with meta-learning. FPRM, MVCM and S-map are all rooted in time-delay embedding, yet they differ substantially in methodological design. MVCM and S-map both follow a local nearest-neighbor imputation paradigm: MVCM depends on multi-view attractor reconstruction and strong causal relationships across multiple variables, whereas S-map performs local estimation using only a single time series. In contrast, FPRM establishes global diffeomorphic mappings between fully and partially reconstructed attractors via Gaussian process regression. Since MVCM cannot be applied to scenarios where only one variable is fully observed while all others suffer from extensive missingness, we

instead use S-map as the comparison baseline. The results show that the FPRM clearly outperforms traditional methods across multiple datasets (see Tab. 1).

The

Table 1 Comparison with other imputation techniques

Dataset	Method										
	FPRM	Mean	*k*-NN	GPR	SVM	RF	NN	SVM*	NN*	S-map	MetaTSI
120D	(0.89,0.07)	(0.01,0.09)	(0.64,0.05)	(0.71,0.06)	(0.7,0.11)	(0.51,0.05)	(0.74,0.08)	(0.81,0.08)	(0.82,0.07)	(0.27,0.04)	(0.72,0.07)
Lorenz	(0.41,0.13)	(1.11,0.18)	(0.82,0.06)	(0.57,0.14)	(0.69,0.1)	(0.82,0.14)	(0.65,0.11)	(0.53,0.1)	(0.49,0.16)	(1.13,0.23)	(0.68,0.09)
system											
120D	(0.9,0.14)	(0.01,0.04)	(0.66,0.07)	(0.51,0.08)	(0.49,0.08)	(0.46,0.06)	(0.46,0.05)	(0.84,0.07)	(0.87,0.1)	(0.26,0.05)	(0.87,0.09)
Rossler	(0.31,0.21)	(1.07,0.15)	(0.81,0.12)	(0.67,0.11)	(0.67,0.09)	(0.79,0.11)	(0.69,0.18)	(0.44,0.09)	(0.39,0.22)	(1.12,0.17)	(0.49,0.14)
system											
500D road	(0.83,0.08)	(0.01,0.03)	(0.61,0.07)	(0.67,0.11)	(0.64,0.15)	(0.53,0.12)	(0.68,0.07)	(0.77,0.1)	(0.7,0.11)	(0.25,0.06)	(0.75,0.14)
occupancy	(0.49,0.13)	(1.01,0.07)	(0.86,0.11)	(0.68,0.16)	(0.69,0.16)	(0.89,0.13)	(0.87,0.12)	(0.65,0.14)	(0.76,0.21)	(1.22,0.39)	(0.66,0.08)
rates											
64D EEG	(0.95,0.11)	(0.01,0.02)	(0.06,0.11)	(0.66,0.1)	(0.62,0.12)	(0.68,0.09)	(0.64,0.16)	(0.91,0.09)	(0.89,0.12)	(0.22,0.06)	(0.12,0.14)
series	(0.27,0.12)	(1.07,0.12)	(1.36,0.18)	(0.71,0.16)	(0.75,0.13)	(0.85,0.17)	(0.85,0.12)	(0.38,0.13)	(0.39,0.23)	(1.32,0.28)	(1.01,0.11)
52D	(0.82,0.14)	(-0.01,0.1)	(0.71,0.12)	(0.79,0.09)	(0.79,0.12)	(0.65,0.15)	(0.78,0.1)	(0.76,0.1)	(0.78,0.15)	(0.24,0.04)	(0.86,0.01)
exchange	(0.41,0.24)	(1.03,0.09)	(0.68,0.18)	(0.43,0.15)	(0.52,0.14)	(0.77,0.21)	(0.46,0.16)	(0.48,0.21)	(0.51,0.13)	(1.3,0.27)	(0.31,0.06)
rates											

Note: For each dataset, we assume that the first variable is fully observed, whereas all other variables are incomplete, with 90% missing values. SVM* and NN* denote that the training processes of FPRM are replaced by support vector machine (SVM) and neural network (NN), respectively. The machine learning algorithms and neural networks are simulated using the Statistics and Machine Learning Toolbox in MATLAB R2024b. Each entry reports two evaluation metrics, average ρ and RMSE, as well as their standard deviations.

ecological example is a bit too trivial to count as a 'real world' application – there are only two variables, and since these exhibit cyclic dynamics, the attractor is 1-d. In the real world, the number of species vastly exceeds 2 and attractor dimensions are typically >5. I suggest simply dropping this - there's already plenty here to make a convincing case.

Response: We appreciate the important comment. We have deleted this sample and added a new representative dataset (500D road occupancy rates) from a complex transportation system.

This all said, I am having some trouble seeing how this algorithm differs- in a fundamental way - from the algorithm presented by Carpenter et al. (2025) 'Repairing gaps in ecological time series' in Methods in Ecology and Evolution. The basic idea and theoretical justification are identical. As far as I can tell, the only real difference is that Wu et al. use GP regression for state reconstruction, while Carpenter et al. use a local linear model. A trivial distinction. The authors have cited Carpenter in the context of missing variables in ecology, but have failed to note the considerable methodological overlap – or take the opportunity to highlight the differences in their approach. This seems critical if this paper is to appear in Nature.

Response: Many thanks for your valuable and insightful comment. We agree that both our method and the algorithm proposed by Carpenter et al. (2025) are built upon the time-delay embedding theorem. Nevertheless, the two approaches differ distinctly in theoretical architecture, implementation workflow, and practical applicability to complex systems, and our framework exhibits unique advantages for broad real-world complex systems. We clarify the detailed differences as follows:

(a) Theoretical architecture

The MVCM method proposed by Carpenter et al. is a local nearest-neighbor-based imputation strategy that relies on local topological similarity on the reconstructed attractor. This paradigm performs well under adequate and well-sampled attractor reconstruction. Similar to conventional nearest-neighbor-based methods, its performance strongly depends on reconstruction quality and is vulnerable to issues such as false nearest neighbors, especially when only short time series data are available and the reconstructed attractor is relatively sparse.

In contrast, our FPRM is a global dynamics-informed framework. It is established based on the diffeomorphic mapping between full and partial reconstructed attractors,

and it explicitly embeds the intrinsic dynamical priors into the mapping learning procedure. Accordingly, the imputed values naturally follow the underlying system dynamics (see Figure 3(a–c, g–i). Furthermore, FPRM directly learns the global dynamical mapping between full and partial state-space reconstructions, which circumvents the need for explicit nearest neighbor searching.

In summary, MVCM focuses on local neighbor-based topological matching, whereas FPRM learns globally consistent dynamical mappings under diffeomorphic invariance without relying on nearest neighbor search.

(b) Implementation workflow

MVCM adopts time-delay embedding to construct multi-view phase-space reconstructions from multiple correlated time series, and then achieves imputation via weighted averaging of nearest neighbor counterparts across multiple attractors. As noted in Carpenter et al., reliable multi-view reconstruction typically requires strongly causal relationships among the involved variables, which inevitably imposes additional challenges on causal identification in practical applications.

Our FPRM also employs time-delay embedding to reconstruct full and partial dynamical attractors, and then adopts Gaussian process regression to establish a robust mapping between complete and incomplete state spaces for missing value recovery. The underlying system dynamics is fully extracted from the observed time series and naturally incorporated into the learning framework. As shown in Table 1 of the main manuscript, embedding such dynamical priors into the machine learning pipeline evidently improves imputation performance. Notably, FPRM does not require explicit causal detection among multivariate time series, which offers greater flexibility in implementation.

In summary, MVCM performs imputation by multi-view nearest-neighbor averaging that requires strong causal relationships among variables, while FPRM learns a robust global dynamical mapping without relying on causal identification, making it more flexible and reliable in real-world applications.

(c) Practical implication

Our FPRM can achieve reliable imputation for multiple incomplete variables relying merely on one single fully observed variable, even under high missing ratios up to 90%. By comparison, MVCM is more suitable for scenarios with multiple simultaneously observed and causally coupled variables to support multi-view reconstruction. It is relatively less applicable to the extreme case where only one

variable is fully observed while all other variables contain extensive missingness.

Since MVCM cannot be applied to scenarios where only one variable is fully observed while all others suffer from extensive missingness, we instead use S-map as the comparison baseline (see Table 1 in the main manuscript). Both S-map and MVCM share a similar local nearest-neighbor-based imputation strategy that estimates missing values using weighted nearest-neighbor averaging in the reconstructed phase space. However, they differ in that MVCM requires multiple variables to construct multi-view attractors, while S-map performs imputation using only a single time series.

We have summarized the discussion with MVCM in the revised main text (Line 401-417), with the relevant content presented below.

Despite these static imputation methods, we further consider three representative classical dynamics-aware methods: MVCM [25], S-map [11] and MetaTSI [43]. Specifically, MVCM and the S-map method reconstruct the system attractor via time-delay embedding and perform imputation through locally weighted linear regression on states in the phase space. MetaTSI achieves dynamics-aware imputation by learning continuous coordinate-based representations with meta-learning. FPRM, MVCM and S-map are all rooted in time-delay embedding, yet they differ substantially in methodological design. MVCM and S-map both follow a local nearest-neighbor imputation paradigm: MVCM depends on multi-view attractor reconstruction and strong causal relationships across multiple variables, whereas S-map performs local estimation using only a single time series. In contrast, FPRM establishes global diffeomorphic mappings between fully and partially reconstructed attractors via Gaussian process regression. Since MVCM cannot be applied to scenarios where only one variable is fully observed while all others suffer from extensive missingness, we instead use S-map as the comparison baseline. The results show that the FPRM clearly outperforms traditional methods across multiple datasets (see Tab. 1).

Two small points by line number-

285-I am glad to see the authors move beyond the trivial Lotka-Volterra and Lorenz examples. It would be helpful in interpreting the results if-in addition to the dimensionality of the state space- the authors provided an estimate of the attractor dimension. E.g. if the attractor dimension is 1-2 as in the previous examples, then this

isn’t that much more challenging. But if it is 50 or more, then the results are quite impressive.

Response: Thanks for your valuable comment. We use the classic correlation dimension to quantify the intrinsic dimensionality of all examined systems, as this metric is a standard fractal measure for attractor characterization. As shown in Table S2, the results demonstrate that our method is validated not only on simple low-attractor-dimensional benchmarks but also on systems with higher intrinsic attractor dimensions far beyond 1–2.

Table S2 Estimation of correlation dimension in our sample

Datasets	Correlation dimension
3D ecology system	1.31
3D Lorenz system	2.12
120D coupled Lorenz system	10.97
120D coupled Rössler system	12.13
500D road occupancy rate	15.04
64D EEG series	6.24
52D exchange rate	8.93
231D wind speed	11.03

We have incorporated the aforementioned contents into the revised supplementary (Table S2).

529-530 – again the authors assert that static methods for data imputation are the only possibility, overlooking well established time series methods.

Response: Thanks for your comments.

For comparison with existing methods, in addition to traditional statistical approaches, we have already included several dynamics-aware approaches, such as the combinations of delay embedding with support vector machine (SVM*) and delay embedding with neural network (NN*). In this section, we further add two representative classical dynamics-aware methods: S-map and MetaTSI. Specifically, the S-map method reconstructs the system attractor via time-delay embedding and performs imputation through locally weighted linear regression on states in the phase space. MetaTSI achieves dynamics-aware imputation by learning continuous coordinate-based representations with meta-learning. The experimental results show

that S-map fails to deliver reliable imputation performance when multivariate time series are affected by 90% missing values. Although MetaTSI achieves satisfactory results on several synthetic and real-world datasets, it exhibits limited generalization capability and performs poorly on the EEG dataset (see Table 1 in the main text). Overall, FPRM consistently achieves superior imputation performance compared with the above methods.

Reviewer #1 (Remarks on code availability):

Some of the code are available at this link - but not enough to rerun the examples. E.g. the mlx code seems to references a function PhaSpaRecon which is not provided, similarly the file for the high dimensional Rossler example is missing. These are only two examples, not a thorough list of all of the things that are missing that would be required to repeat this analysis.

Response: Thanks for your comments. We have re-implemented the function PhaSpaRecon. We have now uploaded complete codes at the following repository: https://doi.org/10.6084/m9.figshare.30446765.

Reviewer #2 (Remarks to the Author):

The manuscript presents an imputation framework for recovering missing values in a multivariate time series data from a known system, based on knowledge of system dynamics and the availability of complete time series for at least one variable. The framework relies on Takens' embedding theorem, which provides a mathematical basis for the approach's validity in principle. The presentation of the results is clear, and the selection of test systems is diverse and appropriate. However, the framework's positioning as a general data-driven modelling approach appears somewhat overstated, given its reliance on a priori knowledge of the system dynamics. In addition, the statistical analysis would benefit from modest improvements in both presentation and depth. Overall, the proposed framework is promising and demonstrates advantages over model-agnostic imputation approaches.

Response: We sincerely thank you for your favorable evaluation. We highly appreciate your comment that this work delivers a promising and theoretically well-motivated framework, and also recognizes its superiority over model-agnostic imputation methods.

We have thoroughly revised the manuscript to fully address all your valuable comments and suggestions. The main revisions are summarized as follows:

Specifically, we have revised and clarified ambiguous expressions to avoid potential misunderstanding. Besides, we supplemented detailed parameter sensitivity analysis, optimized unclear figures and tables, and objectively discussed the limitations of our method.

For ease of reading, we provide the following notes regarding this response letter:
1) All italicized text in this response represents the content that has been added to the revised manuscript.
2) Figures marked as Fig. S are included in the Supplemental information (SI).
3) Figures marked as Fig. SS will not be included in the formal manuscript and are provided for explanatory purposes in this letter..

Specifically, I have the following comments and suggestions:

1. The introduction frames the scope as "data-driven modelling" in the early sections. e.g. lines 82-90: "the reliable reconstruction of such dynamics may be compromised when the time series contains substantial missing values..." . This framing suggests that the primary objective is the discovery of underlying system dynamics and, ultimately, improved model identification. However, the framework addresses a different problem: the recovery of missing values in observational time series for a system whose dynamics are assumed to be already known. This distinction is important and should be clarified. The authors are encouraged to revise the Introduction to more accurately reflect the scope of the work.

Response: Thanks for your important comments. We apologize for the ambiguous expression that led to misunderstanding. Our intention is to use an example to emphasize the importance of missing value imputation for the reliable reconstruction of the dynamical system structure, rather than to identify the system's underlying dynamical equations or improve model identification. In other words, we present a straightforward practical example to highlight the significance of time series imputation in complex dynamical systems by illustrating the connection between complete time series and system dynamics.

To clarify this point, we presented a specific case of the Lorenz system in the

Introduction. Specifically, if we can obtain complete time series of all variables generated by a complex system, we can use these time series to approximately reconstruct the system's geometric structure in the state space. This process is demonstrated by three time series derived from the Lorenz system (Fig. SS1(a, b)) and the corresponding state-space reconstruction (Fig. SS1(c, d)). Such reliable reconstruction will facilitate various subsequent downstream dynamical analysis tasks, such as dynamical behavior prediction. In contrast, if the time series contain missing values, the accuracy of the reconstruction will be severely compromised. The reconstructed dynamical structure will contain substantial missing information as shown in Fig. SS1(e, f), which may further hinder subsequent downstream tasks. Therefore, imputing missing values in time series is of great importance for the analysis of dynamical systems.

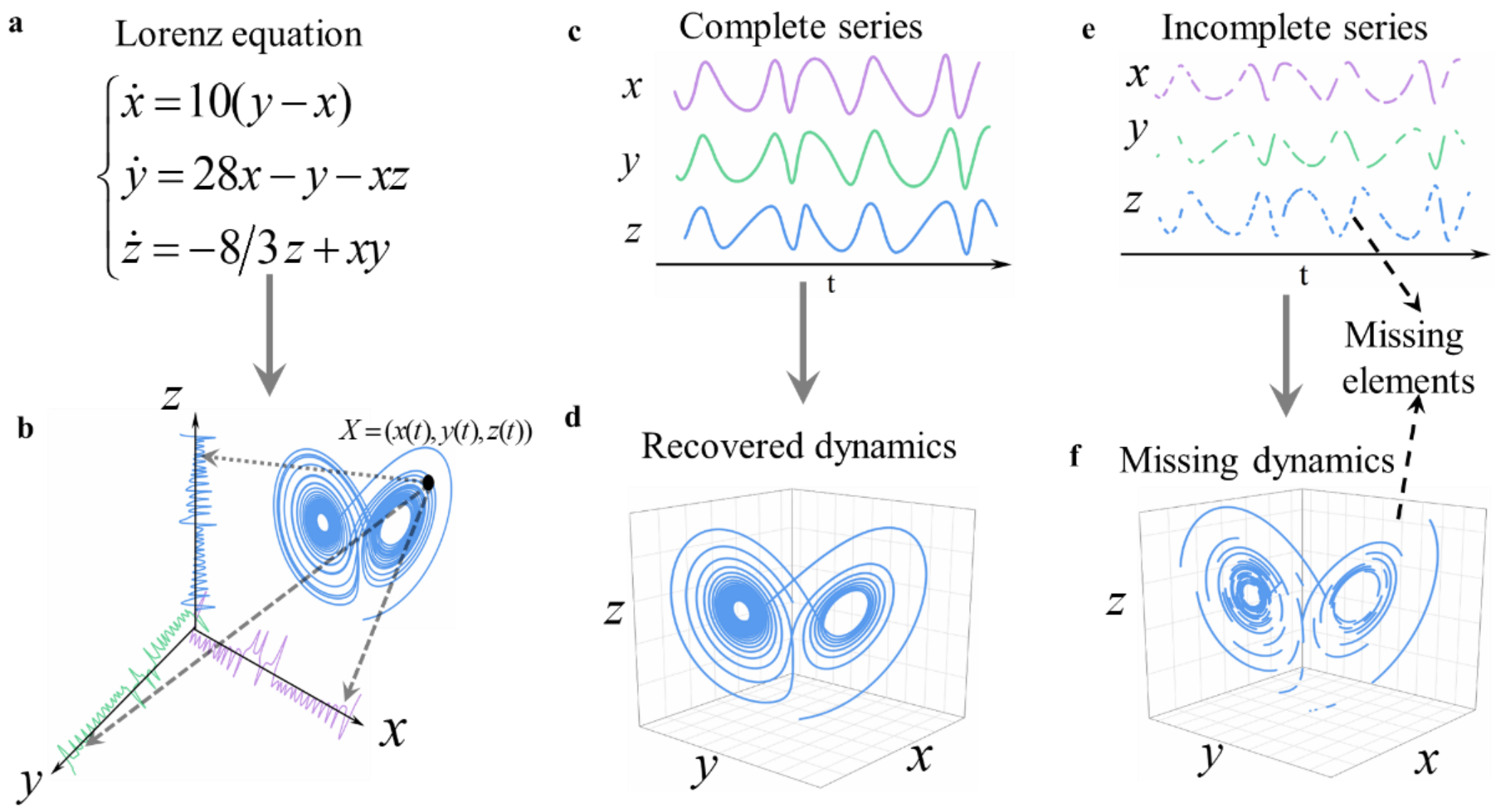

Figure SS1. Time series and system dynamics

To avoid potential misunderstanding, we have revised and clarified the relevant descriptions in the Introduction (Lines 82–94), as presented below.

Furthermore, time series observations are intrinsically linked to the underlying dynamics of complex systems. Missing values in observed time series can pose an obstacle to capturing essential system dynamics [27]. For illustration, consider the classic Lorenz system, a prototypical simplified model of fluid dynamics [28]. Its dynamics evolves over time to form a butterfly-like structure in the state space, with system components directly mapped to three time series observations (Figs. 1(d, g)). Consequently, these complete time series can inversely approximate the full dynamical

behavior of the underlying system (Figs. 1(e, h)), and such reliable reconstruction will facilitate various subsequent downstream dynamical analysis tasks (e.g., dynamical behavior prediction). However, the reliable reconstruction of such dynamics may be compromised when the time series contains substantial missing values (Figs. 1(f, i)). Therefore, imputing missing values in time series is of great importance for the analysis of dynamical systems.

2. The comparison with existing methods focuses primarily on model-agnostic or system-unaware approaches, which implicitly combine model learning and imputation (e.g., machine learning, deep learning, or Bayesian methods). While such comparisons are useful from a practical standpoint, the manuscript would benefit from discussing comparisons with other dynamics-informed or system-aware approaches, if available. This is only a suggestion.

Response: Thanks for your constructive suggestion. For comparison with existing methods, in addition to traditional statistical approaches, we have already included several dynamics-aware approaches, such as the combinations of delay embedding with support vector machine (SVM*) and delay embedding with neural network (NN*). Here we further add two representative classical dynamics-aware methods: S-map and MetaTSI. Specifically, the S-map method reconstructs the system attractor via time-delay embedding and performs imputation through locally weighted linear regression on states in the phase space. MetaTSI achieves dynamics-aware imputation by learning continuous coordinate-based representations with meta-learning. The experimental results show that S-map fails to deliver reliable imputation performance when multivariate time series are affected by 90% missing values. Although MetaTSI achieves satisfactory results on several synthetic and real-world datasets, it exhibits limited generalization capability and performs poorly on the EEG dataset (see Table 1 in the main text). Overall, FPRM consistently achieves better imputation performance compared with the above methods.

We have incorporated the comparison with the aforementioned methods into the revised main text (Lines 401–415), and the relevant content is presented below.

Despite these static imputation methods, we further consider two representative classical dynamics-aware methods: MVCM [25], S-map [11] and MetaTSI [43].

Specifically, MVCM and the S-map method reconstruct the system attractor via time-delay embedding and perform imputation through locally weighted linear regression on states in the phase space. MetaTSI achieves dynamics-aware imputation by learning continuous coordinate-based representations with meta-learning. FPRM, MVCM and S-map are all rooted in time-delay embedding, yet they differ substantially in methodological design. MVCM and S-map both follow a local nearest-neighbor imputation paradigm: MVCM depends on multi-view attractor reconstruction and strong causal relationships across multiple variables, whereas S-map performs local estimation using only a single time series. In contrast, FPRM establishes global diffeomorphic dynamical mappings between fully and partially reconstructed attractors via Gaussian process regression. Since MVCM cannot be applied to scenarios where only one variable is fully observed while all others suffer from extensive missingness, we instead use S-map as the comparison baseline. The results show that the FPRM clearly outperforms traditional methods across multiple datasets (see Tab. 1).

3. Introduction, lines 91-93: It is not clear what 'deletion' means here. While readers familiar with imputation methods understand the technical meaning, clarifying it would improve comprehension for average readers. e.g., does it mean that the entire time series is deleted for the erratic observable?

Response: Thanks for your valuable suggestion. As traditional approaches, deletion here refers to removing time points that contain missing values to achieve temporal alignment across variables, rather than discarding entire time series variables. We have added a brief explanation after "deletion" for clarification in main text (Line 95-97) , and the relevant content is presented below.

Practically, while datasets with sparse missing values may be addressed via deletion ***(e.g., time points containing missing values are removed for temporal alignment across variables)***

4. Introduction (lines 104–108): The manuscript states that real-world systems are inherently nonlinear and subject to noise and time-varying shifts, and the subsequent paragraph suggests that the proposed framework addresses all of these challenges. While the claim of addressing nonlinearity is justified, nonstationary (including time-varying shifts) is also a limitation of the proposed approach, as acknowledged later in the manuscript. This creates a degree of inconsistency in the framing. The authors may

wish to moderate this claim in the Introduction.

Response: Thank you for your careful comment. To make the presentation more rigorous, we have removed the claim regarding nonstationarity (including time-varying distributional shifts) in the Introduction (Line112-1113).

5. Results (lines 154-186): Taken's theorem forms the basis of the proposed framework. However, its application to data relies on several assumptions, such as infinite or sufficiently long time series, an appropriate embedding dimension, and noise-free observations. While these assumptions can be approximated in practice, as demonstrated in the manuscript, this still warrants discussion of the limits with respect to the required data size. Particularly, it would help to mention the constraint on the length of the available time series relative to the system timescale. This is an important practical consideration and would help clarify the method's range of applicability.

Response: Thanks for your valuable comment and suggestion. We have supplemented the corresponding sensitivity analysis on time series length in the revised supplementary material (Figure S5). Experimental results across multiple datasets show that our method exhibits stable and robust imputation performance even for short time series with only 50 data points (Fig. S5(g)). The framework maintains reliable performance as the series length varies.

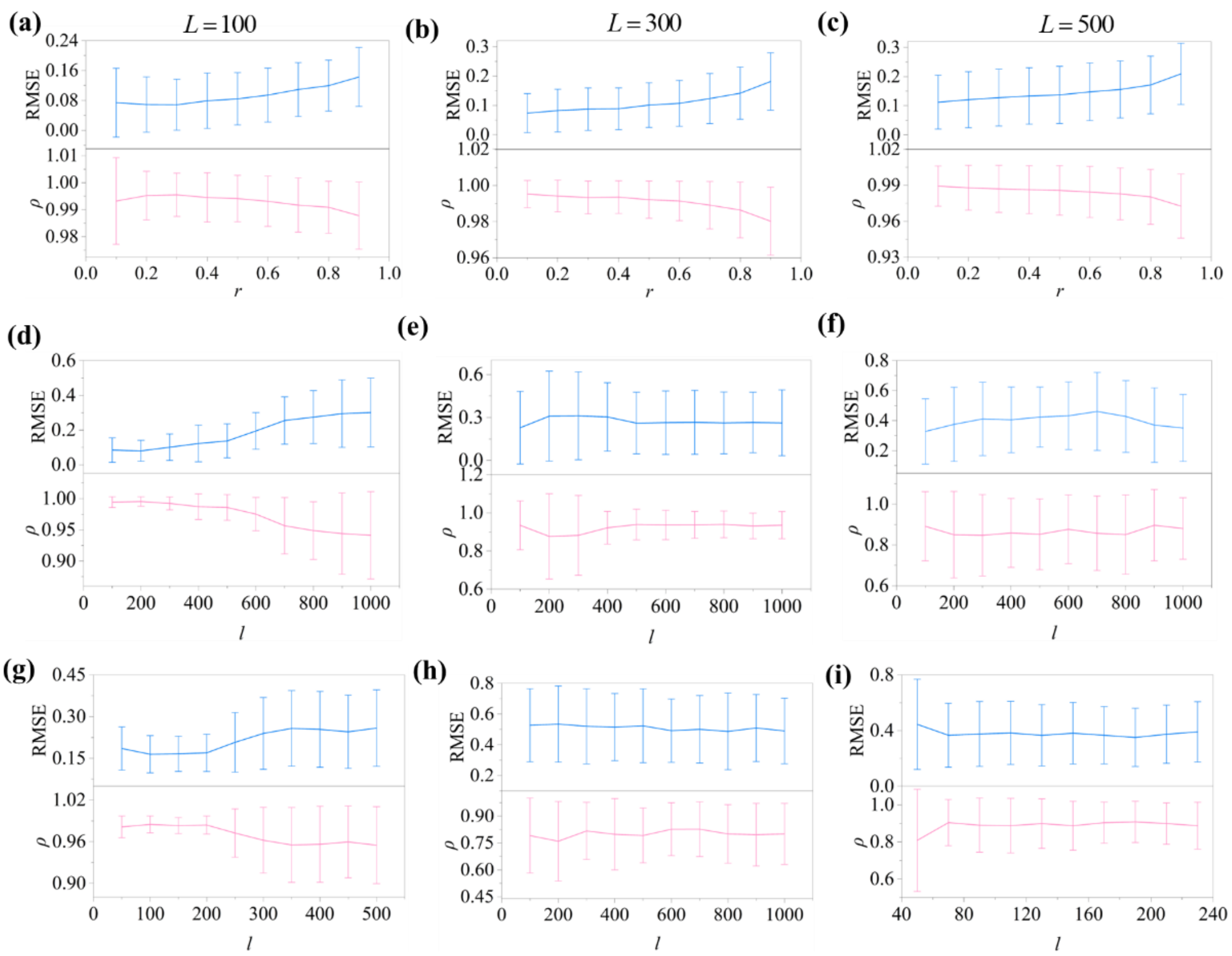

Figure S5. Robustness test of length of datasets. (a-c) Performance for the 3D Lorenz system under different time series lengths ($l = 100, 300, 500$) and missing rates ranging from $r = 0.1$ to $r = 0.9$. (d) Performance for the coupled Lorenz system as the time series length increases from $l = 100$ to $l = 1000$, with a fixed missing rate of $r = 0.5$. (e) Corresponding analyses for the coupled Rössler system. (f-i) Robustness tests on real-world datasets, including road occupancy rates, EEG signals, exchange rates, and wind speed series, consistent with the datasets used in the main manuscript. Note: In all tests, only one variable is assumed to be fully observed, while all other variables contain missing values. Error bars represent the standard deviation of the evaluation metrics across repeated trials.

We have incorporated the robustness test of length into the revised Supplementary (Fig. S5)

6. Figure 3: The manuscript presents imputation results for the variable y across different noise levels, but only for a single missing data ratio (r=0.5). For completeness, it would be helpful to include analogous results for the variable z as well as for additional missing data ratios. While these results need not all be included in the main figures, they could be incorporated into Table 1 or provided as supplementary material to give a more comprehensive evaluation.

Response: Thanks for your constructive suggestion. We have added the imputation for variable *x* and *z*, see Figs. S3 and S4 in Supplementary information.

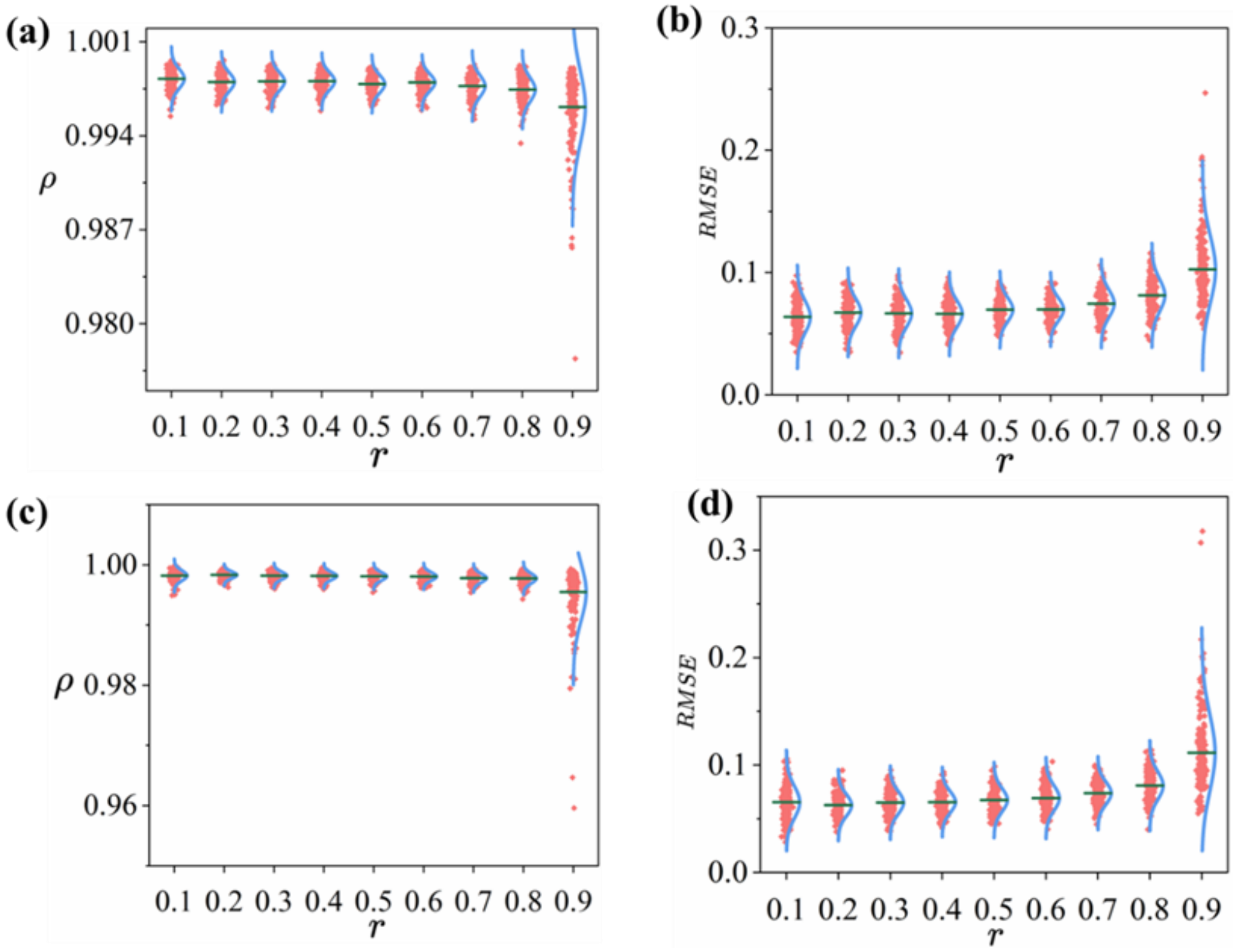

Figure S3. Imputation of incomplete series in 3-dimensional ecology system. (a, b) Imputation performance of variable x with increasing missing elements, where variable y is complete and used for the imputation. (c, d) Imputation of variable z using complete variable y. Our method achieves reliable imputation of substantial missing elements in this nonlinear system. Note: The distribution is derived from 200 repeated experiments. r represents the proportion of missing values, and ranges from $r = 0.1$ to $r = 0.9$.

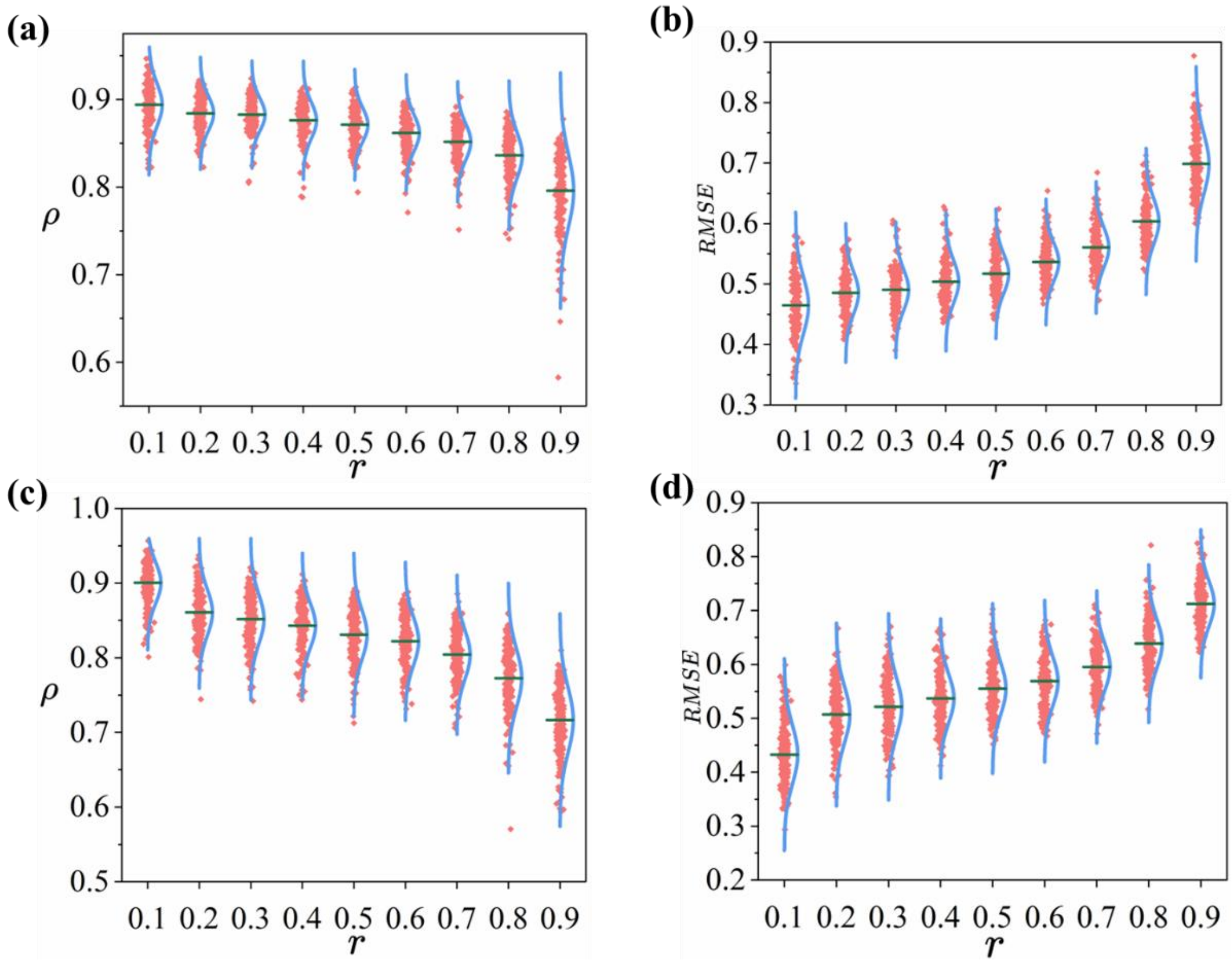

Figure S4. Imputation of incomplete series in 3-dimenisonal Lorenz system. (a, b) Imputation performance of variable x with increasing missing elements. (c, d) Performance of variable z. Note: Variable y is assumed to have complete information. The distribution is derived from 200 repeated experiments. r represents the proportion of missing values, and ranges from $r = 0.1$ to $r = 0.9$.

7. Figures 3 and 4: The Pearson correlation coefficient and RMSE appear to be plotted in a manner that suggests they follow the same distribution (this is particularly noticeable at higher noise levels, e.g., noise magnitude = 0.9). As these metrics represent fundamentally different quantities, this visualisation may be misleading. It would be preferable to present them separately or clearly distinguish them within the same figure, e.g., using different colours, as in Figure 6.

Response: Thanks for your careful suggestion. In these figures, we use kernel density curves to visualize the overall distribution of each metric (i.e., ρ and RMSE). While the plotting style is consistent, the underlying distributions for the two metrics are fundamentally different. To further improve clarity and explicitly, we have revised all these figures (Figure 3 and 4) by adding the rug plots (blue tick marks along the horizontal axis). These marks provide fine-grained information by directly displaying

the raw values from all individual trials, as illustrated in the example below.

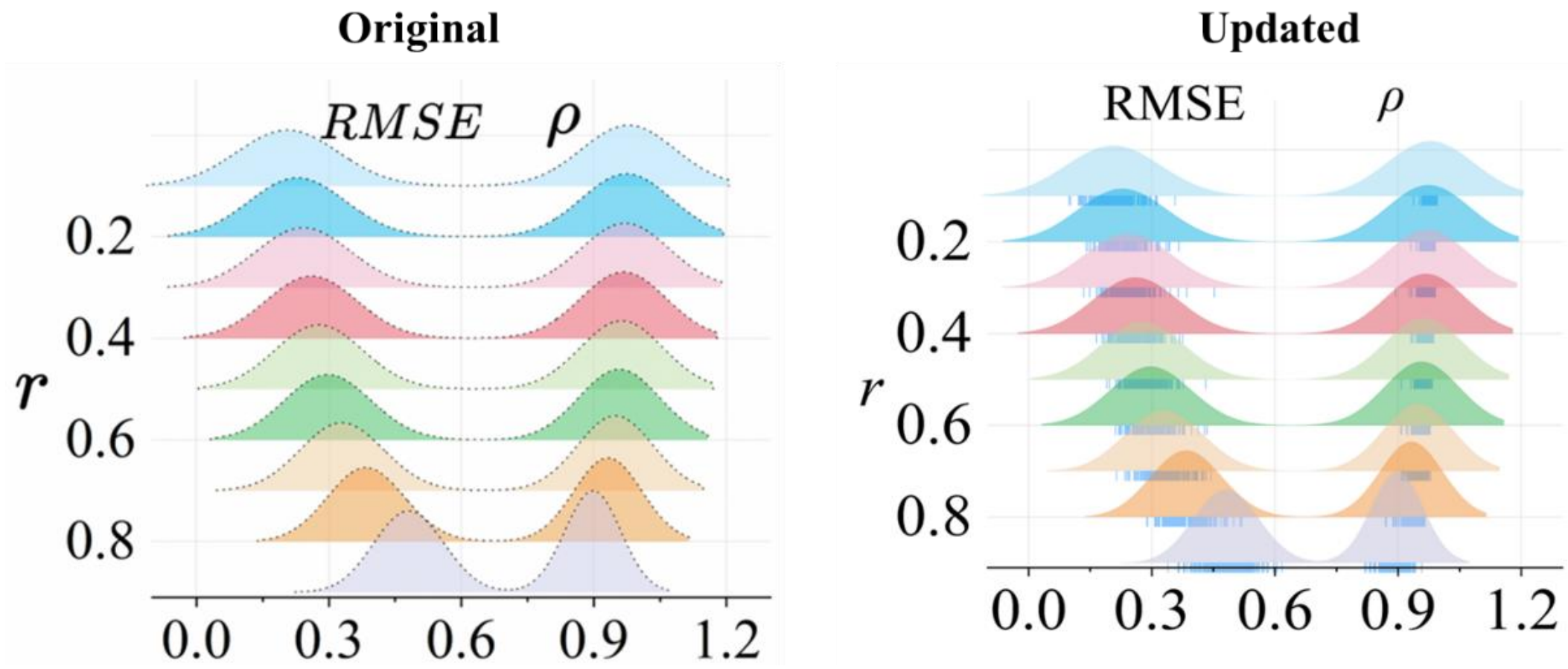

Figure SS2. Updated version of the distribution plot

We have updated all the aforementioned figures into the revised main text (Figs. 3 and 4).

8. Figure 5: Connected to the previous point, the manuscript does not appear to include an analysis for the aforementioned metrics under different noise distributions for the real-world systems. Incorporating such an analysis could strengthen the interpretation of the results, particularly in relation to Figure 6, where the performance of the proposed method appears to degrade for real-world datasets, and is attributed to inherent noise. Exploring the sensitivity to different noise characteristics would provide additional insight into how to best implement the framework for practical applications.

Response: Thanks for your important comments. In practice, the real-world datasets we use (such as EEG and exchange rates) already contain inherent noise originating from their collection environment or underlying physiological processes. Explicitly adding further synthetic noise to these data would create an artificial, non-physiological noise structure that is difficult to interpret and may not reflect natural conditions.

Instead, inspired by your suggestion, we have extended our noise sensitivity analysis on model systems to include colored noise (e.g., autoregressive noise), in addition to the original white Gaussian noise. The results confirm that our framework remains stable and reliable under both white and colored noise conditions.

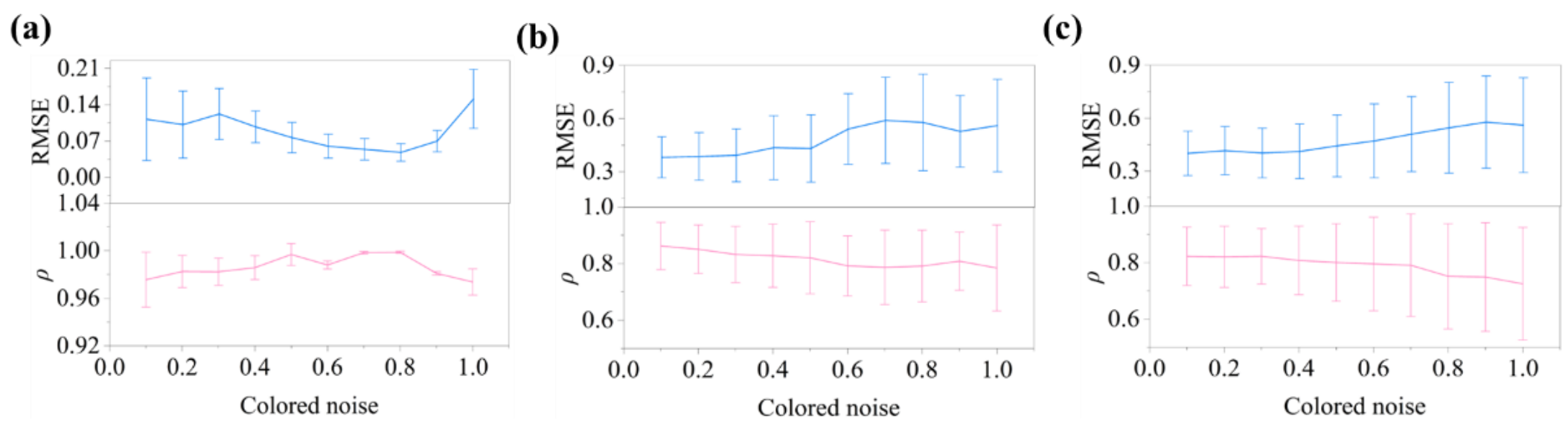

Figure S6. Robustness test under colored noise. (a) Performance for the 3D ecology system as the colored noise intensity increases from 0.1 to 1. (b) Performance for the 120D coupled Lorenz system. (c) Performance for the 120D coupled Rössler system. Note: the colored noise is simulated via a typical $AR(1)$ autoregressive model: $\eta(t)=\rho\eta(t-1)+\varepsilon(t)$, where $\varepsilon(t)$ is the white noise, and ρ is the correlation parameter (we set $\rho=0.8$ in this work).

We have included the robustness test of colored noise into the revised Supplementary (Fig. S6).

9. Table 1: The table reports performance metrics for the compared algorithms; however, it appears that only mean values are presented. For a more rigorous statistical assessment, it would be beneficial to include measures of variability, such as standard deviations or confidence intervals. This is particularly relevant given that Figure 5 suggests a substantial spread in the distributions of these metrics, which should be reflected in the summary statistics.

Response: Thanks for your constructive comments. We have now added the standard deviations for all evaluation metrics, as shown in Table 1.

10.

Table 1 Comparison with other imputation techniques

Dataset	Method										
	FPRM	Mean	k-NN	GPR	SVM	RF	NN	SVM*	NN*	S-map	MetaTSI
120D	(0.89,0.07)	(0.01,0.09)	(0.64,0.05)	(0.71,0.06)	(0.7,0.11)	(0.51,0.05)	(0.74,0.08)	(0.81,0.08)	(0.82,0.07)	(0.27,0.04)	(0.72,0.07)
Lorenz system	(0.41,0.13)	(1.11,0.18)	(0.82,0.06)	(0.57,0.14)	(0.69,0.1)	(0.82,0.14)	(0.65,0.11)	(0.53,0.1)	(0.49,0.16)	(1.13,0.23)	(0.68,0.09)
120D	(0.9,0.14)	(0.01,0.04)	(0.66,0.07)	(0.51,0.08)	(0.49,0.08)	(0.46,0.06)	(0.46,0.05)	(0.84,0.07)	(0.87,0.1)	(0.26,0.05)	(0.87,0.09)
Rossler system	(0.31,0.21)	(1.07,0.15)	(0.81,0.12)	(0.67,0.11)	(0.67,0.09)	(0.79,0.11)	(0.69,0.18)	(0.44,0.09)	(0.39,0.22)	(1.12,0.17)	(0.49,0.14)
500D road	(0.83,0.08)	(0.01,0.03)	(0.61,0.07)	(0.67,0.11)	(0.64,0.15)	(0.53,0.12)	(0.68,0.07)	(0.77,0.1)	(0.7,0.11)	(0.25,0.06)	(0.75,0.14)
occupancy rates	(0.49,0.13)	(1.01,0.07)	(0.86,0.11)	(0.68,0.16)	(0.69,0.16)	(0.89,0.13)	(0.87,0.12)	(0.65,0.14)	(0.76,0.21)	(1.22,0.39)	(0.66,0.08)
64D EEG	(0.95,0.11)	(0.01,0.02)	(0.06,0.11)	(0.66,0.1)	(0.62,0.12)	(0.68,0.09)	(0.64,0.16)	(0.91,0.09)	(0.89,0.12)	(0.22,0.06)	(0.12,0.14)
series	(0.27,0.12)	(1.07,0.12)	(1.36,0.18)	(0.71,0.16)	(0.75,0.13)	(0.85,0.17)	(0.85,0.12)	(0.38,0.13)	(0.39,0.23)	(1.32,0.28)	(1.01,0.11)
52D	(0.82,0.14)	(-0.01,0.1)	(0.71,0.12)	(0.79,0.09)	(0.79,0.12)	(0.65,0.15)	(0.78,0.1)	(0.76,0.1)	(0.78,0.15)	(0.24,0.04)	(0.86,0.01)
exchange rates	(0.41,0.24)	(1.03,0.09)	(0.68,0.18)	(0.43,0.15)	(0.52,0.14)	(0.77,0.21)	(0.46,0.16)	(0.48,0.21)	(0.51,0.13)	(1.3,0.27)	(0.31,0.06)

Note: For each dataset, we assume that the first variable is fully observed, whereas all other variables are incomplete, with 90% missing values. SVM* and NN* denote that the training processes of FPRM are replaced by support vector machine (SVM) and neural network (NN), respectively. The machine learning algorithms and neural networks are simulated using the Statistics and Machine Learning Toolbox in MATLAB R2024b. Each entry reports two evaluation metrics, average ρ and RMSE, as well as their standard deviations.

Conclusion (lines 496–504): It is not entirely clear what is meant by “random values” in this context. The manuscript primarily discusses imputation based on Takens' embedding theorem, and it is therefore unclear what baseline or alternative this statement is referring to. The authors can clarify why and how ‘random values’ can be inevitable in some alternative approaches compared.

Response: Thanks for your important comments. The relevant discussion is intended to clarify the imputability of missing values in dynamical systems. We apologize for the ambiguity caused by the term “random values” in the original text. By “random values”, we refer to completely arbitrary and unpredictable numbers that follow no dynamical laws or underlying patterns. Since such purely random values cannot be predicted or inferred from the observed time series, they are fundamentally impossible to impute.

In contrast, many real-world systems can be approximated as dynamical systems, even when their explicit governing equations are unknown. Variables in such systems are governed by inherent dynamical constraints. Missing values are therefore not arbitrary random numbers, but are determined by the system’s underlying dynamics and thus recoverable.

Taking the classic Lorenz system as an example, its dynamical evolution is visualized in Figure SS2(b). As the state points evolve, the system generates three time-series variables (Fig. SS2(c)). These variables are constrained by the underlying dynamical rules, and each value is similarly constrained rather than being arbitrary. Even when some values are unobserved, they can be recovered using these inherent rules, which is supported by the equivalent topological structure reconstructed from the time-series variables (the key foundation of our method). This dynamical regularity ensures the recoverability of missing values and forms the key rationale for imputation under Takens’ embedding theorem.

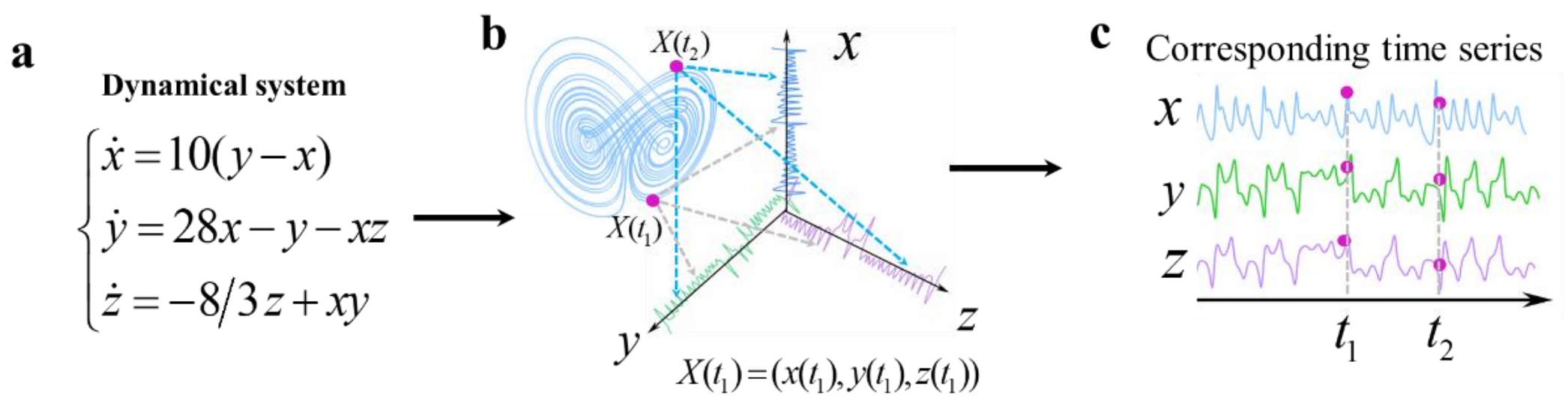

Figure SS3. Dynamical system and corresponding time series variables.

We have clarified the aforementioned methods into the revised main text (Line 542-

559)), and the relevant content is presented below.

On the other hand, Takens' theorem underscores the feasibility of imputing missing values in time series from dynamical systems. ***Generally, if missing values were purely random and unpredictable numbers with no dynamical regularity, they could not be meaningfully imputed. Importantly, many real-world systems can be approximated as dynamical systems, even when their explicit governing equations remain unknown.*** *Takens' theorem guarantees that the missing values in such a system are not* ***arbitrary random values****, as constrained by the requirement that time-series-based reconstruction must preserve the fundamental dynamics of the underlying system. Random values would lead to reconstructed attractors with drastically altered topological structures and entirely different dynamics, which directly contradicts the premises of the theorem. Consequently, missing values (except for some abrupt and unpredictable transitions such as tipping points [56]) cannot be regarded as purely random values; instead, they are governed by specific constraints that ensure that the imputed time series yields a reconstructed attractor that is diffeomorphic to the true attractor of the underlying system. This theoretical basis validates the feasibility of missing-value imputation, as well as underpins the interpretability of our FPRM framework.*

11. Conclusion (lines 510-519): The manuscript states that the proposed framework "reconstructs a topologically diffeomorphic attractor of the system in a low-dimensional space." This claim could be misinterpreted to mean that the framework offers a way to perform dimensional reduction from high-dimensional multivariate data. In the examples presented, the reconstructed attractor has the same dimensionality as the original system (or the number of variables for which data is available). In fact, the framework relies on matching the embedding dimension of the partially observed system to that of the full system in order to ensure a valid one-to-one mapping. As such, it may be more accurate to state that the method reconstructs a diffeomorphic representation of the attractor under appropriate embedding conditions, rather than implying dimensionality reduction. Clarifying this point would help avoid potential overinterpretation of the results.

Response: Thank you for this careful and constructive comment. We clarify that our framework does not perform explicit dimensionality reduction on raw high-dimensional multivariate data. Instead, under the theoretical support of Takens' delay

embedding theorem, FPRM constructs a topologically diffeomorphic attractor in an appropriately embedded low-dimensional state space. The intrinsic dynamics of a high-dimensional dissipative system can be fully represented by a low-dimensional embedded attractor, whose embedding dimension is governed by the system's inherent dynamical characteristics rather than the number of observed variables. Since the dimension of the attractor in a high-dimensional dissipative dynamical system is also much less than the dimension of the system, such an optimal embedding dimension is usually much smaller than the dimension of the original system. According to Takens' delay embedding theorem, reconstruction from time series yields a compact low-dimensional representation while completely preserving the topological invariance of the original attractor. Accordingly, delay embedding essentially provides a natural and dynamical way to achieve intrinsic dimension compression for complex systems, instead of conventional data-driven dimensionality reduction.

In this work, we adopt the false nearest neighbor method to select the optimal embedding dimension. As summarized in Table S1 of the Supplementary, the obtained embedding dimension is far lower than the dimension of the original multivariate datasets.

We have clarified related content in the revised main text (Line 560-572), with the relevant content presented below.

2) Recovering missing dynamical states in high-dimensional systems remains challenging due to complex interactions among multiple variables and widespread incomplete observations. FPRM reconstructs a topologically diffeomorphic attractor of the original system via delay embedding under proper embedding settings. High-dimensional systems generally possess redundant observational information, and their intrinsic dynamical evolution can be well characterized within a properly embedded state space [19]. The embedding dimension is determined by the inherent dynamical properties of the system rather than the number of observed variables, which is often lower than that of original dissipative high-dimensional system (i.e., $E \ll n$*, n is the dimension of the original system) [58]. Imputation is thereafter implemented within this dynamically consistent embedded space, which naturally alleviates the curse of dimensionality and lowers computational overhead, particularly for kernel function evaluation in Gaussian Process Regression (GPR).*

In summary, the manuscript presents a promising and theoretically well-motivated

framework, but its current presentation would benefit from clearer positioning, more precise articulation of assumptions, and more comprehensive presentation. Addressing the issues outlined above would substantially strengthen the manuscript and improve its overall rigour and clarity.

Reviewer #1 (Remarks to the Author):

The authors have done a nice job of responding to my earlier comments. Their efforts to address realistic patterns of missing data are commendable and results impressive. Overall, this is a very nice piece of work.

Response: Many thanks for your positive evaluation and valuable feedback. We highly appreciate your constructive suggestions, which have substantially improved this work. Following you suggestion and comments, the main revisions are summarized as follows:

First, we have clarified the concept of global diffeomorphic mapping introduced in our proposed method.

Second, we supplemented additional numerical experiments to benchmark our approach against the baseline MVCM, despite our prior comparisons covering theory, model structure and practical applications in the previous manuscript. We also added complete implementation details of MVCM and S-map to the Supplementary Information.

Note: All italicized parts are newly added in the revised manuscript.

I am however, a bit disappointed by the authors response regarding MVCM. First, the authors claim that GP regression provides a global, diffeomorphic map. Maybe there is a sense in which a GP regression is 'global,' though this is rather at odds with O'Hagan's original 1978 derivation of GP regression as a Bayesian version of local linear regression. And unless the authors have implemented some incredibly clever non-standard prior specification (which they have not reported) there is no reason at all to imagine that the output of a GP regression is a diffeomorphism. To see that this is true, simply let the length scale go to zero to find that the regression collapses to a set of delta functions evaluated at the data points: Definitely not invertible.

Response: Many thanks for your constructive comments. We fully agree with your point regarding the misrepresentation of Gaussian process regression as a "global diffeomorphic map".

First, our original intention for the word "global" was merely to contrast FPRM's manifold-wide training scheme against the pointwise local neighbour fitting of S-map and MVCM, not to claim GPR possesses global or diffeomorphic mathematical properties. Sorry for the misleading wording.

Second, the diffeomorphic correspondence exists inherently between full and

partial attractor manifolds as a direct consequence of Takens' delay embedding theorem; this topological property is intrinsic to the reconstructed state spaces and not constructed or enforced by GPR. As you mentioned, we acknowledge that standard squared-exponential kernel GPR, as implemented here, does not itself yield an invertible diffeomorphism. The GPR only approximates the pre-existing topological relation between paired attractor states for imputation, without imposing bijectivity or invertibility on the fitted function.

To eliminate potential misinterpretation, we have fully removed all instances of the term "global" from the revised manuscript. We additionally clarify that Gaussian process regression (GPR) merely serves as a flexible nonparametric proxy for reconstructing missing dynamical states. The revised text (Lines 134–154 of the Introduction) is presented below:

To address these challenges, we draw core methodological inspiration from dynamics-aware time-delay embedding frameworks including S-map [11] and MVCM [25]. S-map achieves dynamic imputation via weighted local linear approximation restricted to neighbours within a single attractor embedding view [11], while MVCM extends S-map by ensembling predictions from multiple dynamically relevant attractor views weighted by cross-mapping skill [25]. Both methods deliver competitive imputation performance for ecological time series. Distinct from the pointwise local-neighbour fitting paradigm of S-map and MVCM, our FPRM leverages the intrinsic diffeomorphic topological correspondence between attractor manifolds guaranteed by Takens' embedding theorem, and approximates this native manifold-to-manifold relation via Gaussian process regression (GPR) [46]. Specifically, FPRM first reconstructs attractor embeddings to encode system dynamics based on the delay embedding theorem [45]: a full-state reconstruction from fully observed time series, and a partial-state reconstruction from incomplete observations (Figs. 1(k, l)). We then train the GPR model on paired valid state samples spanning the entire domain of both manifolds to approximate their inherent topological mapping. This pipeline enables theoretically grounded recovery of missing dynamical trajectories by capitalizing on topological invariants retained during delay-coordinate embedding. Crucially, the diffeomorphism is an inherent topological property of the paired reconstructed manifolds, rather than a characteristic imposed by the GPR fit. The GPR merely acts as a flexible nonparametric surrogate to retrieve unobserved states based on this pre-existing manifold structure (see Materials and methods).

Second, the authors claim without evidence that MVCM is inappropriate for context in which they are working. Given the description of both algorithms, I genuinely can't see why this would be the case. But the right way to respond would have been to simply run MVCM on the simulated data and show that it does not work. Instead, the authors have chosen to run S-map as a straw man (without providing any details on how this was done), which unsurprisingly performs rather poorly.

Response: Thanks for your comments and suggestions. Following your suggestion, we add extra comparative simulation experiments of MVCM and our FPRM method (see Table 1 in main text). (Note that we have systematically benchmarked FPRM against MVCM from theoretical, structural and practical aspects in the prior version). We also document the core workflows of S-map and MVCM in the supplementary note.

Table 1 Comparison with other imputation techniques

Dataset	Method											
	FPRM	Mean	*k*-NN	GPR	SVM	RF	NN	SVM*	NN*	MVCM	S-map	MetaTSI
120D Lorenz system	(0.89,0.07)	(0.01,0.09)	(0.64,0.05)	(0.71,0.06)	(0.7,0.11)	(0.51,0.05)	(0.74,0.08)	(0.81,0.08)	(0.82,0.07)	(0.63,0.1)	(0.27,0.04)	(0.72,0.07)
	(0.41,0.13)	(1.11,0.18)	(0.82,0.06)	(0.57,0.14)	(0.69,0.1)	(0.82,0.14)	(0.65,0.11)	(0.53,0.1)	(0.49,0.16)	(0.67,0.09)	(1.13,0.23)	(0.68,0.09)
120D Rossler system	(0.9,0.14)	(0.01,0.04)	(0.66,0.07)	(0.51,0.08)	(0.49,0.08)	(0.46,0.06)	(0.46,0.05)	(0.84,0.07)	(0.87,0.1)	(0.64,0.12)	(0.26,0.05)	(0.87,0.09)
	(0.31,0.21)	(1.07,0.15)	(0.81,0.12)	(0.67,0.11)	(0.67,0.09)	(0.79,0.11)	(0.69,0.18)	(0.44,0.09)	(0.39,0.22)	(0.68,0.08)	(1.12,0.17)	(0.49,0.14)
500D road occupancy rates	(0.83,0.08)	(0.01,0.03)	(0.61,0.07)	(0.67,0.11)	(0.64,0.15)	(0.53,0.12)	(0.68,0.07)	(0.77,0.1)	(0.7,0.11)	(0.6,0.11)	(0.25,0.06)	(0.75,0.14)
	(0.49,0.13)	(1.01,0.07)	(0.86,0.11)	(0.68,0.16)	(0.69,0.16)	(0.89,0.13)	(0.87,0.12)	(0.65,0.14)	(0.76,0.21)	(0.66,0.1)	(1.22,0.39)	(0.66,0.08)
64D EEG series	(0.95,0.11)	(0.01,0.02)	(0.06,0.11)	(0.66,0.1)	(0.62,0.12)	(0.68,0.09)	(0.64,0.16)	(0.91,0.09)	(0.89,0.12)	(0.58,0.16)	(0.22,0.06)	(0.12,0.14)
	(0.27,0.12)	(1.07,0.12)	(1.36,0.18)	(0.71,0.16)	(0.75,0.13)	(0.85,0.17)	(0.85,0.12)	(0.38,0.13)	(0.39,0.23)	(0.71,0.13)	(1.32,0.28)	(1.01,0.11)
52D exchange rates	(0.82,0.14)	(-0.01,0.1)	(0.71,0.12)	(0.79,0.09)	(0.79,0.12)	(0.65,0.15)	(0.78,0.1)	(0.76,0.1)	(0.78,0.15)	(0.51,0.14)	(0.24,0.04)	(0.86,0.01)
	(0.41,0.24)	(1.03,0.09)	(0.68,0.18)	(0.43,0.15)	(0.52,0.14)	(0.77,0.21)	(0.46,0.16)	(0.48,0.21)	(0.51,0.13)	(0.79,0.18)	(1.3,0.27)	(0.31,0.06)

Note: For each dataset, we assume that the first variable is fully observed, whereas all other variables are incomplete, with 90% missing values. SVM* and NN* denote that the training processes of FPRM are replaced by support vector machine (SVM) and neural network (NN), respectively. The machine learning algorithms and neural networks are simulated using the Statistics and Machine Learning Toolbox in MATLAB R2024b. Each entry reports two evaluation metrics, average ρ and RMSE, as well as their standard deviations.

Supplementary notes

1 Main imputation pipeline of the S-map algorithm

In our benchmark setting where only one anchor time series is fully observed and all remaining variables suffer 90% data missingness, S-map reconstructs attractors solely from this complete reference and imputes missing entries of coupled incomplete series via exponential-weighted local linear regression over all valid attractor states. Specifically, we first perform state-space reconstruction with fixed embedding dimension E and time delay τ *whose values are kept identical to those adopted in the FPRM framework (Table S1). During embedding construction, we only utilize the single fully observed time series to build delay-coordinate attractors, and discard any state vectors that contain lagged values from incomplete variables to form the valid training library. The only tunable hyperparameter of S-map is the locality weight* θ*, which we searched over* $\{1,5,9,15,25,35,45\}$ *following the standard benchmark range defined in the original S-map algorithm and rEDM toolbox (https://github.com/SugiharaLab/rEDM). The model assigns exponential weights* $w(d) = \exp(-\theta d / \bar{d})$ *to valid library states based on the Euclidean state-space distance. For each time point with missing entries, we build a query attractor state solely from lagged values of the complete series. A weighted local linear regression is fitted on all valid library points, and the optimal* θ *is selected to maximize the Pearson correlation between ground truth and imputed values.*

2 Main imputation pipeline of the MVCM algorithm

In our benchmark setting where only one anchor time series is fully observed and all remaining variables suffer 90% data missingness, MVCM extends the single-view S-map paradigm and achieves imputation by ensembling predictions from multiple attractor views built upon the sole complete reference series. we first perform state-space reconstruction with fixed embedding dimension E and time delay τ *whose values are kept identical to those adopted in the FPRM framework (Table S1). For the multi-view construction, we sample distinct lag combination for the candidate lag rang [1, 20] to generate diverse attractor views, and eliminate any candidate view containing lagged values from incomplete variables. The core tunable hyperparameters of MVCM include the locality weight* θ *(consistent with S-map) and the number of top-*

performing view n. Each view produces independent S-map predictions with exponential distance weighting, and outputs are aggregated via weights proportional to each view's cross-mapping predictive skill. For each time point with missing entries, we build a query attractor state solely from lagged values of the complete series. A weighted local linear regression is fitted on all valid library points, and the optimal θ *is selected to maximize the Pearson correlation between ground truth and imputed values. For each timestamp with missing measurements, we filter valid lag-based attractor views derived from the complete anchor series, run S-map local regression for each qualified view, and compute the weighted ensemble average across top-n views. The optimal combination of* θ *and n is selected to maximize the Pearson correlation coefficient between ground-truth observations and ensemble imputed predictions.*

To alleviate any ambiguity: the only reasonable response is to acknowledge that Carpenter and Sugihara already had this idea, but applied it to a very narrow range of systems - right from the beginning, not buried in the Methods. They would then include Carpenter and Sugihara's algorithm alongside their own in the simulation testing so that readers had a clear sense of how significant a contribution was being made.

Response: Thanks for your constructive comments and suggestions.

First, following your advice, we explicitly acknowledge the foundational contributions of Carpenter and Sugihara in the Introduction (Lines 107–117), rather than placing these discussions solely within the Methods section. We further elaborate on the core innovations of our FPRM in the subsequent paragraphs of the Introduction (Lines 135–155).

Line 107-117 in the revised Introduction:

To further refine filling accuracy, researchers shift modeling paradigms to encode the intrinsic dynamics behind time series. Specifically, S-map and its multi-view extension MVCM [11,25] are classic empirical dynamic modelling tools that achieve credible gap-filling results for ecological time series with mild missing entries. Meanwhile, the meta-learning framework MetaTSI [43] provides a specialized dynamics-aware imputation pipeline to characterize intricate spatiotemporal evolution in urban traffic data. Though these methods yield satisfying performance within their respective domain boundaries, accurately recovering missing dynamics of complex systems under

high missing rates remains an open area for improvement.

Line 135-155 in the revised Introduction:

To address these challenges, we draw core methodological inspiration from dynamics-aware time-delay embedding frameworks including S-map [11] and MVCM [25]. S-map achieves dynamic imputation via weighted local linear approximation restricted to neighbours within a single attractor embedding view [11], while MVCM extends S-map by ensembling predictions from multiple dynamically relevant attractor views weighted by cross-mapping skill [25]. Both methods deliver competitive imputation performance for ecological time series. Distinct from the pointwise local-neighbour fitting paradigm of S-map and MVCM, our FPRM leverages the intrinsic diffeomorphic topological correspondence between attractor manifolds guaranteed by Takens' embedding theorem, and approximates this native manifold-to-manifold relation via Gaussian process regression (GPR) [46]. Specifically, FPRM first reconstructs attractor embeddings to encode system dynamics based on the delay embedding theorem [45]: a full-state reconstruction from fully observed time series, and a partial-state reconstruction from incomplete observations (Figs. 1(k, l)). We then train the GPR model on paired valid state samples spanning the entire domain of both manifolds to approximate their inherent topological mapping. This pipeline enables theoretically grounded recovery of missing dynamical trajectories by capitalizing on topological invariants retained during delay-coordinate embedding. Crucially, the diffeomorphism is an inherent topological property of the paired reconstructed manifolds, rather than a characteristic imposed by the GPR fit. The GPR merely acts as a flexible nonparametric surrogate to retrieve unobserved states based on this pre-existing manifold structure (see Materials and methods).

Second, we add extra comparative simulation experiments of MVCM and our FPRM method (see Table 1 in main text). We also document the core workflows of S-map and MVCM in the supplementary notes.

Table 1 Comparison with other imputation techniques

Dataset	Method											
	FPRM	Mean	*k*-NN	GPR	SVM	RF	NN	SVM*	NN*	MVCM	S-map	MetaTSI
120D	(0.89,0.07)	(0.01,0.09)	(0.64,0.05)	(0.71,0.06)	(0.7,0.11)	(0.51,0.05)	(0.74,0.08)	(0.81,0.08)	(0.82,0.07)	(0.63,0.1)	(0.27,0.04)	(0.72,0.07)
Lorenz system	(0.41,0.13)	(1.11,0.18)	(0.82,0.06)	(0.57,0.14)	(0.69,0.1)	(0.82,0.14)	(0.65,0.11)	(0.53,0.1)	(0.49,0.16)	(0.67,0.09)	(1.13,0.23)	(0.68,0.09)
120D	(0.9,0.14)	(0.01,0.04)	(0.66,0.07)	(0.51,0.08)	(0.49,0.08)	(0.46,0.06)	(0.46,0.05)	(0.84,0.07)	(0.87,0.1)	(0.64,0.12)	(0.26,0.05)	(0.87,0.09)
Rossler system	(0.31,0.21)	(1.07,0.15)	(0.81,0.12)	(0.67,0.11)	(0.67,0.09)	(0.79,0.11)	(0.69,0.18)	(0.44,0.09)	(0.39,0.22)	(0.68,0.08)	(1.12,0.17)	(0.49,0.14)
500D road	(0.83,0.08)	(0.01,0.03)	(0.61,0.07)	(0.67,0.11)	(0.64,0.15)	(0.53,0.12)	(0.68,0.07)	(0.77,0.1)	(0.7,0.11)	(0.6,0.11)	(0.25,0.06)	(0.75,0.14)
occupancy rates	(0.49,0.13)	(1.01,0.07)	(0.86,0.11)	(0.68,0.16)	(0.69,0.16)	(0.89,0.13)	(0.87,0.12)	(0.65,0.14)	(0.76,0.21)	(0.66,0.1)	(1.22,0.39)	(0.66,0.08)
64D EEG	(0.95,0.11)	(0.01,0.02)	(0.06,0.11)	(0.66,0.1)	(0.62,0.12)	(0.68,0.09)	(0.64,0.16)	(0.91,0.09)	(0.89,0.12)	(0.58,0.16)	(0.22,0.06)	(0.12,0.14)
series	(0.27,0.12)	(1.07,0.12)	(1.36,0.18)	(0.71,0.16)	(0.75,0.13)	(0.85,0.17)	(0.85,0.12)	(0.38,0.13)	(0.39,0.23)	(0.71,0.13)	(1.32,0.28)	(1.01,0.11)
52D	(0.82,0.14)	(-0.01,0.1)	(0.71,0.12)	(0.79,0.09)	(0.79,0.12)	(0.65,0.15)	(0.78,0.1)	(0.76,0.1)	(0.78,0.15)	(0.51,0.14)	(0.24,0.04)	(0.86,0.01)
exchange rates	(0.41,0.24)	(1.03,0.09)	(0.68,0.18)	(0.43,0.15)	(0.52,0.14)	(0.77,0.21)	(0.46,0.16)	(0.48,0.21)	(0.51,0.13)	(0.79,0.18)	(1.3,0.27)	(0.31,0.06)

Note: For each dataset, we assume that the first variable is fully observed, whereas all other variables are incomplete, with 90% missing values. SVM* and NN* denote that the training processes of FPRM are replaced by support vector machine (SVM) and neural network (NN), respectively. The machine learning algorithms and neural networks are simulated using the Statistics and Machine Learning Toolbox in MATLAB R2024b. Each entry reports two evaluation metrics, average ρ and RMSE, as well as their standard deviations.

Supplementary notes

1 Main imputation pipeline of the S-map algorithm

In our benchmark setting where only one anchor time series is fully observed and all remaining variables suffer 90% data missingness, S-map reconstructs attractors solely from this complete reference and imputes missing entries of coupled incomplete series via exponential-weighted local linear regression over all valid attractor states. Specifically, we first perform state-space reconstruction with fixed embedding dimension E and time delay τ *whose values are kept identical to those adopted in the FPRM framework (Table S1). During embedding construction, we only utilize the single fully observed time series to build delay-coordinate attractors, and discard any state vectors that contain lagged values from incomplete variables to form the valid training library. The only tunable hyperparameter of S-map is the locality weight* θ*, which we searched over* $\{1,5,9,15,25,35,45\}$ *following the standard benchmark range defined in the original S-map algorithm and rEDM toolbox (https://github.com/SugiharaLab/rEDM). The model assigns exponential weights* $w(d)=\exp(-\theta d/\bar{d})$ *to valid library states based on the Euclidean state-space distance. For each time point with missing entries, we build a query attractor state solely from lagged values of the complete series. A weighted local linear regression is fitted on all valid library points, and the optimal* θ *is selected to maximize the Pearson correlation between ground truth and imputed values.*

2 Main imputation pipeline of the MVCM algorithm

In our benchmark setting where only one time series is fully observed and all remaining variables suffer 90% data missingness, MVCM extends the single-view S-map paradigm and achieves imputation by ensembling predictions from multiple attractor views built upon the sole complete reference series. we first perform state-space reconstruction with fixed embedding dimension E and time delay τ *whose values are kept identical to those adopted in the FPRM framework (Table S1). For the multi-view construction, we sample distinct lag combination for the candidate lag rang [1, 20] to generate diverse attractor views, and eliminate any candidate view containing lagged values from incomplete variables. The core tunable hyperparameters of MVCM include the locality weight* θ *(consistent with S-map) and the number of top-performing view*

n. Each view produces independent S-map predictions with exponential distance weighting, and outputs are aggregated via weights proportional to each view's cross-mapping predictive skill. For each time point with missing entries, we build a query attractor state solely from lagged values of the complete series. A weighted local linear regression is fitted on all valid library points, and the optimal θ *is selected to maximize the Pearson correlation between ground truth and imputed values. For each timestamp with missing measurements, we filter valid lag-based attractor views derived from the complete anchor series, run S-map local regression for each qualified view, and compute the weighted ensemble average across top-n views. The optimal combination of* θ *and n is selected to maximize the Pearson correlation coefficient between ground-truth observations and ensemble imputed predictions.*

Reviewer #1 (Remarks on code availability):

n/a

Reviewer #2 (Remarks to the Author):

My earlier comments have been satisfactorily addressed. I am happy to recommend the article, but I suggest a careful reading of the figure captions. For example, I noticed the caption for Figure S19 is not accurate, the panels (c)-(d) do not represent ecological system.

Response: Many thanks for recommending our work.
We have revised the related caption, as follows.

Figure S19. Imputation in multi-dimensional space. (a-c) Imputation results for the 3D ecology system with different embedding dimension, in which variables x and y are incomplete with 50% missingness. (g-l) Similar imputation scenarios are shown for the 3D Lorenz system. We first conduct phase-space reconstruction in a multidimensional space ($E = 10, 20, 30$*) and then implement missing value imputation. The results further demonstrate the reliability of the proposed method.*

Another confusion is with the description of the AR1 process (Figure S6). The same symbol \rho is used for another correlation parameter everywhere else in the paper, where it represents correlation between the imputed and original time series. I suggest you check carefully and change the symbols to avoid confusion.

Response: Thanks for your suggestion. We have replaced the ρ with an alternative notation υ, as follows:

Note: the colored noise is simulated via a typical $AR(1)$ *autoregressive model:* $\eta(t) = \upsilon\eta(t-1) + \varepsilon(t)$ *, where* $\varepsilon(t)$ *is the white noise, and* υ *is the correlation parameter (we set* $\upsilon = 0.8$ *in this work).*

Reviewer #1 (Remarks to the Author):

The authors have done a nice job addressing my earlier suggestions and complaints. Although the cross-mapped gap filling idea is not new, GP is clearly better than simplex/NN for interpolating. This is a substantial piece of work that will almost certainly end up being highly cited.

Response: Thank you very much for your encouraging evaluation. Your valuable comments and suggestions had contributed greatly to improving our work.

Reviewer #2 (Remarks to the Author):

Authors have improved on existing imputation algorithms in the cases where dynamics of the system is fully known. This is slightly different from the case when the system equations are not known in full, but a coupled dependence is assumed between observed and partially observed variables. So in essence, here the idea is to learn a mapping between the full attractor of the system, and partially observed attractor of the system, and then use it for imputation. I believe this work could find application in scenarios when a well-understood system is observed but the exact states are unknown and most of the information about the current and accessible past states is limited. I am surprised to find that some parts of the paper overstate and wrongly claim that this algorithm can help understand "essential system dynamics". They should moderate this claim since at best this algorithm provides a way to find more information about the current state of the system, rather than unfolding the system dynamics or learning equations.

For example, in introduction, lines 83-94, and in lines 117-133, the authors have presented the algorithm as an alternative to exisiting "empirical" ones, such as MVCM. But they have not clarified that the domain of those algorithms by nature is unknown systems, which is very different from their case. On careful reading, I find the comparison rather ill-suited since they work with different information about the system and to compare their performance may not be informative on their suitability for a given situation. This distinction should be clearly stated early in the introduction. Also in the Discussion. For example, the line 433-435 is incomplete: "We demonstrated the imputation performance under the typical assumption that a specific variable is fully observed (e.g., the first variable), whereas all other variables within the system remain incomplete." since there is a bigger assumption that the system dynamics is known. They should state this assumption of known attractor wherever the other assumption of "specific variable is observed etc." is stated so the reader is not misled into believing this is another empirical approach based solely on observed partial data.
I am happy to see that they have moderated this claim and clarified the scope in the rest of the discussion section.
I am happy with the details of the methods and supplementary section.

Response: We greatly appreciate your valuable remarks. We are hoping that you agree that we have moderated the relevant claim and clarified its scope in the Discussion section, and you are satisfied with the details provided in the Methods and Supplementary sections. Thank you indeed for your insightful and constructive comments, which have greatly helped us improve our manuscript.

First, regarding your observation that "essential system dynamics" can be misinterpreted as referring to the system's governing differential equations. We would like to clarify a key conceptual point: FPRM does not require explicit knowledge of system differential equations (For synthetic model-system experiments, the governing differential equations are known for simulation and ground-truth generation; nevertheless, for real-world validation datasets, the underlying system dynamics and governing equations are fully unknown in practice). Its theoretical foundation is Takens' embedding theorem: given time-series outputs from an unknown dynamical system, delay-embedding can reconstruct attractor manifolds that are diffeomorphic to the true underlying attractor, without knowing the governing equations. Critically, FPRM only aims to recover missing historical system states from incomplete observations, instead of inferring, unfolding, or learning the intrinsic dynamical rules or governing equations of the system. We have systematically moderated over-reaching claims throughout the main text and added inline parenthetical clarifications for dynamics. In this manuscript, we focus on key information within dynamics: system's evolution trajectories in the state space, rather than the system's governing equations.
The revised text appears in lines 82-94 of the main manuscript and is reproduced below:
Furthermore, time series observations are intrinsically linked to the underlying dynamics (e.g., system's evolution trajectories in the state space) of complex systems. Missing values in observed time series can pose an obstacle to recovering historical system states and their associated trajectory information [27]. For illustration, consider the classic Lorenz system, a prototypical simplified model of fluid dynamics [28]. Its dynamical flow evolves over time to form a butterfly-like structure in the state space, and system states are directly projected onto the three observed time-series

variables (Figs. 1(d, g)). Consequently, these time series can inversely approximate the historical states of the underlying system (Figs. 1(e, h)), and such reliable reconstruction will facilitate various subsequent downstream dynamical analysis tasks (e.g., dynamical behavior prediction). However, when time-series data contain substantial missing entries, our capacity to reconstruct these historical system states and trajectory information deteriorates (Figs. 1(f, i)).

Second, we would like to clarify a potential misunderstanding: MVCM, S-map and our FPRM all do not require prior knowledge of system governing equations. In our synthetic experiments, differential equations are only used to generate synthetic time-series and ground-truth; these equations are never used as inputs to the FPRM algorithm. FPRM works purely from observed time-series data, as validated by our real-world datasets where governing equations are entirely unknown. Despite sharing Takens' embedding theorem as the theoretical foundation, these methods adopt different modelling paradigms: S-map/MVCM use local nearest-neighbour fitting, whereas FPRM learns diffeomorphic mappings between reconstructed attractor manifolds. Nevertheless, we emphasize that MVCM, S-map and FPRM are all data-driven approaches that operate without prior knowledge of the system's governing dynamical equations, so they share the fundamental setting of working for unknown dynamical systems, which justifies quantitative comparison under controlled benchmark conditions. We have added explanatory text in the Introduction to highlight this point.

The revised text appears in lines 108-113 (Introduction) of the main manuscript and is reproduced below:

To further refine filling accuracy, researchers shift modeling paradigms to encode the fundamental dynamics behind time series. Specifically, S-map and its multi-view extension MVCM [11,25] are classic empirical dynamic modelling tools that operate well without prior knowledge of system governing equations and achieve credible gap-filling results for ecological time series with mild missing entries.

The revised text appears in lines 422-426 (Discussion) of the main manuscript and is

reproduced below:

Despite these static imputation methods, we further consider three representative classical dynamics-aware methods: MVCM [25], S-map [11] and MetaTSI [43]. As time-series reconstruction-based and dynamics-aware paradigms, they are purely data-driven and require no prior knowledge of system's governing equations.

Third, as we clarified above, our FPRM does not require analytically known system dynamics or known true attractor. It does rest upon two critical prerequisites:

(a) At least one variable is fully observed from an underlying system, which can generate a reliable full delay-embedding reconstruction;

(b) The underlying system can be approximated as a deterministic dynamical system satisfying Takens embedding theorem, so that delay-embedding from observed time-series can produce diffeomorphic attractor manifolds. This is a modelling assumption on the nature of the underlying system, rather than requiring prior knowledge of system equations or true attractor geometry.

The first prerequisite is already provided in the Discussion (see lines 594-608), and we have supplemented the second one at lines 582-584 of the Discussion

The underlying system can be approximated as a deterministic dynamical system satisfying Takens embedding theorem, so that reconstruction from each observed time series can produce diffeomorphic attractor manifolds.
